# Integrative Multi‐Omics Mendelian Randomization Highlights Causal Autophagy‐Related Genes for Amyotrophic Lateral Sclerosis

**DOI:** 10.1002/brb3.71366

**Published:** 2026-03-31

**Authors:** Zheng Jiang, Yan‐Lin Ren, Xiao‐Jing Gu, Wei‐Ming Su, Qing‐Qing Duan, Kang‐Fu Yin, Bei Cao, Jing‐Yu Li, Bo Yan, Yong‐Ping Chen

**Affiliations:** ^1^ Department of Neurology West China Hospital Sichuan University Chengdu China; ^2^ Department of Neurology The Second Affiliated Hospital of Chongqing Medical University Chongqing China; ^3^ Department of Pathophysiology West China College of Basic Medical Sciences & Forensic Medicine Sichuan University Chengdu China; ^4^ Mental Health Center, West China Hospital Sichuan University Chengdu China

**Keywords:** amyotrophic lateral sclerosis, autophagy dysregulation, causal genes, Mendelian randomization

## Abstract

**Background:**

Autophagy dysregulation has been implicated in the toxic protein aggregates of amyotrophic lateral sclerosis (ALS). However, the causal relationship between impaired autophagy and ALS remains ambiguous, necessitating further elucidation.

**Methods:**

This Mendelian randomization (MR) study employs a two‐sample design, utilizing genetic instruments to proxy autophagy dysregulation as the exposure and ALS as the outcome. It incorporates summary statistics of ALS (27,205 cases, 110,881 controls), along with data on DNA methylation, RNA splicing, gene expression, and protein abundance quantitative trait loci (QTLs) in both blood and brain tissues (mQTL, sQTL, eQTL, and pQTL, respectively) sourced from European cohorts. *Cis*‐variants situated proximal to or within the 604 autophagy‐related genes, exhibiting robust associations with molecular alterations in autophagy, are employed as instrumental variables. Their causal links with ALS are assessed via summary‐data‐based MR (SMR) analyses, followed by Bayesian colocalization, sensitivity analyses, brain cell‐specific MR analyses, protein–protein interaction (PPI), and druggable analyses.

**Results:**

Consistent evidence supported the causal effects of two lysosome genes (*FNBP1* and *IDUA*), one autophagy core gene (*C9orf72*), and one mitophagy gene (*USP35*) on ALS risk. Specifically, brain *FNBP1* splicing level (OR = 1.18, *p* = 3.38E‐5) and blood *USP35* expression level (OR = 1.17, *p* = 5.94E‐5) were positively associated with higher ALS risk. In contrast, we found strong causal evidence of brain *IDUA* methylation level (OR = 0.96, *p* = 8.36E‐6) and blood *C9orf72* methylation level (OR = 0.55, *p* = 7.59E‐12) with lower ALS risk. Cell‐type‐specific MR analyses, PPI, and druggable analyses further nominated the key brain cell type (astrocytes), potential interaction with known causative genes (*SQSTM1* and *PFN1*), and promising druggability for *FNBP1* in ALS.

**Conclusions:**

This multi‐omics MR study identified causal associations between the regulation of four autophagy‐related genes and ALS risk, shedding light on autophagy‐mediated mechanisms and offering early evidence of novel therapeutic targets for ALS.

## Graphical abstract

Multi‐omics Mendelian randomization reveals causal roles of four autophagy‐related genes (*FNBP1*, *IDUA*, *C9orf72*, *USP35*) in ALS risk, with *FNBP1* and *USP35* increasing risk and *IDUA* and *C9orf72* protective, shedding light on autophagy‐mediated mechanisms and offering early evidence of novel therapeutic targets for ALS.

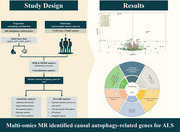

## 1 Introduction

Autophagy is a cellular process that acts as a self‐cleaning mechanism, allowing cells to degrade and recycle their components. It is a highly regulated process essential for cell survival and has been implicated in various physiological and pathological conditions, including aging and neurodegenerative diseases (Klionsky et al. 2021). Autophagy dysfunction is a well‐known amyotrophic lateral sclerosis (ALS) feature, characterized by the accumulation of neurotoxic misfolded proteins. Defective autophagy may lead to the buildup of misfolded proteins and damaged organelles, contributing to neuronal dysfunction and the progression of ALS (Xu et al. 2021). Thus, understanding the precise roles of autophagy in ALS is essential for advancing our knowledge and developing potential therapeutic strategies for the disease.

It is worth noting that autophagy dysregulation represents a multifaceted cellular phenomenon manifesting across a spectrum of pathophysiological states, which pose challenges in accurately determining the timing and spatial localization of its impairment for specific diseases. Many autophagy‐related genes have been implicated in the autophagy‐lysosome process, and genetic variants in these genes may potentially lead to the occurrence of autophagy dysregulation (Kocak et al. 2022). Numerous genetic studies have endeavored to elucidate the causative association between autophagy dysregulation and ALS by exploring rare pathogenic variants and polymorphisms of autophagy‐related genes involved in ALS development (Deng et al. 2017). However, rare pathogenic variants usually account for only a very small fraction of ALS cases, and results from genetic association studies have often been inconsistent due to differences in sample size and ethnic origin. In contrast to these prior mutation‐ or candidate gene‐focused studies, our study adopts a systematic, multi‐omics Mendelian randomization (MR) approach to evaluate whether regulatory genetic variants affecting DNA methylation, RNA splicing, gene expression, or protein abundance in all autophagy‐related genes contribute causally to ALS risk, providing a broader and more unbiased assessment of autophagy's role in ALS pathogenesis.

MR is an analytical approach employing genetic variants as instrumental variables (IVs) to infer potential causal relationships between exposures and outcomes of interest, which mitigates biases arising from unobserved confounders and reverse causality (Skrivankova et al. 2021). Genome‐wide association studies (GWAS) leverage genetic associations with phenotypes utilizing single‐nucleotide polymorphisms (SNPs), while the combination of GWAS statistics with DNA methylation, RNA splicing, gene expression, or protein abundance data has facilitated the delineation of diverse quantitative trait loci (mQTL, sQTL, eQTL, or pQTL, respectively) (Bellenguez et al. 2022; Yang et al. 2021). Moreover, summary‐data‐based Mendelian randomization (SMR) has expanded and refined the concept of MR, enabling the utilization of independent GWAS summary statistics data and QTL data to prioritize potential causal genes derived from GWAS findings (Zhu et al. 2016). By employing SMR in conjunction with the heterogeneity in dependent instruments (HEIDI) test, potential causal relationships were discerned amid the genome's prevalent linkage disequilibrium (LD).

Currently, no MR study has investigated the potential causal association between autophagy dysregulation and ALS risk. The primary objective of this study was to investigate the potential causal association between autophagy dysregulation, as indicated by genetic predispositions in autophagy‐related genes, and ALS risk. In order to establish instruments for autophagy dysregulation from the autophagy‐related genome, we conducted a comprehensive and unbiased analysis that integrated multi‐omics QTL and GWAS data using a two‐sample MR approach. Furthermore, we performed various sensitivity analyses to strengthen the robustness of our findings.

## 2 Methods

**FIGURE 1:**
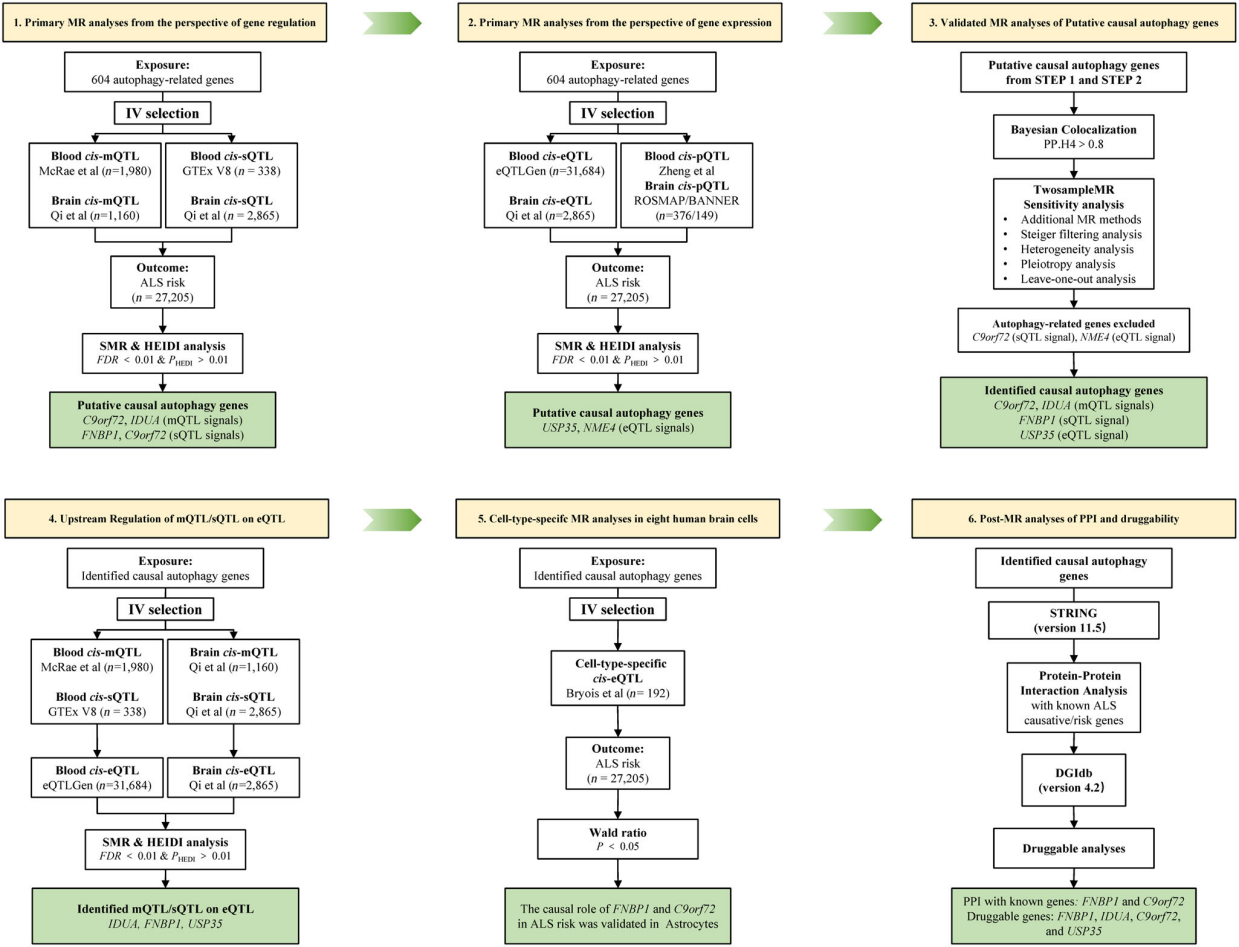
Flow diagram of the MR analyses performed.

Our research strictly followed the STROBE‐MR reporting guideline (see Supporting Information) (Skrivankova et al. 2021). Figure 1 provides an overview of the study's design and the methodology employed in selecting genetic variants and analytical processes. In order to ascertain autophagy dysregulation as indicated by the genetic predisposition in the autophagy‐related genome, we retrieved a list of 604 established autophagy‐related genes comprehensively summarized by an original publication (Bordi et al. 2021). The main categories included MTOR and upstream pathways (135 genes), autophagy core (197 genes), autophagy transcription factors (68 genes), mitophagy (80 genes), docking and fusion (22 genes), and lysosome (162 genes) and lysosome‐related genes (34 genes) (for details, see Table S1).

### 2.1 Data Source

#### 2.1.1 Exposure Source

All SNPs included in the initial analysis had at least a suggestive *p* value < 5 × 10^−8^. SNPs located within the human major histocompatibility complex region at chromosome 6: 26–34 Mb (GRCh37/hg19) were excluded considering their complex LD structure (Zheng et al. 2020).

##### 2.1.1.1 mQTL

Blood *cis*‐mQTL instruments robustly associated with autophagy gene methylation were extracted using summary data from a meta‐analysis of two cohorts (*n* = 1980) (McRae et al. 2018). As a result, 1601 SNPs were selected, corresponding to 1902 autophagy‐related DNA methylation CpG sites.

The brain *cis*‐mQTL database originated from a 2018 publication by Qi et al. (2018). In this study, the authors conducted a meta‐analysis of *cis*‐mQTLs using data from three different datasets: 468 cortical brain region samples from the ROSMAP study (Ng et al. 2017), 166 fetal brain samples by Hannon et al. (2016), and 526 frontal cortex region samples (Jaffe et al. 2016). This resulted in a final sample size of 1160 brain samples and nearly 6 million *cis*‐mQTLs. Consequently, we identified 1623 SNPs corresponding to 1929 autophagy‐related DNA methylation CpG sites.

##### 2.1.1.2 sQTL

Blood *cis*‐sQTL instruments robustly associated with autophagy RNA splicing abundance were extracted using summary data from GTEx V8 (*n* = 338) (Garrido‐Martín et al. 2021). In total, 237 SNPs of 328 autophagy‐related splicing probes were selected as instruments for the MR.

Human brain *cis*‐sQTL summary data were obtained from Qi et al. (2022), which contains sQTL data from 2865 human brain transcriptomes. As a result, 1146 SNPs of 1591 autophagy‐related splicing probes were selected.

##### 2.1.1.3 eQTL

Blood eQTL instruments for autophagy‐related genes were obtained using the *cis*‐eQTL summary statistics from the eQTLGen Consortium (https://www.eqtlgen.org/cis‐eqtls.html). The eQTLGen Consortium provides information on 10,317 trait‐associated SNPs from 31,684 individuals (Vosa et al. 2021). It is important to note that variants associated with gene expression levels of X and Y chromosomes were not included in the eQTLGen dataset. For the *cis*‐eQTL analysis, we selected 489 SNPs linked to the expression of 489 autophagy‐related transcripts.

To generate brain *cis*‐eQTL instruments for autophagy‐related genes, we used the BrainMeta v2 dataset (Qi et al. 2022), which contains eQTL data from 2865 human brain transcriptomes. As a result, 347 SNPs associated with the expression of 347 autophagy‐related transcripts were selected.

##### 2.1.1.4 pQTL

Blood *cis*‐pQTL instruments for genetic variants associated with the expression of autophagy‐related proteins were selected from the study by Zheng et al. (2020), which integrated five published proteome datasets (Suhre et al. 2017; Yao et al. 2018; Folkersen et al. 2020; Sun et al. 2018; Emilsson et al. 2018), and 23 SNPs robustly associated with 23 autophagy‐related protein expressions were selected.

The brain *cis*‐pQTL data were derived from two datasets: the Religious Orders Study (ROS) or the Rush Memory and Aging Project (MAP) dataset (referred to as the ROSMAP dataset), consisting of dorsolateral prefrontal cortex (dlPFC) samples from 376 subjects (Beach et al. 2015), and the Banner dataset comprising dlPFC samples from 152 individuals collected by the Banner Sun Health Research Institute (Beach et al. 2015). Finally, 32 SNPs associated with autophagy‐related protein expressions were selected for analysis.

### 2.2 Outcome Source

The outcome GWAS were performed in 29,612 patients with ALS and 122,656 controls, but we only used the summary results conducted in European ancestry populations (27,205 cases, 110,881 controls) (van Rheenen et al. 2021).

Table 1 summarizes QTL and GWAS data used in this research, and details on the study design have been described in the original paper. The present study only involved publicly available GWAS summary statistics, so informed consent and ethical approval were not required.

**TABLE 1:** Information on QTL and GWAS datasets.

Type of dataset	Data subtype	Resource	Sample size	Sample Age Group	Brain region stratification	Reference	Download Site
QTL	Brain *cis‐*mQTL	Brain‐mMeta mQTL summary data	1160	Adults	Yes	Qi et al. (2018)	https://yanglab.westlake.edu.cn/data/SMR/Brain‐mMeta.tar.gz
	Blood *cis‐*mQTL	McRae et al. mQTL summary data	1980	Adults (including older adults in LBC)	/	Y. Wu et al. (2018)	https://yanglab.westlake.edu.cn/data/SMR/LBC_BSGS_meta_lite.tar.gz
	Brain *cis‐*sQTL	BrainMeta v2 sQTL summary data	2865	Adults	Yes	Qi et al. (2022)	https://yanglab.westlake.edu.cn/data/SMR/BrainMeta_cis_sqtl_summary.tar.gz
	Blood *cis‐*sQTL	V8 release of the GTEx sQTL summary data	338	Adults (21–70 years)	/	GTEx Consortium (2020)	https://yanglab.westlake.edu.cn/data/SMR/GTEx_V8_cis_sqtl_summary_lite.tar
	Brain *cis‐*eQTL	BrainMeta v2 eQTL summary data	2865	Adults	Yes	Qi et al. (2018)	https://yanglab.westlake.edu.cn/data/SMR/BrainMeta_cis_eqtl_summary.tar.gz
	Blood *cis‐*eQTL	eQTLGen Consortium	31,684	Adults	/	Vosa et al. (2021)	https://www.eqtlgen.org/cis‐eqtls.html
	Brain *cis‐*pQTL	ROSMAP Consortium	376	Older adults	Only dorsolateral prefrontal cortex	Robins et al. (2021)	https://adknowledgeportal.org/
		BANNER Consortium	149				
	Blood *cis‐*pQTL	Five published proteome datasets integrated by Zheng et al. (2020) (PMID: 32895551)	1000	Adults (23–81 years)	/	Suhre et al. (2017)	https://www.nature.com/articles/ncomms14357#MOESM1569
			3200	Older adults (> 65 years)	/	Emilsson et al. (2018)	https://www.science.org/doi/10.1126/science.aaq1327?url_ver=Z39.882003&rfr_id=ori:rid:crossref.org&rfr_dat=cr_pub%20%200pubmed
			3301	Adults (blood donors)	/	Sun et al. (2018)	http://www.phpc.cam.ac.uk/ceu/proteins/
			6861	Adults (mean age 50)	/	Yao et al. (2018)	https://www.nature.com/articles/s41467‐018‐05512‐x#additional‐information
			21,758	Adults (middle and old age)	/	Folkersen et al. (2020)	https://www.nature.com/articles/s42255‐020‐00287‐2#Sec35
	*cis*‐eQTL of Eight Brain Cells	Published paper	192	Adults	No	Bryois et al. (2022)	https://zenodo.org/record/7276971
GWAS summary	ALS	Project MinE	case: 27,205, control: 110,881	Adults	/	van Rheenen et al. (2021)	https://www.projectmine.com/research/download‐data/

### 2.3 Statistical Methods

The main analytical process consisted of SMR, colocalization, and sensitivity analyses.

#### 2.3.1 SMR Analysis

MR necessitates fulfilling three fundamental assumptions. Assumption 1: The genetic instruments should exhibit associations with the exposures, that is, QTL(s). Assumption 2: The genetic instruments should exclusively influence the outcomes, that is, ALS, through their effects on the exposures, thereby avoiding any instances of horizontal pleiotropy. Assumption 3: The genetic instruments should not be linked to any confounding factors that could impact the association between the exposure and outcome. To extend the concept of MR, the SMR method was devised to examine the pleiotropic association between exposure (DNA methylation, RNA splicing, gene expression, or protein abundance) and outcome (ALS risk) (Zhu et al. 2016). To satisfy the MR assumptions, we computed the causal association as *β*_autodys‐ALS_ = *β*_SNP‐ALS_/*β*_SNP‐autodys_. *β*_autodys‐ALS_ represents the estimated effect size of autophagy dysregulation on ALS, whereas *β*_SNP‐autodys_ denotes the estimated effect size of the genetic variant on autophagy dysregulation (the association between the genetic variant and the exposure trait), and *β*_SNP‐ALS_ denotes the estimated effect size of the genetic variant on ALS (the association between the same genetic variant and the outcome trait). The HEIDI test (*p*_HEIDI_ > 0.01) was utilized to exclude significant SMR associations due to linkage rather than causality or pleiotropy (see Supporting Information).

In this study, we conducted SMR analyses utilizing the Linux version 1.3.1 of the SMR software by default settings. To assess the impact of autophagy dysregulation on ALS risk, we acquired odds ratio (OR) estimates by OR_autodys‐ALS_ = exp(*β*_autodys‐ALS_), in which OR represents the odds ratio estimate per a 1‐ln increment in autophagy‐related molecular alteration levels, and exp denotes the base of the natural logarithm. We defined significance at a false discovery rate (FDR) of 0.01 for each SMR analysis (see Supporting Information).

### 2.4 Colocalization Analysis

The HEIDI test, which utilizes an external reference to estimate LD, is one method employed for colocalization analysis. To further refine the results, we conducted a Bayesian test to assess the colocalization of two traits and estimate the posterior probability of shared variants (Giambartolomei et al. 2014). We retrieved all SNPs within a 100 kb range upstream and downstream of the leading SNPs for colocalization analysis. This analysis aimed to evaluate the posterior probability of H4 (PP.H4), with a cutoff of PP.H4 > 0.8 commonly used to indicate strong evidence of colocalization between the GWAS and QTL associations (see Supporting Information).

### 2.5 Sensitivity Analysis

Following SMR analyses, sensitivity analyses were conducted using other MR methods, namely inverse variance weighting (IVW), weighted median, MR Egger, simple mode, and weighted mode, with default parameters. Each method originated from various assumptions on instrument validity estimates the causal effect, thereby providing robust evidence for primary findings. Additionally, Steiger analyses were used to confirm that the proposed instruments were directly associated with the outcome or effect estimate directionality (Hemani et al. 2017). To test for heterogeneity (*Q* test *p <* 0.05) and horizontal pleiotropy (MR‐Egger intercept *p* < 0.05), we employed Cochran's *Q* (Higgins et al. 2003) and MR Egger intercept test (Burgess and Thompson 2017), respectively. Furthermore, we conducted a leave‐one‐out analysis within the IVW method to assess the influence of individual variants on the observed association (Burgess et al. 2017). When only one genetic variant was included in the analysis, the simplest approach, the Wald ratio estimate, was used to obtain the causal estimate for the genetic variant representing the exposure on the outcome (Burgess et al. 2017). The *F*‐statistic was employed to measure the strength of the variants, with a value > 10 indicating a strong MR instrument (Skrivankova et al. 2021).

Colocalization and sensitivity analyses were mainly performed using the packages TwoSampleMR (0.5.6) and coloc (5.2.0) of the statistical software R (4.1.3).

### 2.6 Phenome‐Wide Scan of Identified Genetic Variants

To further evaluate horizontal pleiotropic effects, we conducted a comprehensive search for identified genetic variants surviving the above analyses using the PhenoScanner database (Kamat et al. 2019), which incorporates over 5000 GWAS at the time of this investigation. We performed a phenome‐wide scan (PheWAS) by considering all identified genetic variants related to disease traits, and an SNP was considered pleiotropic when satisfying the following criteria: (1) the SNPs exhibited the same effect allele as our findings, (2) the association reached genome‐wide significance (*p* < 5 × 10^−8^), (3) the absolute value of the effect size (*β*) was over 0.01, and (4) the ethnic population was of European descent.

### 2.7 Regulatory Relationships Between Epigenetic and Transcriptional Layers

DNA methylation and RNA splicing are known to influence gene expression and protein abundance through transcriptional and posttranscriptional mechanisms (J. Wang et al. 2013; Kornblihtt et al. 2013). To provide additional mechanistic context for the identified QTL–ALS associations, we conducted SMR analyses to examine whether ALS‐associated DNA methylation and RNA splicing signals were also associated with gene expression or protein abundance. These analyses were intended as exploratory investigations to characterize potential regulatory relationships between different molecular layers, rather than to establish formal causal hierarchies linking genetic variants to ALS risk.

### 2.8 Cell‐Type‐Specific MR Analyses of Identified Causal Autophagy Genes

To nominate the potential brain cell types for identified causal autophagy genes, we further performed MR analyses using *cis*‐eQTL data (Bryois et al. 2022) of eight brain cells (astrocytes, endothelial cells, excitatory neurons, inhibitory neurons, oligodendrocytes, OPCs, pericytes, and microglia). The IV selection criteria for identified genes were the same as those in the sensitivity analyses.

### 2.9 Protein–Protein Interaction Analysis

To investigate the interactions between the autophagy‐related genes in this study and the known ALS causative/risk genes extracted from the Amyotrophic Lateral Sclerosis Online Database (Abel et al. 2012), we explored the protein–protein interaction (PPI) network between them by using the Search Tool for the Retrieval of Interacting Genes (STRING) database version 11.5 (https://string‐db.org/) (Szklarczyk et al. 2019). We input gene symbols into the STRING database and set the minimum required interaction score at 0.4 to ensure confidence in the results.

### 2.10 Druggable Analysis

Genes with QTL signals in blood or brain that passed SMR, sensitivity, and colocalization analyses were interrogated for druggability in the Drug Gene Interaction Database (DGIdb) (version 4.2) (https://www.dgidb.org/) (Freshour et al. 2021). DGIdb employs various sources of information, including expert curation, text‐mining, and data on drug–gene interactions from databases such as DrugBank, PharmGKB, ChEMBL, and Drug Target Commons, to prioritize genes with potential druggability and assess gene function (Freshour et al. 2021).

## 3 Results

### 3.1 MR Analysis of Autophagic *Cis*‐mQTLs and ALS

**FIGURE 2:**
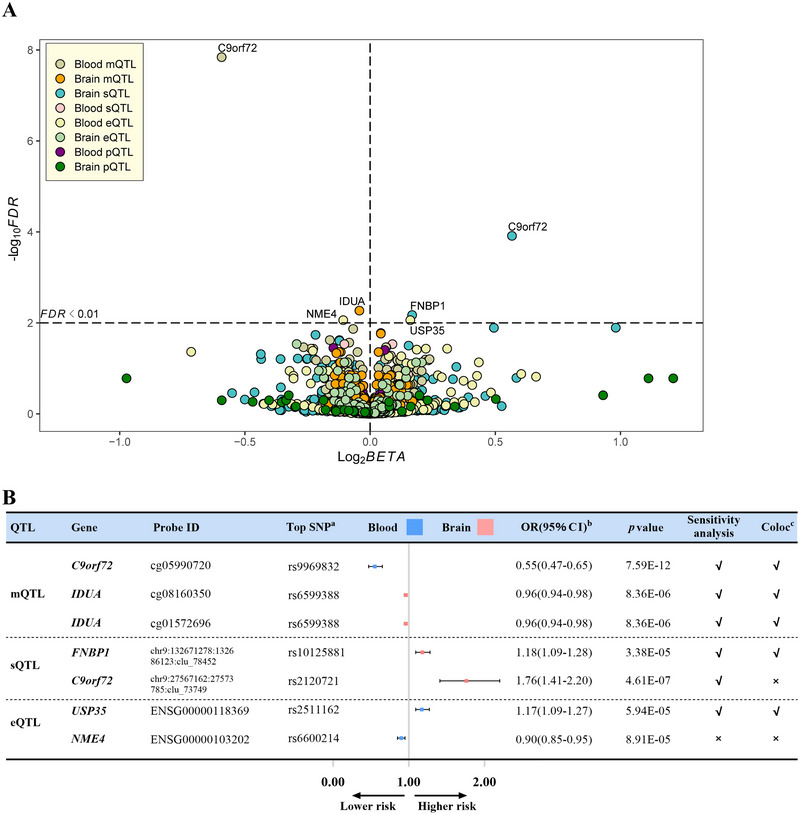
Mendelian randomization results for the association between the autophagy‐related genes and ALS risk. (A) Volcano plot displaying the log2 Beta (*x* axis) against the −log10 FDR *p* value (*y* axis) for the association between the autophagy‐related QTLs and ALS risk. (B) Forest plot for SMR estimates for the outcomes reaching 1% FDR significance without evidence of heterogeneity as determined by the HEIDI test. ^a^Different probes may detect the same SNP. ^b^Odds ratios were calculated by the expectation of the causal estimate (*β* coefficient). ^c^Colocalization indicates PP.H4 between QTLs and ALS risk. PP.H4 > 0.8 is the well‐applied cutoff for the evidence of colocalization.

For the causal association between the DNA methylation of the autophagy‐related genes and ALS, SMR analyses (FDR *p*_SMR_ < 0.01) and HEIDI tests (*p*_HEIDI_ > 0.01) were performed. After correction for multiple testing, we identified one association signal across one unique genetic locus for ALS in blood and two association signals across one unique genetic locus for ALS in brain (Figure 2 and Table S2). The sensitivity and colocalization analyses supported the SMR analyses (Tables S2 and S3). In concrete, we found strong causal evidence of blood *C9orf72* methylation level (OR per SD, 0.55; 95% CI, 0.47–0.65; *p* = 7.59E‐12), as well as brain *IDUA* methylation level (OR per SD, 0.96; 95% CI, 0.94–0.98; *p* = 8.36E‐6) with lower ALS risk.

### 3.2 MR Analysis of Autophagic *Cis*‐sQTLs and ALS

For the causal association between the RNA splicing of the autophagy‐related genes and ALS, SMR analyses (FDR *p*_SMR_ < 0.01) and HEIDI tests (*p*_HEIDI_ > 0.01) identified two association signals across two unique genetic loci for ALS in the brain after correction for multiple testing (Figure 2 and Table S2). The sensitivity analyses supported the same associations as the SMR analyses (Table S3). Specifically, brain *FNBP1* splicing level was positively associated with higher ALS risk (OR per SD, 1.18; 95% CI, 1.09–1.28; *p* = 3.38E‐5). However, colocalization analyses suggested that the causal effect of *C9orf72* on ALS risk may be confounded by LD.

### 3.3 MR Analysis of Autophagic *Cis*‐eQTLs and ALS

For the causal association between the RNA expression of the autophagy‐related genes and ALS, SMR analyses (FDR *p*_SMR_ < 0.01) and HEIDI tests (*p*_HEIDI_ > 0.01) identified two association signals across two unique genetic loci for ALS in blood after correction for multiple testing (Figure 2 and Table S2). Specifically, blood *USP35* expression level was positively associated with higher ALS risk (OR per SD, 1.17; 95% CI, 1.09–1.27; *p* = 5.94E‐5) (for details, see Figure 2, Table S2, and Table S3). However, the sensitivity and colocalization analyses failed to support the causal effect of *NME4* expression abundance indicated by rs6600214 on ALS risk.

### 3.4 MR Analysis of Autophagic *Cis*‐pQTLs and ALS

For the causal association between the protein abundance of the autophagy‐related genes and ALS, SMR analyses (FDR *p*_SMR_ < 0.01) and HEIDI tests (*p*_HEIDI_ > 0.01) were performed. Only 23 proposed autophagy‐related SNPs in blood and 32 in the brain were extracted from *cis*‐pQTLs. However, no association signals survived the correction for multiple testing.

### 3.5 Summary of SMR, Sensitivity, and Colocalization Analyses

**FIGURE 3:**
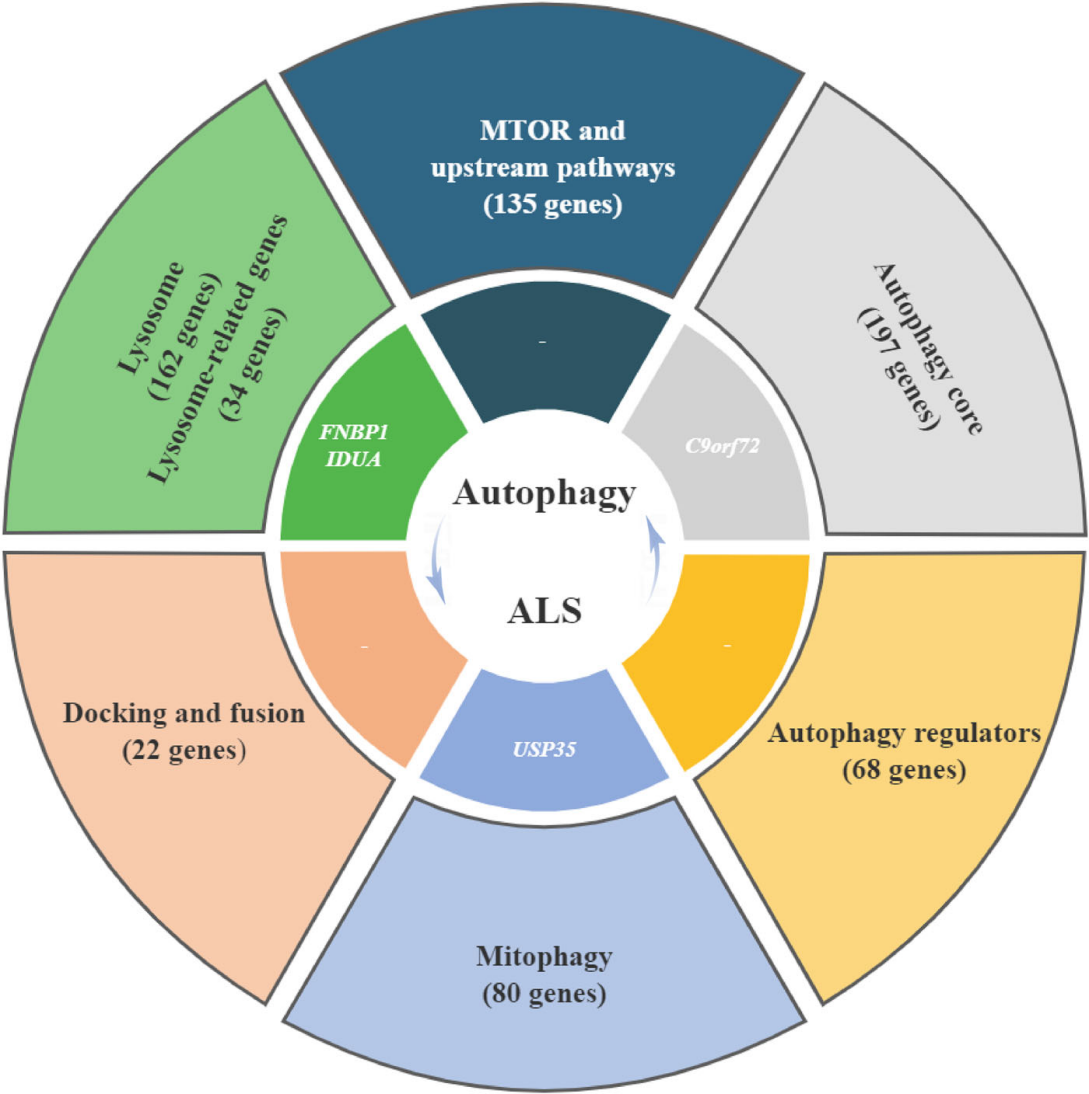
Causal autophagy‐related genes for amyotrophic lateral sclerosis.

First, we identified potential causal relationships between five autophagy‐related genes and ALS after SMR analyses. Subsequently, four of them survived the predefined sensitivity and colocalization analyses. Finally, we found five QTL signals from two lysosome genes (*FNBP1* and *IDUA*), one autophagy core gene (*C9orf72*), and one mitophagy gene (*USP35*) in either blood or brain that have causal effects on ALS risk (Figure 3). Overall, four identified autophagy‐related genes manifest their causal influence on ALS risk through mQTL, sQTL, and eQTL signals.

### 3.6 Phenome‐Wide Scan of Identified Genetic Variants

We performed a PheWAS of the included genetic variants using the PhenoScanner database to systematically identify potential associations with secondary phenotypes (Table S4). The RNA splicing variants of *FNBP1* and *C9orf72* that were causally associated with ALS were not found to be related to any secondary phenotype, supporting the robustness of their inferred causal effects on ALS risk.

In contrast, the DNA methylation variant of *IDUA* and the gene expression variant of *USP35*, both of which showed causal associations with ALS, were also associated with several additional phenotypes (Table S4). Specifically, the *IDUA* methylation variant was associated with Parkinson's disease (PD), while the *USP35* expression variant was associated with whole‐body and limb impedance measures as well as age at menarche. These findings suggest the presence of potential pleiotropic effects for *IDUA* and *USP35*, which warrant further interpretation regarding their biological relevance and the nature of pleiotropy involved.

### 3.7 Regulatory Relationships Between Epigenetic and Transcriptional Layers

Furthermore, DNA methylation or RNA splicing can influence gene expression. Therefore, we also performed SMR analyses on the causal association between autophagy‐related gene methylation or RNA splicing and RNA expression by mapping the mQTLs or sQTLs to eQTLs through shared genetic variants. After correction for multiple testing and the HEIDI test, we obtained the gene list for the autophagy gene expression regulated by DNA methylation CpG or RNA splicing sites (Table S5). For the potential causal genes identified above, SMR results showed that the methylation of *USP35*, which was regulated by rs2512525, was associated with blood *USP35* expression level (Table S5).

### 3.8 Cell‐Type‐Specific MR Analyses of Identified Causal Autophagy‐Related Genes

To support the role of identified causal autophagy genes in specific brain cells, cell‐type‐specific MR analyses were further performed. As a result, the expression level of *FNBP1* in astrocytes was negatively associated with ALS risk (OR per SD, 0.88; 95% CI, 0.81–0.94; *p* = 3.99E‐4). In contrast, the expression level of *C9orf72* in astrocytes was positively associated with ALS risk (OR per SD, 1.25; 95% CI, 1.15–1.36; *p* = 1.33E‐7) (Table S6). Due to the limited number of independent SNPs available in current cell‐type‐specific eQTL datasets, HEIDI and Bayesian colocalization analyses were not applicable; thus, these results were considered supportive rather than confirmatory.

### 3.9 PPI Analyses

**FIGURE 4:**
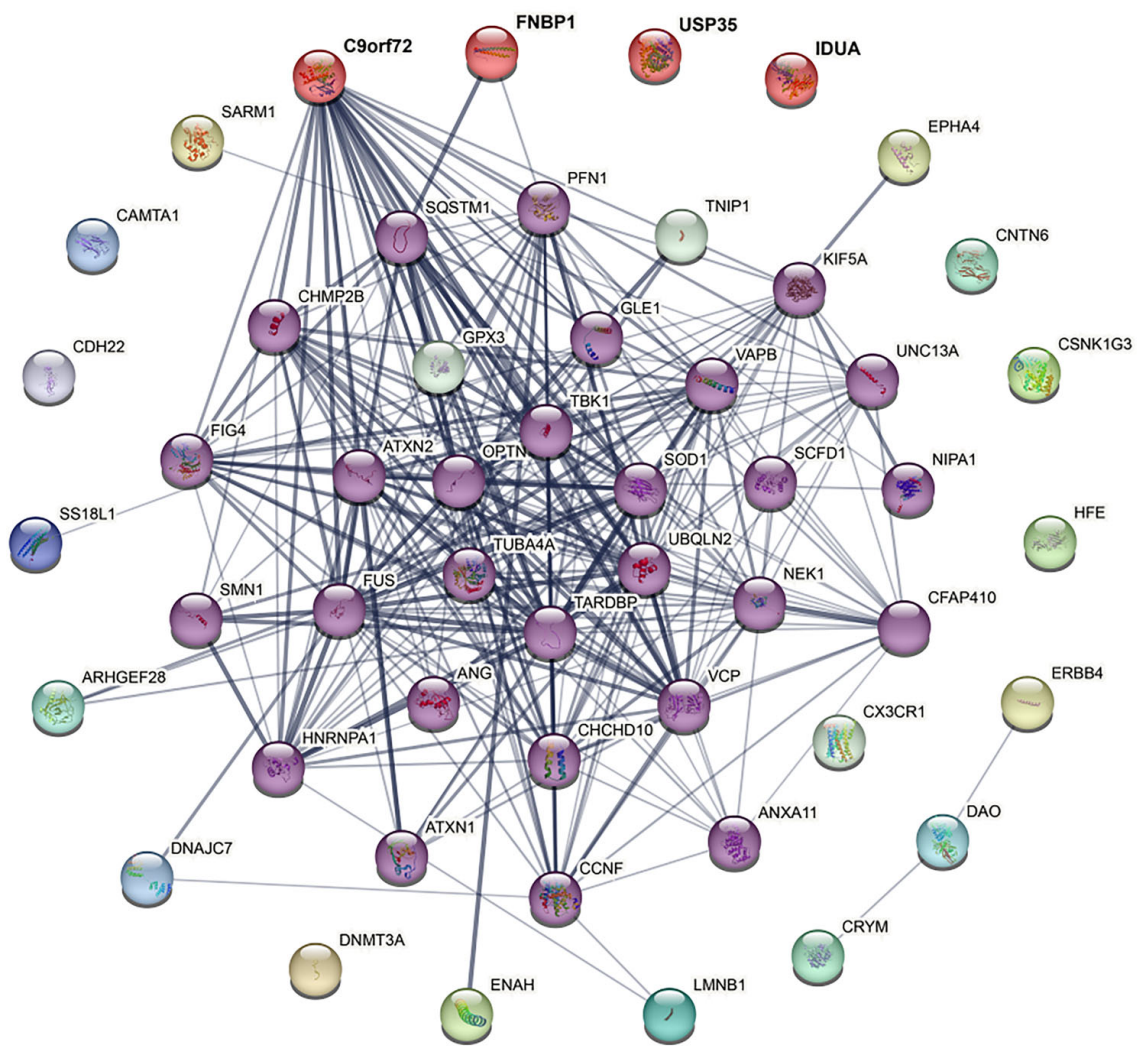
The protein–protein interaction network between the identified causal autophagy‐related genes and known ALS causative/risk genes. The nodes of red color mean the potential causal genes identified in this study, while the nodes of purple color represent the known ALS causal/risk genes interacting with the identified genes. Line thickness indicates the confidence of interaction.

The PPI network revealed interactions between two autophagy‐related genes (*FNBP1* and *C9orf72*) and multiple ALS causative/risk genes (Figure 4 and Table S7). Specifically, *FNBP1* interacts with *SQSTM1 and PFN1*, while the well‐known ALS causative *C9orf72*, also identified in this study, demonstrated various interactions with other ALS causative/risk genes, such as *SOD1* and *TARDBP* (Figure 4).

### 3.10 Druggable Analyses

We next evaluated whether four autophagy‐related genes (*FNBP1*, *IDUA*, *C9orf72*, and *USP35*) with QTL signals robustly associated with ALS risk were potentially druggable by using DGIdb (Freshour et al. 2021). Although no evidence of drug–gene interactions was found, druggability analyses indicated that all of them were druggable (Table S8), which will contribute to prioritizing novel ALS drug targets.

## 4 Discussion

In this study, an integrative analytical pipeline combining SMR, colocalization, sensitivity analysis, regulatory relationship analyses involving eQTLs, cell‐type‐specific MR analysis, PPI analysis, and druggability analysis was employed to systematically explore the potential causal relationship between autophagy dysregulation and ALS risk. Specifically, we found two lysosome genes (*FNBP1* and *IDUA*), one autophagy core gene (*C9orf72*), and one mitophagy gene (*USP35*) in either blood or brain that have causal effects on ALS risk. These genes may mediate the risk of ALS by modulating either RNA splicing, DNA methylation, or RNA expression. Especially, *FNBP1* manifested no horizontal pleiotropy associated with any secondary traits and was also validated in cell‐specific MR analyses, and the PPI analyses further provided evidence for its gene‐gene interaction with the known causative/risk genes of ALS. In addition, this gene has been identified as clinically actionable through druggability analyses. Overall, our study is fundamental as an attempt to fill the gaps in our understanding of causally involved autophagy‐related genes and shed light on the critical components of autophagy in the pathogenesis of ALS.

Although a series of causative or risk genes for ALS have been discovered over time, they can only account for a small proportion of ALS cases (Chen et al. 2022). Currently, researchers have a minimal understanding of the underlying mechanisms driving the disease in most ALS patients. Prior investigations have implicated autophagy as a crucial player in ALS pathology; however, the exact mechanisms by which autophagy contributes to the disease remain incompletely elucidated. In the most recent GWAS (van Rheenen et al. 2021), the genetic loci associated with ALS were enriched with genes involved in autophagy and degradation of (misfolded) proteins. Using multi‐omics data from both blood and brain, our analysis identified four autophagy‐related genes potentially involved in the pathogenesis of ALS, including one known causative gene, *C9orf72*, as well as three promising genes, *FNBP1*, *IDUA*, and *USP35*.

The *C9orf72* gene has been established as a known pathogenic gene associated with ALS, expanded repeats of which account for ∼40% of familial and ∼7% of sporadic ALS in Europeans (Akçimen et al. 2023). Three primary hypothesized mechanisms driving *C9orf72*‐related ALS involve C9orf72 protein haploinsufficiency and toxic gain of function through sequestration of RNA‐binding proteins and dipeptide repeat proteins formed through non‐canonical RNA translation (Gagliardi et al. 2020). Although the pathogenic mechanisms by which repeat expansions in the *C9orf72* gene are causing ALS are not fully understood, many studies indicated that dysregulation of autophagy has been implicated in *C9orf72*‐related ALS (Beckers et al. 2021). Consistent with previous studies, our research reaffirmed the link between *C9orf72* and ALS risk. Importantly, we indicated the protective mechanism by which *C9orf72* influences ALS risk partially through blood DNA methylation, aligning with prior findings that *C9orf72* hypermethylation may protect against ALS repeat expansion‐associated pathology (E. Y. Liu et al. 2014). In addition, cell‐type‐specific MR analyses validated the role of *C9orf72* in astrocytes, which is an important brain cell type for ALS (Rajpurohit et al. 2020; Allen et al. 2019; Madill et al. 2017; Sugiyama et al. 2013). However, at the *C9orf72* locus, we cannot fully exclude the possibility that some cis‐QTL variants are in LD with the pathogenic hexanucleotide repeat expansion, as previously demonstrated in ALS GWAS (e.g., van Rheenen et al. 2021). Nevertheless, our findings were based on QTL instruments that directly proxy regulatory changes (e.g., DNA methylation), which are less likely to be entirely explained by repeat tagging. Future studies incorporating long‐read sequencing or direct genotyping of *C9orf72* expansions will be important to further clarify this issue.

*FNBP1* encodes a member of the formin‐binding‐protein family, which mainly exists in the lysosome and cytosol. It plays a vital role in clathrin‐mediated endocytosis (Shimada et al. 2007). Although *FNBP1* has been preliminarily reported as a risk gene for ALS by a multiethnic meta‐GWAS (Nakamura et al. 2020), its role was not clarified in ALS. A recent large‐scale transcriptomics analysis of human postmortem ALS spinal cords identified that oligodendrocytes were exclusively assigned as a putative cell type for *FNBP1* (Humphrey et al. 2023). In this study, *FNBP1* is proven to affect ALS through RNA splicing, which is consistent with our findings of a positive relationship between brain *FNBP1* splicing level and ALS risk. More importantly, the sQTL data we analyzed are from the brain, which expands the potential pathophysiologic role of *FNBP1* from the spinal cord to the brain, another key affected region in ALS. Furthermore, our study validates the protective role of *FNBP1* expression level in astrocytes by cell‐type‐specific MR analyses, supported by the significant downregulation of this gene in animal models (W. Liu et al. 2020) as well as sporadic ALS patients (Andrés‐Benito et al. 2017). In the PPI analyses, *FNBP1* is found to interact with an ALS‐associated gene *SQSTM1* and *PFN1*, both of which may act in the formation of pathological protein aggregates in ALS models (Brady et al. 2011; C.‐H. Wu et al. 2012). Notably, the PheWAS of identified genetic variants for *FNBP1* found no pleiotropic ones associated with the secondary traits, and the druggability analyses have assigned this gene to be clinically actionable. Overall, our results firstly indicated that *FNBP1* may be a robust autophagy‐related gene with causal links with ALS in the brain.

*IDUA* and *USP35* emerge as two novel genes discovered in our study, albeit with relatively limited supporting evidence for their involvement in ALS. *IDUA* encodes the enzyme alpha‐l‐iduronidase, which is responsible for the degradation of glycosaminoglycans in lysosomes. Mutations in the *IDUA* gene cause mucopolysaccharidosis type I, a lysosomal storage disease (Poletto et al. 2019). Although this gene has not been reported to be associated with ALS, our study provides preliminary evidence indicating a possible association between *IDUA* and ALS risk through DNA methylation in the brain. The dysregulation of IDUA‐mediated lysosomal degradation pathways may contribute to the accumulation of protein aggregates and impaired autophagic flux observed in ALS. The *USP35* gene encodes a member of the peptidase C19 family of ubiquitin‐specific proteases, which catalyze the removal of ubiquitin proteins from target proteins. USP35 has been involved in several cellular processes, including the regulation of PARK2‐mediated mitophagy (Y. Wang et al. 2015) and Aurora B stability (Park et al. 2018). Our findings, which indicate a positive association between blood *USP35* gene expression and ALS risk, differ from a recent study that observed downregulation of USP35 in ALS‐affected neurons (Farrawell et al. 2020), highlighting the complexity of its role across different cell types.

The PheWAS analysis revealed that genetic variants instrumenting *IDUA* and *USP35* were associated with several secondary phenotypes, raising the possibility of pleiotropic effects. However, the presence of such associations does not necessarily imply horizontal pleiotropy that would violate MR assumptions. For *USP35*, the observed associations with whole‐body and limb impedance measures may reflect vertical pleiotropy, whereby altered gene expression influences body composition–related traits that could lie upstream of ALS risk. Such a mechanism would be compatible with a causal pathway linking *USP35* expression to ALS rather than indicating an alternative confounding pathway. Similarly, the association between *IDUA* methylation and PD does not imply that PD mediates ALS risk. Instead, *IDUA* may exert independent effects on multiple neurodegenerative phenotypes through shared lysosomal or neurodegeneration‐related biological pathways. This pattern is consistent with biological pleiotropy rather than horizontal pleiotropy driven by distinct causal routes. Therefore, while pleiotropic associations were observed for *IDUA* and *USP35*, these findings should be interpreted cautiously and do not necessarily invalidate the inferred causal relationships with ALS. Future studies incorporating multivariable MR or pathway‐specific analyses may further clarify the underlying mechanisms.

The present study has several key strengths. First, it adopts a comprehensive multi‐omics MR approach to investigate the causal relationship between autophagy dysregulation and ALS. Additionally, integrating multiple molecular traits, that is, mQTL, sQTL, eQTL, and pQTL, provides a robust framework for causal inference. Second, the study includes various molecular traits specific to blood and brain tissues. This tissue‐specific analysis enables a more refined assessment of autophagy‐related molecular changes in tissues relevant to ALS pathology, while acknowledging that bulk brain and blood datasets may not fully capture disease‐relevant regulatory effects. Third, the study employs multiple MR methods, including the SMR approach, Bayesian colocalization, sensitivity analyses, regulatory relationship analyses involving eQTLs, cell‐type‐specific MR analyses, PPI, and druggable analyses. These rigorous analyses ensure the reliability, robustness, and consistency of the findings while offering valuable insights into potential pathways and therapeutic targets involved in the pathogenesis of ALS.

This study has several limitations as well. First, the relatively small number of autophagy‐related pQTLs likely reflects the limited coverage of current plasma proteomic datasets, which underrepresent low‐abundance intracellular proteins, rather than a true absence of protein‐level regulation. Moreover, a key limitation is the use of QTL data that sometimes conflates brain regions. While some datasets (e.g., GTEx Consortium 2020) distinguish partitions like the amygdala and cortex, others aggregate cortical areas or focus solely on the dlPFC (e.g., Robins et al., 2021). This may miss heterogeneity in gene expression, methylation, and splicing, potentially limiting insights into ALS mechanisms affecting motor neurons. We prioritized these datasets for sample size and statistical power, given the scarcity of ALS‐specific resources. Future studies incorporating single‐cell or motor neuron–focused QTL resources (e.g., PsychENCODE) may provide greater anatomical and cellular resolution. In addition, our analyses of regulatory relationships between DNA methylation, RNA splicing, and gene expression were not designed to establish definitive causal hierarchies from genetic variation to ALS risk. Ideally, such hierarchies would be evaluated by systematically testing whether mQTL or sQTL signals exert downstream effects on gene expression that subsequently mediate ALS risk. However, the limited number of variants instrumenting DNA methylation and RNA splicing for the identified genes constrained the statistical power for such analyses. Consequently, findings such as the association between a *USP35* mQTL and *USP35* expression, in the absence of a direct association between the mQTL and ALS risk, should be interpreted as providing a supportive mechanistic context rather than evidence of a complete causal pathway. These results suggest regulatory coherence across molecular layers but do not imply that upstream epigenetic variants independently drive ALS risk. Furthermore, for most autophagy‐related genes, only a single cis‐acting SNP was available as an IV, limiting our ability to perform multiple sensitivity analyses and reducing confidence in fully excluding horizontal pleiotropy. Some QTL datasets (e.g., ROSMAP) included both diseased and healthy individuals; while this increases power, it may also introduce potential bias due to disease‐related expression variation. Finally, the possibility of weak instrument bias cannot be completely ruled out, particularly for genes with low expression levels or limited QTL effect sizes. To mitigate this, we calculated *F*‐statistics for all instruments and excluded those with *F* < 10, but residual bias remains possible.

## 5 Conclusion

In summary, our results support the idea that autophagy dysregulation plays an essential role in the pathophysiology of ALS. Further work on the causal autophagy‐related genes for ALS, especially *FNBP1*, is needed to better understand the links between perturbed autophagy function and the neuronal death involved in ALS.

## Author Contributions

Z.J. and Y.P.C. conceived and designed the study. Z.J. contributed to the statistical analysis and wrote the first draft of the manuscript. Z.J., Y.L.R., X.J.G., W.M.S., Q.Q.D., K.F.Y., and B.C. extracted the data and contributed to the statistical analysis. J.Y.L. and B.Y. revised and discussed the final edition. Y.P.C. supervised the project and revised and discussed the final edition. All authors read and approved the final version of the manuscript.

## Funding

This study was supported by the National Key Research and Development Program of China (Grant No. 2022YFC2703101 to Y.P.C), the National Natural Science Fund of China (Grant No. 82371422 and 81971188 to Y.P.C.), the National Natural Science Fund of Sichuan (Grant No. 2022NSFSC0749 to B.C.), and the Science and Technology Bureau Fund of Sichuan Province (Grant No. 2023YFS0269 to Y.P.C).

## Ethics Statement

This research involves analyzing publicly available data, for which ethical approval and individual consent were obtained from all original studies.

## Conflicts of Interest

The authors declare no conflicts of interest.

## Supporting information




**Supplementary Materials**: brb371366‐sup‐0001‐SuppMat.doc

**Supplementary Materials**: brb371366‐sup‐0002‐SuppMat.xlsx

**Supplementary Materials**: brb371366‐sup‐0003‐SuppMat.doc

## Data Availability

The GWAS summary statistics supporting this research are available from the corresponding GWAS consortium. The main paper and Supporting Information present all data supporting our findings. The code or algorithm used to generate results in this study is available from the corresponding authors upon reasonable request.
