# Supplementary material for: Integrative Multi‐Omics Mendelian Randomization Highlights Causal Autophagy‐Related Genes for Amyotrophic Lateral Sclerosis
Source: Brain Behav. 2026 Mar 31;16(4):e71366. doi: 10.1002/brb3.71366 (PMC13112017; doi:10.1002/brb3.71366)
Supplement: Supplementary file 3 — Supplementary Materials: brb371366‐sup‐0003‐SuppMat.doc [file BRB3-16-e71366-s003.doc]

**Supplementary material**

**Contents**

**Supplementary methods**

**Supplementary tables**

**Supplementary Table 1.** Information and categories of 604 autophagy-related genes.

**Supplementary Table 2.** SMR and colocalization results of the association between exposures of autophagy-related genes and ALS risk.

**Supplementary Table 3.** Sensitivity analysis used the TwoSampleMR package on the association between exposures of autophagy-related genes and ALS risk.

**Supplementary Table 4.** Phenome-wide scan of the association between identified SNPs with other disease traits using PhenoScanner.

**Supplementary Table 5.** SMR results of the association between DNA methylation/RNA splicing and expression of autophagy-related genes.

**Supplementary Table 6**. Cell-type-specific MR analyses of identified causal autophagy-related genes.

**Supplementary Table 7.** Known ALS causative/risk genes from ALSoD.

**Supplementary Table 8.** Druggability of the causal autophagy-related genes for ALS.

**Supplementary figures**

**Supplementary Figure 1.** Leave-one-out sensitivity analyses of the SNPs represented the blood expression of *NME4* and ALS risk.

**Supplementary method references.**

**Supplementary methods**

### Summary-data-based Mendelian randomization analyses

Summary-data-based Mendelian randomization (SMR) employed a two-step least-squares method to estimate the effect size of an exposure on an outcome using a genetic variant as an instrument that exhibited a significant association with the exposure ^1^. In order to satisfy the assumptions of Mendelian randomization in our study, we selected SNPs with a *P*_SNP-autodys_ value less than 5 × 10^−8^ and included them (assumption 1). Additionally, we excluded all SNPs with a linkage disequilibrium (LD) r-squared value greater than 0.90 or less than 0.05 (assumptions 2 and 3). The causal associations were subsequently calculated as follows:

*β*_autodys-ALS_ = *β*_SNP-ALS_ / *β*_SNP-autodys_

The effect size of autophagy dysfunction on ALS, denoted as *β*_autodys-ALS_, is determined by the estimated effect size of a genetic variant (SNP) on autophagy dysfunction (*β*_SNP-autodys_) and the estimated effect size of the same genetic variant on ALS (*β*_SNP-ALS_).

To control the genome-wide type I error, we adjusted the SMR *P*-value with using false discovery rate (FDR) to account for multiple testing. We report SNP-gene combinations as significant with FDR *P*_SMR_ < 0.01. SNP-gene combinations with FDR *P*_SMR_ ≥ 0.01 but unadjusted *P*_SMR_ < 0.05 was considered suggestively significant.

In our study, the SMR approach incorporated the heterogeneity in dependent instruments (HEIDI) test to determine whether the observed association was primarily attributed to vertical pleiotropy or to linkage disequilibrium (LD) with the causal variant. The LD estimation was conducted using genomic data from individuals of European ancestry obtained from the 1000 Genomes Project Consortium as a reference dataset ^2^. Associations with a HEIDI test p-value (*P*_HEIDI_) of ≤ 0.01 were considered indicative of a potential influence of linkage rather than pleiotropy (where the same genetic variant independently influences both the outcome and the exposure), and thus, these associations were excluded from the subsequent analysis ^3^.

### Colocalization Analysis

Colocalization serves as a valuable approach for evaluating the existence of a shared causal variant within a specific genomic region affecting two distinct traits. The fundamental hypothesis underlying colocalization analysis is:

H0: neither trait has a genetic association in the region

H1: only trait 1 has a genetic association in the region

H2: only trait 2 has a genetic association in the region

H3: both traits are associated, but with different causal variants

H4: both traits are associated and share a single causal variant

The HEIDI test is a colocalization method that utilizes an external reference to estimate the LD between variants. To enhance the accuracy of our findings, we conducted an additional Bayesian test for colocalization, aiming to estimate the posterior probability of shared variants between two traits ^4^. For each leading SNP in the ALS GWAS database under investigation, we retrieved all SNPs located within a 100 kb region upstream and downstream of the leading SNPs for the purpose of colocalization analysis. This analysis involved estimating the posterior probability of H4 (PP.H4), utilizing default priors indicating that the probability of a shared causal genetic variant for trait 1 (P1) and trait 2 (P2) is 10^-4^, and the probability of a shared causal genetic variant across both traits is P12 = 5 × 10^-5^. While the threshold for PP.H4 can be adjusted to account for multiple hypothesis testing, a widely employed cut-off for indicating evidence of colocalization between the GWAS and QTL association is PP.H4 > 0.8.

**Supplementary Table 1.** Information and categories of 604 autophagy-related genes.

| **Gene symbol** | **Ensembl Gene ID** | **Synonyms** | **Chr** | **Gene start** | **Gene end** | **Biological Function** | **Description** | **Refs** | **Main categories** |
| --- | --- | --- | --- | --- | --- | --- | --- | --- | --- |
| *LAMTOR1* | ENSG00000149357 | C11orf59|PDRO|Ragulator1|p18|p27RF-Rho | 11 | 71796941 | 71814433 | mTOR regulators | late endosomal/lysosomal adaptor, MAPK and MTOR activator 1 | Trends Cell Biol. 2014 Jul;24(7):400-6. Regulation of mTORC1 by amino acids. Bar-Peled L, Sabatini DM. | mTOR and upstream pathways |
| *LAMTOR2* | ENSG00000116586 | ENDAP|HSPC003|MAPBPIP|MAPKSP1AP|ROBLD3|Ragulator2|p14 | 1 | 156024543 | 156028301 | mTOR regulators | late endosomal/lysosomal adaptor, MAPK and MTOR activator 2 | Trends Cell Biol. 2014 Jul;24(7):400-6. Regulation of mTORC1 by amino acids. Bar-Peled L, Sabatini DM. | mTOR and upstream pathways |
| *LAMTOR3* | ENSG00000109270 | MAP2K1IP1|MAPBP|MAPKSP1|MP1|PRO0633|Ragulator3 | 4 | 100799493 | 100815647 | mTOR regulators | late endosomal/lysosomal adaptor, MAPK and MTOR activator 3 | Trends Cell Biol. 2014 Jul;24(7):400-6. Regulation of mTORC1 by amino acids. Bar-Peled L, Sabatini DM. | mTOR and upstream pathways |
| *LAMTOR4* | ENSG00000188186 | C7orf59 | 7 | 99746530 | 99753567 | mTOR regulators | late endosomal/lysosomal adaptor, MAPK and MTOR activator 4 | Trends Cell Biol. 2014 Jul;24(7):400-6. Regulation of mTORC1 by amino acids. Bar-Peled L, Sabatini DM. | mTOR and upstream pathways |
| *LAMTOR5* | ENSG00000134248 | HBXIP|XIP | 1 | 110943871 | 110950564 | mTOR regulators | late endosomal/lysosomal adaptor, MAPK and MTOR activator 5 | Trends Cell Biol. 2014 Jul;24(7):400-6. Regulation of mTORC1 by amino acids. Bar-Peled L, Sabatini DM. | mTOR and upstream pathways |
| *FLCN* | ENSG00000154803 | BHD|DENND8B|FLCL | 17 | 17115526 | 17140502 | mTOR regulators | folliculin | Trends Cell Biol. 2014 Jul;24(7):400-6. Regulation of mTORC1 by amino acids. Bar-Peled L, Sabatini DM. | mTOR and upstream pathways |
| *FNIP1* | ENSG00000217128 | - | 5 | 130977407 | 131132710 | mTOR regulators | folliculin interacting protein 1 | Trends Cell Biol. 2014 Jul;24(7):400-6. Regulation of mTORC1 by amino acids. Bar-Peled L, Sabatini DM. | mTOR and upstream pathways |
| *FNIP2* | ENSG00000052795 | FNIPL|MAPO1 | 4 | 159690290 | 159829201 | mTOR regulators | Folliculin Interacting Protein 2 | Nat Rev Mol Cell Biol. 2020 Apr;21(4):183-203. mTOR at the nexus of nutrition, growth, ageing and disease. Liu GY, Sabatini DM5. | mTOR and upstream pathways |
| *MIOS* | ENSG00000164654 | MIO|Sea4|Yulink | 7 | 7606503 | 7648560 | mTOR regulators | missing oocyte, meiosis regulator, homolog (Drosophila) | Trends Cell Biol. 2014 Jul;24(7):400-6. Regulation of mTORC1 by amino acids. Bar-Peled L, Sabatini DM. | mTOR and upstream pathways |
| *SEH1L* | ENSG00000085415 | SEC13L|SEH1A|SEH1B|Seh1 | 18 | 12947132 | 12987535 | mTOR regulators | SEH1-like (S. cerevisiae) | Trends Cell Biol. 2014 Jul;24(7):400-6. Regulation of mTORC1 by amino acids. Bar-Peled L, Sabatini DM. | mTOR and upstream pathways |
| *WDR24* | ENSG00000127580 | C16orf21|JFP7 | 16 | 734622 | 740444 | mTOR regulators | WD repeat domain 24 | Trends Cell Biol. 2014 Jul;24(7):400-6. Regulation of mTORC1 by amino acids. Bar-Peled L, Sabatini DM. | mTOR and upstream pathways |
| *WDR59* | ENSG00000103091 | CDW12|FP977|p90-120 | 16 | 74907468 | 75034071 | mTOR regulators | WD repeat domain 59 | Trends Cell Biol. 2014 Jul;24(7):400-6. Regulation of mTORC1 by amino acids. Bar-Peled L, Sabatini DM. | mTOR and upstream pathways |
| *SEC13* | ENSG00000157020 | D3S1231E|SEC13L1|SEC13R|npp-20 | 3 | 10334815 | 10362862 | mTOR regulators | SEC13 homolog (S. cerevisiae) | Trends Cell Biol. 2014 Jul;24(7):400-6. Regulation of mTORC1 by amino acids. Bar-Peled L, Sabatini DM. | mTOR and upstream pathways |
| *RHEB* | ENSG00000106615 | RHEB2 | 7 | 151163098 | 151217206 | mTOR regulators | Ras homolog enriched in brain | Trends Cell Biol. 2014 Jul;24(7):400-6. Regulation of mTORC1 by amino acids. Bar-Peled L, Sabatini DM. | mTOR and upstream pathways |
| *NPC1* | ENSG00000141458 | NPC|POGZ|SLC65A1 | 18 | 21086148 | 21166862 | mTOR regulators | Niemann-Pick disease, type C1 | Science. 2017 Mar 24;355(6331):1306-1311. Lysosomal cholesterol activates mTORC1 via an SLC38A9-Niemann-Pick C1 signaling complex. Castellano BM, Thelen AM, Moldavski O, Feltes M, van der Welle RE, Mydock-McGrane L, Jiang X, van Eijkeren RJ, Davis OB, Louie SM, Perera RM, Covey DF, Nomura DK, Ory DS, Zoncu R. | mTOR and upstream pathways |
| *SLC38A9* | ENSG00000177058 | URLC11 | 5 | 54921673 | 55069022 | mTOR regulators | solute carrier family 38, member 9 | Science. 2017 Mar 24;355(6331):1306-1311. Lysosomal cholesterol activates mTORC1 via an SLC38A9-Niemann-Pick C1 signaling complex. Castellano BM, Thelen AM, Moldavski O, Feltes M, van der Welle RE, Mydock-McGrane L, Jiang X, van Eijkeren RJ, Davis OB, Louie SM, Perera RM, Covey DF, Nomura DK, Ory DS, Zoncu R. | mTOR and upstream pathways |
| *NPC2* | ENSG00000119655 | EDDM1|HE1 | 14 | 74942895 | 74960880 | mTOR regulators | Niemann-Pick disease, type C2 | Proc Natl Acad Sci U S A. 2010 Mar 9;107(10):4764-9. Cholesterol trafficking is required for mTOR activation in endothelial cells. Xu J, Dang Y, Ren YR, Liu JO. | mTOR and upstream pathways |
| *BTRC* | ENSG00000166167 | BETA-TRCP|FBW1A|FBXW1|FBXW1A|FWD1|bTrCP|bTrCP1|betaTrCP | 10 | 103113820 | 103317078 | mTOR regulators | beta-transducin repeat containing E3 ubiquitin protein ligase | Mol Cell. 2011 Oct 21;44(2):304-16. DEPTOR, an mTOR inhibitor, is a physiological substrate of SCF(βTrCP) E3 ubiquitin ligase and regulates survival and autophagy. Zhao Y, Xiong X, Sun Y. | mTOR and upstream pathways |
| *SESN2* | ENSG00000130766|ENSG00000285069 | HI95|SES2|SEST2 | 1 | 28586038 | 28609002 | mTOR regulators | sestrin 2 | Cell Death Differ. 2013 Apr;20(4):611-9. Sestrin2 integrates Akt and mTOR signaling to protect cells against energetic stress-induced death. Ben-Sahra I, Dirat B, Laurent K, Puissant A, Auberger P, Budanov A, Tanti JF, Bost F. | mTOR and upstream pathways |
| *SESN1* | ENSG00000080546 | PA26|SEST1 | 6 | 109307640 | 109416022 | mTOR regulators | sestrin 1 | Cell. 2008 Aug 8;134(3):451-60. p53 target genes sestrin1 and sestrin2 connect genotoxic stress and mTOR signaling. Budanov AV, Karin M. | mTOR and upstream pathways |
| *SESN3* | ENSG00000149212 | SEST3 | 11 | 94898704 | 94965705 | mTOR regulators | sestrin 3 | Dev Cell. 2010 Apr 20;18(4):592-604. FoxOs inhibit mTORC1 and activate Akt by inducing the expression of Sestrin3 and Rictor. Chen CC, Jeon SM, Bhaskar PT, Nogueira V, Sundararajan D, Tonic I, Park Y, Hay N. | mTOR and upstream pathways |
| *KIAA1524* | ENSG00000163507 | CIP2A | 3 | 108268716 | 108308491 | mTOR regulators | KIAA1524 | J Cell Biol. 2014 Mar 3;204(5):713-27. CIP2A oncoprotein controls cell growth and autophagy through mTORC1 activation. Puustinen P, Rytter A, Mortensen M, Kohonen P, Moreira JM, Jäättelä M. | mTOR and upstream pathways |
| *PPP2CA* | ENSG00000113575 | NEDLBA|PP2Ac|PP2CA|PP2Calpha|RP-C | 5 | 133530025 | 133561833 | mTOR regulators | protein phosphatase 2, catalytic subunit, alpha isozyme | Crit Rev Biochem Mol Biol. 2016 May-Jun;51(3):162-84. PP2A as a master regulator of the cell cycle. Wlodarchak N, Xing Y. | mTOR and upstream pathways |
| *RRAGA* | ENSG00000155876 | FIP-1|FIP1|RAGA | 9 | 19049372 | 19051019 | mTOR regulators | Ras-related GTP binding A | Nat Rev Mol Cell Biol. 2014 Mar;15(3):155-62. Making new contacts: the mTOR network in metabolism and signalling crosstalk. Shimobayashi M, Hall MN | mTOR and upstream pathways |
| *RRAGC* | ENSG00000116954 | GTR2|RAGC|TIB929 | 1 | 39303870 | 39325495 | mTOR regulators | Ras-related GTP binding C | Nat Rev Mol Cell Biol. 2014 Mar;15(3):155-62. Making new contacts: the mTOR network in metabolism and signalling crosstalk. Shimobayashi M, Hall MN | mTOR and upstream pathways |
| *RRAGD* | ENSG00000025039 | RAGD|bA11D8.2.1 | 6 | 90074355 | 90121989 | mTOR regulators | Ras-related GTP binding D | Nat Rev Mol Cell Biol. 2014 Mar;15(3):155-62. Making new contacts: the mTOR network in metabolism and signalling crosstalk. Shimobayashi M, Hall MN | mTOR and upstream pathways |
| *RRAGB* | ENSG00000083750 | RAGB|bA465E19.1 | X | 55744172 | 55785207 | mTOR regulators | Ras-related GTP binding B | Nat Rev Mol Cell Biol. 2014 Mar;15(3):155-62. Making new contacts: the mTOR network in metabolism and signalling crosstalk. Shimobayashi M, Hall MN | mTOR and upstream pathways |
| *TSC2* | ENSG00000103197 | LAM|PPP1R160|TSC4 | 16 | 2097466 | 2138716 | mTOR regulators | tuberous sclerosis 2 | Trends Cell Biol. 2014 Jul;24(7):400-6. Regulation of mTORC1 by amino acids. Bar-Peled L, Sabatini DM. | mTOR and upstream pathways |
| *TSC1* | ENSG00000165699 | LAM|TSC | 9 | 135766735 | 135820020 | mTOR regulators | tuberous sclerosis 1 | Trends Cell Biol. 2014 Jul;24(7):400-6. Regulation of mTORC1 by amino acids. Bar-Peled L, Sabatini DM. | mTOR and upstream pathways |
| *BMT2* | ENSG00000164603 | C7orf60|SAMTOR | 7 | 112459202 | 112579971 | mTOR regulators | Base Methyltransferase Of 25S RRNA 2 Homolog | Science. 2017 Nov 10;358(6364):813-818. SAMTOR is an S-adenosylmethionine sensor for the mTORC1 pathway. Gu X, Orozco JM, Saxton RA, Condon KJ, Liu GY, Krawczyk PA, Scaria SM, Harper JW, Gygi SP, Sabatini DM6. | mTOR and upstream pathways |
| *NPRL2* | ENSG00000114388 | FFEVF2|NPR2|NPR2L|TUSC4 | 3 | 50384761 | 50388522 | mTOR regulators | nitrogen permease regulator-like 2 (S. cerevisiae) | Nat Rev Mol Cell Biol. 2020 Apr;21(4):183-203. mTOR at the nexus of nutrition, growth, ageing and disease. Liu GY, Sabatini DM5. | mTOR and upstream pathways |
| *NPRL3* | ENSG00000103148 | C16orf35|CGTHBA|FFEVF3|HS-40|MARE|NPR3|RMD11 | 16 | 134273 | 188859 | mTOR regulators | nitrogen permease regulator-like 3 (S. cerevisiae) | Nat Rev Mol Cell Biol. 2020 Apr;21(4):183-203. mTOR at the nexus of nutrition, growth, ageing and disease. Liu GY, Sabatini DM5. | mTOR and upstream pathways |
| *DEPDC5* | ENSG00000100150 | DEP.5|FFEVF|FFEVF1 | 22 | 32149944 | 32303012 | mTOR regulators | DEP Domain Containing 5, GATOR1 Subcomplex Subunit | Nat Rev Mol Cell Biol. 2020 Apr;21(4):183-203. mTOR at the nexus of nutrition, growth, ageing and disease. Liu GY, Sabatini DM5. | mTOR and upstream pathways |
| *KPTN* | ENSG00000118162 | 2E4|MRT41 | 19 | 47978401 | 47987525 | mTOR regulators | Kaptin, Actin Binding Protein | Nat Rev Mol Cell Biol. 2020 Apr;21(4):183-203. mTOR at the nexus of nutrition, growth, ageing and disease. Liu GY, Sabatini DM5. | mTOR and upstream pathways |
| *ITFG2* | ENSG00000111203 | FGGAP1|MDS028 | 12 | 2921788 | 2968957 | mTOR regulators | Integrin Alpha FG-GAP Repeat Containing 2 | Nat Rev Mol Cell Biol. 2020 Apr;21(4):183-203. mTOR at the nexus of nutrition, growth, ageing and disease. Liu GY, Sabatini DM5. | mTOR and upstream pathways |
| *C12orf66* | ENSG00000174206 | - | 12 | 64580096 | 64616076 | mTOR regulators | Chromosome 12 Open Reading Frame 66 | Nat Rev Mol Cell Biol. 2020 Apr;21(4):183-203. mTOR at the nexus of nutrition, growth, ageing and disease. Liu GY, Sabatini DM5. | mTOR and upstream pathways |
| *SZT2* | ENSG00000198198 | C1orf84|EIEE18|KIAA0467|SZT2A|SZT2B | 1 | 43855553 | 43918321 | mTOR regulators | SZT2 Subunit Of KICSTOR Complex | Nat Rev Mol Cell Biol. 2020 Apr;21(4):183-203. mTOR at the nexus of nutrition, growth, ageing and disease. Liu GY, Sabatini DM5. | mTOR and upstream pathways |
| *CASTOR1* | ENSG00000239282 | GATSL3 | 22 | 30681106 | 30685616 | mTOR regulators | Cytosolic Arginine Sensor For MTORC1 Subunit 1 | Nat Rev Mol Cell Biol. 2020 Apr;21(4):183-203. mTOR at the nexus of nutrition, growth, ageing and disease. Liu GY, Sabatini DM5. | mTOR and upstream pathways |
| *CASTOR2* | ENSG00000274070 | GATSL1|GATSL2 | 7 | 74964705 | 75031528 | mTOR regulators | Cytosolic Arginine Sensor For MTORC1 Subunit 2 | Nat Rev Mol Cell Biol. 2020 Apr;21(4):183-203. mTOR at the nexus of nutrition, growth, ageing and disease. Liu GY, Sabatini DM5. | mTOR and upstream pathways |
| *LGALS8* | ENSG00000116977 | Gal-8|PCTA-1|PCTA1|Po66-CBP | 1 | 236681300 | 236716281 | mTOR regulators | Galectin 8 | Mol Cell. 2018 Apr 5;70(1):120-135.e8. Galectins Control mTOR in Response to Endomembrane Damage. Jia J, Abudu YP, Claude-Taupin A, Gu Y, Kumar S, Choi SW, Peters R, Mudd MH, Allers L, Salemi M, Phinney B, Johansen T, Deretic V. | mTOR and upstream pathways |
| *ATP6V0E1* | ENSG00000113732 | ATP6H|ATP6V0E|M9.2|Vma21|Vma21p | 5 | 172410760 | 172462448 | mTOR regulators | ATPase, H+ transporting, lysosomal 9kDa, V0 subunit e1 | Hum Mol Genet. 2011 Oct 1;20(19):3852-66. Characterization of the CLEAR network reveals an integrated control of cellular clearance pathways. Palmieri M | mTOR and upstream pathways |
| *ATP6V1D* | ENSG00000100554 | ATP6M|VATD|VMA8 | 14 | 67761088 | 67826982 | mTOR regulators | ATPase, H+ transporting, lysosomal 34kDa, V1 subunit D | Traffic. 2007 Dec;8(12):1676-86. Integral and associated lysosomal membrane proteins. Schröder B | mTOR and upstream pathways |
| *ATP6V1G3* | ENSG00000151418|ENSG00000263014 | ATP6G3|Vma10 | 1 | 198492352 | 198510075 | mTOR regulators | ATPase, H+ transporting, lysosomal 13kDa, V1 subunit G3 | Sci Rep. 2015; 5: 14827. Mapping the H+ (V)-ATPase interactome: identification of proteins involved in trafficking, folding, assembly and phosphorylationMaria Merkulova, Teodor G. Păunescu, Anie Azroyan, Vladimir Marshansky, Sylvie Breton, and Dennis Browna. | mTOR and upstream pathways |
| *ATP6V1C2* | ENSG00000143882 | ATP6C2|VMA5 | 2 | 10861775 | 10925236 | mTOR regulators | ATPase, H+ transporting, lysosomal 42kDa, V1 subunit C2 | 1) Hum Mol Genet. 2011 Oct 1;20(19):3852-66. Characterization of the CLEAR network reveals an integrated control of cellular clearance pathways. Palmieri M; 2) Traffic. 2007 Dec;8(12):1676-86. Integral and associated lysosomal membrane proteins. Schröder B. | mTOR and upstream pathways |
| *ATP6V1B2* | ENSG00000147416 | ATP6B1B2|ATP6B2|DOOD|HO57|VATB|VPP3|Vma2|ZLS2 | 8 | 20054878 | 20084330 | mTOR regulators | ATPase, H+ transporting, lysosomal 56/58kDa, V1 subunit B2 | 1) Traffic. 2007 Dec;8(12):1676-86. Integral and associated lysosomal membrane proteins. Schröder B; 2) Cell Res. 2014 Nov;24(11):1370-3. De novo mutation in ATP6V1B2 impairs lysosome acidification and causes dominant deafness-onychodystrophy syndrome. Yuan Y | mTOR and upstream pathways |
| *ATP6V0A1* | ENSG00000033627 | ATP6N1|ATP6N1A|Stv1|VPP1|Vph1|a1 | 17 | 40610862 | 40674629 | mTOR regulators | ATPase, H+ transporting, lysosomal V0 subunit a1 | Sci Rep. 2015; 5: 14827. Mapping the H+ (V)-ATPase interactome: identification of proteins involved in trafficking, folding, assembly and phosphorylationMaria Merkulova, Teodor G. Păunescu, Anie Azroyan, Vladimir Marshansky, Sylvie Breton, and Dennis Browna. | mTOR and upstream pathways |
| *ATP6V1B1* | ENSG00000116039 | ATP6B1|RTA1B|VATB|VMA2|VPP3 | 2 | 71163012 | 71192536 | mTOR regulators | ATPase, H+ transporting, lysosomal 56/58kDa, V1 subunit B1 | 1) Hum Mol Genet. 2011 Oct 1;20(19):3852-66. Characterization of the CLEAR network reveals an integrated control of cellular clearance pathways. Palmieri M; 2) Traffic. 2007 Dec;8(12):1676-86. Integral and associated lysosomal membrane proteins. Schröder B. | mTOR and upstream pathways |
| *ATP6V1G2* | ENSG00000206445|ENSG00000213760|ENSG00000226850|ENSG00000227587|ENSG00000230900|ENSG00000234668|ENSG00000234920 | ATP6G|ATP6G2|NG38|VMA10 | 6 | 31512239 | 31516204 | mTOR regulators | ATPase, H+ transporting, lysosomal 13kDa, V1 subunit G2 | Theranostics. 2018; 8(19): 5379–5399. V-ATPases and osteoclasts: ambiguous future of V-ATPases inhibitors in osteoporosisDuan X, Yang S, Zhang L, Yang T. | mTOR and upstream pathways |
| *ATP6V1E2* | ENSG00000250565 | ATP6E1|ATP6EL2|ATP6V1EL2|VMA4 | 2 | 46717889 | 46769696 | mTOR regulators | ATPase, H+ transporting, lysosomal 31kDa, V1 subunit E2 | Gene. 2002 May 1;289(1-2):7-12. A human gene, ATP6E1, encoding a testis-specific isoform of H(+)-ATPase subunit E. Imai-Senga Y, Sun-Wada GH, Wada Y, Futai M. | mTOR and upstream pathways |
| *ATP6V0A4* | ENSG00000105929 | A4|ATP6N1B|ATP6N2|RDRTA2|RTA1C|RTADR|STV1|VPH1|VPP2 | 7 | 138391040 | 138484305 | mTOR regulators | ATPase, H+ transporting, lysosomal V0 subunit a4 | 1) Autophagy. 2015;11(5):756-68. The integral membrane protein ITM2A, a transcriptional target of PKA-CREB, regulates autophagic flux via interaction with the vacuolar ATPase. Namkoong SPalmieri M; 2) Traffic. 2007 Dec;8(12):1676-86. Integral and associated lysosomal membrane proteins. Schröder B. | mTOR and upstream pathways |
| *ATP6V1E1* | ENSG00000131100 | ARCL2C|ATP6E|ATP6E2|ATP6V1E|P31|Vma4 | 22 | 18074902 | 18111584 | mTOR regulators | ATPase, H+ transporting, lysosomal 31kDa, V1 subunit E1 | 1) Hum Mol Genet. 2011 Oct 1;20(19):3852-66. Characterization of the CLEAR network reveals an integrated control of cellular clearance pathways. Palmieri M; 2) Traffic. 2007 Dec;8(12):1676-86. Integral and associated lysosomal membrane proteins. Schröder B. | mTOR and upstream pathways |
| *ATP6V0B* | ENSG00000117410 | ATP6F|HATPL|VMA16 | 1 | 44440159 | 44443967 | mTOR regulators | ATPase, H+ transporting, lysosomal 21kDa, V0 subunit b | Hum Mol Genet. 2011 Oct 1;20(19):3852-66. Characterization of the CLEAR network reveals an integrated control of cellular clearance pathways. Palmieri M | mTOR and upstream pathways |
| *TCIRG1* | ENSG00000110719 | ATP6N1C|ATP6V0A3|Atp6i|OC-116kDa|OC116|OPTB1|Stv1|TIRC7|Vph1|a3 | 11 | 67806483 | 67818362 | mTOR regulators | T-cell, immune regulator 1, ATPase, H+ transporting, lysosomal V0 subunit A3 | "Sci Rep. 2015; 5: 14827. Mapping the H+ (V)-ATPase interactome: identification of proteins involved in trafficking, folding, assembly and phosphorylationMaria Merkulova, Teodor G. Păunescu, Anie Azroyan, Vladimir Marshansky, Sylvie Breton, and Dennis Browna." | mTOR and upstream pathways |
| *ATP6AP2* | ENSG00000182220 | APT6M8-9|ATP6IP2|ATP6M8-9|CDG2R|ELDF10|HT028|M8-9|MRXE|MRXSH|MSTP009|PRR|RENR|XMRE|XPDS | X | 40440146 | 40465889 | mTOR regulators | ATPase, H+ transporting, lysosomal accessory protein 2 | EMBO J. 2011 Jul 29;30(16):3242-58. Regulation of TFEB and V-ATPases by mTORC1. Peña-Llopis S | mTOR and upstream pathways |
| *ATP6V1A* | ENSG00000114573 | ARCL2D|ATP6A1|ATP6V1A1|HO68|IECEE3|VA68|VPP2|Vma1 | 3 | 113465866 | 113530903 | mTOR regulators | ATPase, H+ transporting, lysosomal 70kDa, V1 subunit A | 1) Hum Mol Genet. 2011 Oct 1;20(19):3852-66. Characterization of the CLEAR network reveals an integrated control of cellular clearance pathways. Palmieri M; 2) Traffic. 2007 Dec;8(12):1676-86. Integral and associated lysosomal membrane proteins. Schröder B. | mTOR and upstream pathways |
| *ATP6AP1* | ENSG00000071553 | 16A|ATP6IP1|ATP6S1|Ac45|CF2|VATPS1|XAP-3|XAP3 | X | 153656978 | 153664862 | mTOR regulators | ATPase, H+ transporting, lysosomal accessory protein 1 | Curr Protein Pept Sci. 2012 Mar;13(2):124-33. Novel insights into V-ATPase functioning: distinct roles for its accessory subunits ATP6AP1/Ac45 and ATP6AP2/(pro) renin receptor. Jansen EJ, Martens GJ. | mTOR and upstream pathways |
| *ATP6V1G1* | ENSG00000136888 | ATP6G|ATP6G1|ATP6GL|ATP6J|Vma10 | 9 | 117350026 | 117360653 | mTOR regulators | ATPase, H+ transporting, lysosomal 13kDa, V1 subunit G1 | 1) Hum Mol Genet. 2011 Oct 1;20(19):3852-66. Characterization of the CLEAR network reveals an integrated control of cellular clearance pathways. Palmieri M; 2) Traffic. 2007 Dec;8(12):1676-86. Integral and associated lysosomal membrane proteins. Schröder B. | mTOR and upstream pathways |
| *ATP6V0E2* | ENSG00000171130 | ATP6V0E2L|C7orf32 | 7 | 149570057 | 149577784 | mTOR regulators | ATPase, H+ transporting V0 subunit e2 | Gene. 2007 May 15;393(1-2):94-100. Molecular cloning and characterization of a novel form of the human vacuolar H+-ATPase e-subunit: an essential proton pump component. Blake-Palmer KG, Su Y, Smith AN, Karet FE. | mTOR and upstream pathways |
| *ATP6V0C* | ENSG00000185883 | ATP6C|ATP6L|ATPL|VATL|VPPC|Vma3 | 16 | 2563871 | 2570219 | mTOR regulators | ATPase, H+ transporting, lysosomal 16kDa, V0 subunit c | 1)PLoS One. 2014 Apr 2;9(4):e93257. ATP6V0C knockdown in neuroblastoma cells alters autophagy-lysosome pathway function and metabolism of proteins that accumulate in neurodegenerative disease. Mangieri LR; 2) Traffic. 2007 Dec;8(12):1676-86. Integral and associated lysosomal membrane proteins. Schröder B. | mTOR and upstream pathways |
| *ATP6AP1L* | ENSG00000205464 | - | 5 | 81575281 | 81682796 | mTOR regulators | ATPase, H+ transporting, lysosomal accessory protein 1-like | Nat Commun; 2016 May 27;7:11600. ATP6AP1 Deficiency Causes an Immunodeficiency With Hepatopathy, Cognitive Impairment and Abnormal Protein GlycosylationEric J R Jansen, Sharita Timal, Margret Ryan, Angel Ashikov, Monique van Scherpenzeel, Laurie A Graham, Hanna Mandel, Alexander Hoischen, Theodore C Iancu, Kimiyo Raymond, Gerry Steenbergen, Christian Gilissen, Karin Huijben, Nick H M van Bakel, Yusuke Maeda, Richard J Rodenburg, Maciej Adamowicz, Ellen Crushell, Hans Koenen, Darius Adams, Julia Vodopiutz, Susanne Greber-Platzer, Thomas Müller, Gregor Dueckers, Eva Morava, Jolanta Sykut-Cegielska, Gerard J M Martens, Ron A Wevers, Tim Niehues, Martijn A Huynen, Joris A Veltman, Tom H Stevens, Dirk J Lefeber. | mTOR and upstream pathways |
| *ATP6V1H* | ENSG00000047249 | CGI-11|MSTP042|NBP1|SFD|SFDalpha|SFDbeta|VMA13 | 8 | 54628117 | 54756118 | mTOR regulators | ATPase H+ Transporting V1 Subunit H | 1) Hum Mol Genet. 2011 Oct 1;20(19):3852-66. Characterization of the CLEAR network reveals an integrated control of cellular clearance pathways. Palmieri M; 2) Traffic. 2007 Dec;8(12):1676-86. Integral and associated lysosomal membrane proteins. Schröder B. | mTOR and upstream pathways |
| *ATP6V0A2* | ENSG00000185344 | A2|ARCL|ARCL2A|ATP6A2|ATP6N1D|J6B7|RTF|STV1|TJ6|TJ6M|TJ6S|VPH1|WSS | 12 | 124196865 | 124246302 | mTOR regulators | ATPase, H+ transporting, lysosomal V0 subunit a2 | 1) Hum Mol Genet. 2011 Oct 1;20(19):3852-66. Characterization of the CLEAR network reveals an integrated control of cellular clearance pathways. Palmieri M; 2) Traffic. 2007 Dec;8(12):1676-86. Integral and associated lysosomal membrane proteins. Schröder B. | mTOR and upstream pathways |
| *ATP6V1C1* | ENSG00000155097 | ATP6C|ATP6D|VATC|Vma5 | 8 | 104033291 | 104085279 | mTOR regulators | ATPase, H+ transporting, lysosomal 42kDa, V1 subunit C1 | 1) Hum Mol Genet. 2011 Oct 1;20(19):3852-66. Characterization of the CLEAR network reveals an integrated control of cellular clearance pathways. Palmieri M; 2) Traffic. 2007 Dec;8(12):1676-86. Integral and associated lysosomal membrane proteins. Schröder B. | mTOR and upstream pathways |
| *ATP6V1F* | ENSG00000128524 | ATP6S14|VATF|Vma7 | 7 | 128502880 | 128505898 | mTOR regulators | ATPase, H+ transporting, lysosomal 14kDa, V1 subunit F | Theranostics. 2018; 8(19): 5379–5399. V-ATPases and osteoclasts: ambiguous future of V-ATPases inhibitors in osteoporosisXiaohong Duan, Shaoqing Yang, Lei Zhang, and Tielin Yang | mTOR and upstream pathways |
| *ATP6V0D1* | ENSG00000159720 | ATP6D|ATP6DV|P39|VATX|VMA6|VPATPD | 16 | 67471917 | 67515140 | mTOR regulators | ATPase, H+ transporting, lysosomal 38kDa, V0 subunit d1 | 1) Hum Mol Genet. 2011 Oct 1;20(19):3852-66. Characterization of the CLEAR network reveals an integrated control of cellular clearance pathways. Palmieri M; 2) Traffic. 2007 Dec;8(12):1676-86. Integral and associated lysosomal membrane proteins. Schröder B. | mTOR and upstream pathways |
| *MTOR* | ENSG00000198793 | FRAP|FRAP1|FRAP2|RAFT1|RAPT1|SKS | 1 | 11166592 | 11322564 | mTOR complexes 1 and 2 | mechanistic target of rapamycin (serine/threonine kinase) | Nat Rev Mol Cell Biol. 2020 Jan 14. mTOR at the nexus of nutrition, growth, ageing and disease. Liu GY, Sabatini DM. | mTOR and upstream pathways |
| *DEPTOR* | ENSG00000155792 | DEP.6|DEPDC6 | 8 | 120885957 | 121063152 | mTOR complexes 1 and 2 | DEP domain containing MTOR-interacting protein | Nat Rev Mol Cell Biol. 2020 Jan 14. mTOR at the nexus of nutrition, growth, ageing and disease. Liu GY, Sabatini DM. | mTOR and upstream pathways |
| *RPTOR* | ENSG00000141564 | KOG1|Mip1 | 17 | 78518619 | 78940171 | mTOR complexes 1 and 2 | regulatory associated protein of MTOR, complex 1 | Nat Rev Mol Cell Biol. 2020 Jan 14. mTOR at the nexus of nutrition, growth, ageing and disease. Liu GY, Sabatini DM. | mTOR and upstream pathways |
| *AKT1S1* | ENSG00000204673 | Lobe|PRAS40 | 19 | 50372295 | 50381716 | mTOR complexes 1 and 2 | AKT1 substrate 1 (proline-rich) | Nat Rev Mol Cell Biol. 2020 Jan 14. mTOR at the nexus of nutrition, growth, ageing and disease. Liu GY, Sabatini DM. | mTOR and upstream pathways |
| *MLST8* | ENSG00000167965 | GBL|GbetaL|LST8|POP3|WAT1 | 16 | 2254249 | 2259417 | mTOR complexes 1 and 2 | MTOR associated protein, LST8 homolog (S. cerevisiae) | Nat Rev Mol Cell Biol. 2020 Jan 14. mTOR at the nexus of nutrition, growth, ageing and disease. Liu GY, Sabatini DM. | mTOR and upstream pathways |
| *RICTOR* | ENSG00000164327 | AVO3|PIA|hAVO3 | 5 | 38938021 | 39074510 | mTOR complexes 1 and 2 | RPTOR independent companion of MTOR, complex 2 | Nat Rev Mol Cell Biol. 2020 Jan 14. mTOR at the nexus of nutrition, growth, ageing and disease. Liu GY, Sabatini DM. | mTOR and upstream pathways |
| *PRR5* | ENSG00000186654 | FLJ20185k|PP610|PROTOR-1|PROTOR1 | 22 | 45064593 | 45133561 | mTOR complexes 1 and 2 | proline rich 5 (renal) | Nat Rev Mol Cell Biol. 2020 Jan 14. mTOR at the nexus of nutrition, growth, ageing and disease. Liu GY, Sabatini DM. | mTOR and upstream pathways |
| *MAPKAP1* | ENSG00000119487 | JC310|MIP1|SIN1|SIN1b|SIN1g | 9 | 128199672 | 128469513 | mTOR complexes 1 and 2 | mitogen-activated protein kinase associated protein 1 | Nat Rev Mol Cell Biol. 2020 Jan 14. mTOR at the nexus of nutrition, growth, ageing and disease. Liu GY, Sabatini DM. | mTOR and upstream pathways |
| *TELO2* | ENSG00000100726 | CLK2|TEL2|YHFS | 16 | 1543345 | 1560458 | mTOR complexes 1 and 2 | TEL2, telomere maintenance 2, homolog (S. cerevisiae) | J Cell Biol. 2013 Nov 25;203(4):563-74. Where is mTOR and what is it doing there?Betz C, Hall MN. | mTOR and upstream pathways |
| *TTI1* | ENSG00000101407 | KIAA0406|smg-10 | 20 | 36611409 | 36661870 | mTOR complexes 1 and 2 | TELO2 interacting protein 1 | J Cell Biol. 2013 Nov 25;203(4):563-74. Where is mTOR and what is it doing there?Betz C, Hall MN. | mTOR and upstream pathways |
| *TTI2* | ENSG00000129696 | C8orf41|MRT39 | 8 | 33330904 | 33371119 | mTOR complexes 1 and 2 | TELO2 interacting protein 2 | J Cell Biol. 2013 Nov 25;203(4):563-74. Where is mTOR and what is it doing there?Betz C, Hall MN. | mTOR and upstream pathways |
| *PRR5L* | ENSG00000135362 | PROTOR2 | 11 | 36317838 | 36486754 | mTOR complexes 1 and 2 | Proline Rich 5 Like | Cell. 2012 Apr 13;149(2):274-93. mTOR signaling in growth control and disease. Laplante M, Sabatini DM. | mTOR and upstream pathways |
| *PRKAA1* | ENSG00000132356 | AMPK|AMPKa1 | 5 | 40759481 | 40798476 | AMPK complex | protein kinase, AMP-activated, alpha 1 catalytic subunit | Cardiovasc Pharm Open Access. 2015 Aug;4(3):154. AMP-Activated Protein Kinase Signalling in Cancer and Cardiac Hypertrophy. Lipovka Y, Konhilas JP. | mTOR and upstream pathways |
| *PRKAA2* | ENSG00000162409 | AMPK|AMPK2|AMPKa2|PRKAA | 1 | 57110995 | 57181008 | AMPK complex | protein kinase, AMP-activated, alpha 2 catalytic subunit | Cardiovasc Pharm Open Access. 2015 Aug;4(3):154. AMP-Activated Protein Kinase Signalling in Cancer and Cardiac Hypertrophy. Lipovka Y, Konhilas JP. | mTOR and upstream pathways |
| *PRKAG1* | ENSG00000181929 | AMPKG | 12 | 49396057 | 49412980 | AMPK complex | protein kinase, AMP-activated, gamma 1 non-catalytic subunit | Cardiovasc Pharm Open Access. 2015 Aug;4(3):154. AMP-Activated Protein Kinase Signalling in Cancer and Cardiac Hypertrophy. Lipovka Y, Konhilas JP. | mTOR and upstream pathways |
| *PRKAG2* | ENSG00000106617 | AAKG|AAKG2|CMH6|H91620p|WPWS | 7 | 151253197 | 151574210 | AMPK complex | protein kinase, AMP-activated, gamma 2 non-catalytic subunit | Cardiovasc Pharm Open Access. 2015 Aug;4(3):154. AMP-Activated Protein Kinase Signalling in Cancer and Cardiac Hypertrophy. Lipovka Y, Konhilas JP. | mTOR and upstream pathways |
| *PRKAG3* | ENSG00000115592 | AMPKG3 | 2 | 219687106 | 219696809 | AMPK complex | protein kinase, AMP-activated, gamma 3 non-catalytic subunit | Cardiovasc Pharm Open Access. 2015 Aug;4(3):154. AMP-Activated Protein Kinase Signalling in Cancer and Cardiac Hypertrophy. Lipovka Y, Konhilas JP. | mTOR and upstream pathways |
| *PRKAB1* | ENSG00000111725 | AMPK|HAMPKb | 12 | 120105558 | 120119435 | AMPK complex | protein kinase, AMP-activated, beta 1 non-catalytic subunit | Cardiovasc Pharm Open Access. 2015 Aug;4(3):154. AMP-Activated Protein Kinase Signalling in Cancer and Cardiac Hypertrophy. Lipovka Y, Konhilas JP. | mTOR and upstream pathways |
| *PRKAB2* | ENSG00000131791 | - | 1 | 146626685 | 146644129 | AMPK complex | protein kinase, AMP-activated, beta 2 non-catalytic subunit | Cardiovasc Pharm Open Access. 2015 Aug;4(3):154. AMP-Activated Protein Kinase Signalling in Cancer and Cardiac Hypertrophy. Lipovka Y, Konhilas JP. | mTOR and upstream pathways |
| *STK11* | ENSG00000118046 | LKB1|PJS|hLKB1 | 19 | 1189406 | 1228428 | AMPK activator | serine/threonine kinase 11 | Nat Rev Drug Discov. 2011 Oct 31;10(11):868-80. Rapamycin passes the torch: a new generation of mTOR inhibitors. Benjamin D, Colombi M, Moroni C, Hall MN. | mTOR and upstream pathways |
| *STRADA* | ENSG00000266173 | LYK5|NY-BR-96|PMSE|STRAD|STRAD alpha|Stlk | 17 | 61780192 | 61819330 | AMPK activator | STE20-related kinase adaptor alpha | Cardiovasc Pharm Open Access. 2015 Aug;4(3):154. AMP-Activated Protein Kinase Signalling in Cancer and Cardiac Hypertrophy. Lipovka Y, Konhilas JP. | mTOR and upstream pathways |
| *STRADB* | ENSG00000082146 | ALS2CR2|CALS-21|ILPIP|ILPIPA|PAPK|PRO1038 | 2 | 202252581 | 202345569 | AMPK activator | STE20-related kinase adaptor beta | Cardiovasc Pharm Open Access. 2015 Aug;4(3):154. AMP-Activated Protein Kinase Signalling in Cancer and Cardiac Hypertrophy. Lipovka Y, Konhilas JP. | mTOR and upstream pathways |
| *CAB39* | ENSG00000135932 | CGI-66|MO25 | 2 | 231577560 | 231685790 | AMPK activator | calcium binding protein 39 | Cardiovasc Pharm Open Access. 2015 Aug;4(3):154. AMP-Activated Protein Kinase Signalling in Cancer and Cardiac Hypertrophy. Lipovka Y, Konhilas JP. | mTOR and upstream pathways |
| *CAB39L* | ENSG00000102547 | MO25-BETA|MO2L|bA103J18.3 | 13 | 49882786 | 50018262 | AMPK activator | calcium binding protein 39-like | Cardiovasc Pharm Open Access. 2015 Aug;4(3):154. AMP-Activated Protein Kinase Signalling in Cancer and Cardiac Hypertrophy. Lipovka Y, Konhilas JP. | mTOR and upstream pathways |
| *CAMKK2* | ENSG00000110931 | CAMKK|CAMKKB | 12 | 121675497 | 121736111 | AMPK activator | calcium/calmodulin-dependent protein kinase kinase 2, beta | Cardiovasc Pharm Open Access. 2015 Aug;4(3):154. AMP-Activated Protein Kinase Signalling in Cancer and Cardiac Hypertrophy. Lipovka Y, Konhilas JP. | mTOR and upstream pathways |
| *PIK3CA* | ENSG00000121879 | CLAPO|CLOVE|CWS5|MCAP|MCM|MCMTC|PI3K|PI3K-alpha|p110-alpha | 3 | 178865902 | 178957881 | PI3K complex-class 1 | phosphatidylinositol-4,5-bisphosphate 3-kinase, catalytic subunit alpha | J Cell Sci. 2014 Mar 1;127(Pt 5):923-8. Classes of phosphoinositide 3-kinases at a glance. Jean S, Kiger AA. | mTOR and upstream pathways |
| *PIK3CB* | ENSG00000051382 | P110BETA|PI3K|PI3KBETA|PIK3C1 | 3 | 138372860 | 138553780 | PI3K complex-class 1 | phosphatidylinositol-4,5-bisphosphate 3-kinase, catalytic subunit beta | J Cell Sci. 2014 Mar 1;127(Pt 5):923-8. Classes of phosphoinositide 3-kinases at a glance. Jean S, Kiger AA. | mTOR and upstream pathways |
| *PIK3CD* | ENSG00000171608 | APDS|IMD14|P110DELTA|PI3K|p110D | 1 | 9711790 | 9789172 | PI3K complex-class 1 | phosphatidylinositol-4,5-bisphosphate 3-kinase, catalytic subunit delta | J Cell Sci. 2014 Mar 1;127(Pt 5):923-8. Classes of phosphoinositide 3-kinases at a glance. Jean S, Kiger AA. | mTOR and upstream pathways |
| *PIK3CG* | ENSG00000105851 | PI3CG|PI3K|PI3Kgamma|PIK3|p110gamma|p120-PI3K | 7 | 106505723 | 106547590 | PI3K complex-class 1 | phosphatidylinositol-4,5-bisphosphate 3-kinase, catalytic subunit gamma | J Cell Sci. 2014 Mar 1;127(Pt 5):923-8. Classes of phosphoinositide 3-kinases at a glance. Jean S, Kiger AA. | mTOR and upstream pathways |
| *PIK3R1* | ENSG00000145675 | AGM7|GRB1|IMD36|p85|p85-ALPHA | 5 | 67511548 | 67597649 | PI3K complex-class 1 | phosphoinositide-3-kinase, regulatory subunit 1 (alpha) | J Cell Sci. 2014 Mar 1;127(Pt 5):923-8. Classes of phosphoinositide 3-kinases at a glance. Jean S, Kiger AA. | mTOR and upstream pathways |
| *PIK3R2* | ENSG00000105647 | MPPH|MPPH1|P85B|p85|p85-BETA | 19 | 18263928 | 18281350 | PI3K complex-class 1 | phosphoinositide-3-kinase, regulatory subunit 2 (beta) | J Cell Sci. 2014 Mar 1;127(Pt 5):923-8. Classes of phosphoinositide 3-kinases at a glance. Jean S, Kiger AA. | mTOR and upstream pathways |
| *PIK3R3* | ENSG00000117461 | p55|p55-GAMMA|p55PIK | 1 | 46505812 | 46642160 | PI3K complex-class 1 | phosphoinositide-3-kinase, regulatory subunit 3 (gamma) | J Cell Sci. 2014 Mar 1;127(Pt 5):923-8. Classes of phosphoinositide 3-kinases at a glance. Jean S, Kiger AA. | mTOR and upstream pathways |
| *PIK3R4* | ENSG00000196455 | VPS15|p150 | 3 | 130397779 | 130465673 | PI3K complex-class 1 | phosphoinositide-3-kinase, regulatory subunit 4 | J Cell Sci. 2014 Mar 1;127(Pt 5):923-8. Classes of phosphoinositide 3-kinases at a glance. Jean S, Kiger AA. | mTOR and upstream pathways |
| *PIK3R5* | ENSG00000141506 | F730038I15Rik|FOAP-2|P101-PI3K|p101 | 17 | 8782233 | 8869029 | PI3K complex-class 1 | phosphoinositide-3-kinase, regulatory subunit 5 | J Cell Sci. 2014 Mar 1;127(Pt 5):923-8. Classes of phosphoinositide 3-kinases at a glance. Jean S, Kiger AA. | mTOR and upstream pathways |
| *PIK3R6* | ENSG00000276231 | C17orf38|HsT41028|p84 PIKAP|p87(PIKAP)|p87PIKAP | 17 | 8706041 | 8770994 | PI3K complex-class 1 | phosphoinositide-3-kinase, regulatory subunit 6 | J Cell Sci. 2014 Mar 1;127(Pt 5):923-8. Classes of phosphoinositide 3-kinases at a glance. Jean S, Kiger AA. | mTOR and upstream pathways |
| *PIK3C2A* | ENSG00000011405 | CPK|OCSKD|PI3-K-C2(ALPHA)|PI3-K-C2A|PI3K-C2-alpha|PI3K-C2alpha | 11 | 17099277 | 17229530 | PI3K complex-class 2 | phosphatidylinositol-4-phosphate 3-kinase, catalytic subunit type 2 alpha | J Cell Sci. 2014 Mar 1;127(Pt 5):923-8. Classes of phosphoinositide 3-kinases at a glance. Jean S, Kiger AA. | mTOR and upstream pathways |
| *PIK3C2B* | ENSG00000133056 | C2-PI3K | 1 | 204391756 | 204463852 | PI3K complex-class 2 | phosphatidylinositol-4-phosphate 3-kinase, catalytic subunit type 2 beta | J Cell Sci. 2014 Mar 1;127(Pt 5):923-8. Classes of phosphoinositide 3-kinases at a glance. Jean S, Kiger AA. | mTOR and upstream pathways |
| *PIK3C2G* | ENSG00000139144 | PI3K-C2-gamma|PI3K-C2GAMMA | 12 | 18400548 | 18801348 | PI3K complex-class 2 | phosphatidylinositol-4-phosphate 3-kinase, catalytic subunit type 2 gamma | J Cell Sci. 2014 Mar 1;127(Pt 5):923-8. Classes of phosphoinositide 3-kinases at a glance. Jean S, Kiger AA. | mTOR and upstream pathways |
| *RPS6KB1* | ENSG00000108443 | PS6K|S6K|S6K-beta-1|S6K1|STK14A|p70 S6KA|p70(S6K)-alpha|p70-S6K|p70-alpha | 17 | 57970447 | 58027925 | mTORC1 substrates | ribosomal protein S6 kinase, 70kDa, polypeptide 1 | Cell. 2012 Apr 13;149(2):274-93. mTOR signaling in growth control and disease. Laplante M, Sabatini DM. | mTOR and upstream pathways |
| *RPS6KA1* | ENSG00000117676|ENSG00000281877 | HU-1|MAPKAPK1|MAPKAPK1A|RSK|RSK1|p90Rsk | 1 | 26856252 | 26901521 | mTORC1 substrates | ribosomal protein S6 kinase, 90kDa, polypeptide 1 | Nat Rev Mol Cell Biol. 2014 Mar;15(3):155-62. Making new contacts: the mTOR network in metabolism and signalling crosstalk. Shimobayashi M, Hall MN | mTOR and upstream pathways |
| *DAP* | ENSG00000112977 | - | 5 | 10679342 | 10761384 | mTORC1 substrates | death-associated protein | Curr Biol. 2010 Jun 22;20(12):1093-8. DAP1, a novel substrate of mTOR, negatively regulates autophagy. Koren I, Reem E, Kimchi A. | mTOR and upstream pathways |
| *EEF2K* | ENSG00000103319|ENSG00000284161 | CaMKIII|HSU93850|eEF-2K | 16 | 22217603 | 22298554 | mTORC1 substrates | eukaryotic elongation factor-2 kinase | FEBS J. 2005 Aug;272(16):4211-20. Levels of mTOR and its downstream targets 4E-BP1, eEF2, and eEF2 kinase in relationships with tau in Alzheimer's disease brain. Li X, Alafuzoff I, Soininen H, Winblad B, Pei JJ. | mTOR and upstream pathways |
| *EIF4EBP1* | ENSG00000187840 | 4E-BP1|4EBP1|BP-1|PHAS-I | 8 | 37887859 | 37917883 | mTORC1 substrates | eukaryotic translation initiation factor 4E binding protein 1 | Nat Rev Drug Discov. 2011 Oct 31;10(11):868-80. Rapamycin passes the torch: a new generation of mTOR inhibitors. Benjamin D, Colombi M, Moroni C, Hall MN. | mTOR and upstream pathways |
| *LARP1* | ENSG00000155506 | LARP|Lar1|Lhp1 | 5 | 154092462 | 154197167 | mTORC1 substrates | La Ribonucleoprotein 1, Translational Regulator | BiorxivLARP1 is a major phosphorylation substrate of mTORC1Fonseca D., Jia J. J., Hollensen A. K., Pointet R., …, Yu Y., Damgaard C. K., Berman A. J., Alain T. | mTOR and upstream pathways |
| *ESR1* | ENSG00000091831 | ER|ESR|ESRA|ESTRR|Era|NR3A1 | 6 | 151977826 | 152450754 | mTORC1 substrates | estrogen receptor 1 | Oncogene. 2016 Jul 7;35(27):3535-43. mTORC1 directly phosphorylates and activates ERα upon estrogen stimulation. Alayev A, Salamon RS, Berger SM, Schwartz NS, Cuesta R, Snyder RB, Holz MK. | mTOR and upstream pathways |
| *MAF1* | ENSG00000179632 | - | 8 | 145159402 | 145162514 | mTORC1 substrates | MAF1 homolog (S. cerevisiae) | Cell. 2012 Apr 13;149(2):274-93. mTOR signaling in growth control and disease. Laplante M, Sabatini DM. | mTOR and upstream pathways |
| *ULK1* | ENSG00000177169 | ATG1|ATG1A|UNC51|Unc51.1|hATG1 | 12 | 132379196 | 132407712 | mTORC1 substrates | unc-51 like autophagy activating kinase 1 | Cell Res. 2014 Jan;24(1):24-41. The machinery of macroautophagy. Feng Y, He D, Yao Z, Klionsky DJ. | mTOR and upstream pathways |
| *ATG13* | ENSG00000175224 | KIAA0652|PARATARG8 | 11 | 46638826 | 46696368 | mTORC1 substrates | autophagy related 13 | Cell. 2012 Apr 13;149(2):274-93. mTOR signaling in growth control and disease. Laplante M, Sabatini DM. | mTOR and upstream pathways |
| *LPIN1* | ENSG00000134324 | PAP1 | 2 | 11817721 | 11967535 | mTORC1 substrates | lipin 1 | Cell. 2012 Apr 13;149(2):274-93. mTOR signaling in growth control and disease. Laplante M, Sabatini DM. | mTOR and upstream pathways |
| *AMBRA1* | ENSG00000110497 | DCAF3|WDR94 | 11 | 46417964 | 46615675 | mTORC1 substrates | autophagy/beclin-1 regulator 1 | Nat Cell Biol. 2013 Apr;15(4):406-16. mTOR inhibits autophagy by controlling ULK1 ubiquitylation, self-association and function through AMBRA1 and TRAF6. Nazio F, Strappazzon F, Antonioli M, Bielli P, Cianfanelli V, Bordi M, Gretzmeier C, Dengjel J, Piacentini M, Fimia GM, Cecconi F. | mTOR and upstream pathways |
| *GRB10* | ENSG00000106070 | GRB-IR|Grb-10|IRBP|MEG1|RSS | 7 | 50657760 | 50861159 | mTORC1 substrates | growth factor receptor-bound protein 10 | Nat Rev Mol Cell Biol. 2014 Mar;15(3):155-62. Making new contacts: the mTOR network in metabolism and signalling crosstalk. Shimobayashi M, Hall MN | mTOR and upstream pathways |
| *RPS6KB2* | ENSG00000175634 | KLS|P70-beta|P70-beta-1|P70-beta-2|S6K-beta2|S6K2|S6KB|S6KI(2)|S6Kbeta|SRK|STK14B|p70(S6K)-beta|p70S6Kb | 11 | 67195931 | 67202872 | mTORC1 substrates | ribosomal protein S6 kinase, 70kDa, polypeptide 2 | J Cell Sci. 2013 Apr 15;126(Pt 8):1713-9. Regulation of mTORC1 and its impact on gene expression at a glance. Laplante M, Sabatini DM. | mTOR and upstream pathways |
| *HIF1A* | ENSG00000100644 | HIF-1-alpha|HIF-1A|HIF-1alpha|HIF1|HIF1-ALPHA|MOP1|PASD8|bHLHe78 | 14 | 62162231 | 62214976 | mTORC1 substrates | hypoxia inducible factor 1, alpha subunit (basic helix-loop-helix transcription factor) | J Cell Sci. 2013 Apr 15;126(Pt 8):1713-9. Regulation of mTORC1 and its impact on gene expression at a glance. Laplante M, Sabatini DM. | mTOR and upstream pathways |
| *TFEB* | ENSG00000112561 | ALPHATFEB|BHLHE35|TCFEB | 6 | 41651716 | 41703997 | mTORC1 substrates | transcription factor EB | Science. 2011 Jun 17;332(6036):1429-33. TFEB links autophagy to lysosomal biogenesis. Settembre C, Di Malta C, Polito VA, Garcia Arencibia M, Vetrini F, Erdin S, Erdin SU, Huynh T, Medina D, Colella P, Sardiello M, Rubinsztein DC, Ballabio A. | mTOR and upstream pathways |
| *YY1* | ENSG00000100811 | DELTA|GADEVS|INO80S|NF-E1|UCRBP|YIN-YANG-1 | 14 | 100704635 | 100749129 | mTORC1 substrates | YY1 transcription factor | J Cell Sci. 2013 Apr 15;126(Pt 8):1713-9. Regulation of mTORC1 and its impact on gene expression at a glance. Laplante M, Sabatini DM. | mTOR and upstream pathways |
| *TFE3* | ENSG00000068323 | RCCP2|RCCX1|TFEA|bHLHe33 | X | 48886242 | 48901012 | mTORC1 substrates | transcription factor binding to IGHM enhancer 3 | Sci Signal. 2014 Jan 21;7(309):ra9. The nutrient-responsive transcription factor TFE3 promotes autophagy, lysosomal biogenesis, and clearance of cellular debris. Martina JA, Diab HI, Lishu L, Jeong-A L, Patange S, Raben N, Puertollano R. | mTOR and upstream pathways |
| *STAT3* | ENSG00000168610 | ADMIO|ADMIO1|APRF|HIES | 17 | 40465342 | 40540586 | mTORC1 substrates | signal transducer and activator of transcription 3 (acute-phase response factor) | J Cell Sci. 2013 Apr 15;126(Pt 8):1713-9. Regulation of mTORC1 and its impact on gene expression at a glance. Laplante M, Sabatini DM. | mTOR and upstream pathways |
| *MITF* | ENSG00000187098 | CMM8|COMMAD|MI|WS2|WS2A|bHLHe32 | 3 | 69788586 | 70017488 | mTORC1 substrates | microphthalmia-associated transcription factor | Sci Signal. 2014 Jan 21;7(309):ra9. The nutrient-responsive transcription factor TFE3 promotes autophagy, lysosomal biogenesis, and clearance of cellular debris. Martina JA, Diab HI, Lishu L, Jeong-A L, Patange S, Raben N, Puertollano R. | mTOR and upstream pathways |
| *AKT1S1* | ENSG00000204673 | Lobe|PRAS40 | 19 | 50372295 | 50381716 | mTORC1 substrates | AKT1 substrate 1 (proline-rich) | Nat Rev Mol Cell Biol. 2020 Jan 14. mTOR at the nexus of nutrition, growth, ageing and disease. Liu GY, Sabatini DM. | mTOR and upstream pathways |
| *HDAC5* | ENSG00000108840 | HD5|NY-CO-9 | 17 | 42154114 | 42201070 | mTORC2 substrates | histone deacetylase 5 | Cell Metab. 2013 Nov 5;18(5):726-39. mTOR complex 2 controls glycolytic metabolism in glioblastoma through FoxO acetylation and upregulation of c-Myc. Masui K, Tanaka K, Akhavan D, Babic I, Gini B, Matsutani T, Iwanami A, Liu F, Villa GR, Gu Y, Campos C, Zhu S, Yang H, Yong WH, Cloughesy TF, Mellinghoff IK, Cavenee WK, Shaw RJ, Mischel PS. | mTOR and upstream pathways |
| *HDAC4* | ENSG00000068024 | AHO3|BDMR|HA6116|HD4|HDAC-4|HDAC-A|HDACA | 2 | 239969864 | 240323348 | mTORC2 substrates | histone deacetylase 4 | Cell Metab. 2013 Nov 5;18(5):726-39. mTOR complex 2 controls glycolytic metabolism in glioblastoma through FoxO acetylation and upregulation of c-Myc. Masui K, Tanaka K, Akhavan D, Babic I, Gini B, Matsutani T, Iwanami A, Liu F, Villa GR, Gu Y, Campos C, Zhu S, Yang H, Yong WH, Cloughesy TF, Mellinghoff IK, Cavenee WK, Shaw RJ, Mischel PS. | mTOR and upstream pathways |
| *SGK1* | ENSG00000118515 | SGK | 6 | 134490384 | 134639250 | mTORC2 substrates | serum/glucocorticoid regulated kinase 1 | Cell. 2012 Apr 13;149(2):274-93. mTOR signaling in growth control and disease. Laplante M, Sabatini DM. | mTOR and upstream pathways |
| *PRKCA* | ENSG00000154229 | AAG6|PKC-alpha|PKCA|PKCI+/-|PKCalpha|PRKACA | 17 | 64298754 | 64806861 | mTORC2 substrates | protein kinase C, alpha | Cell. 2012 Apr 13;149(2):274-93. mTOR signaling in growth control and disease. Laplante M, Sabatini DM. | mTOR and upstream pathways |
| *AKT1* | ENSG00000142208 | AKT|PKB|PKB-ALPHA|PRKBA|RAC|RAC-ALPHA | 14 | 105235686 | 105262088 | mTORC2 substrates | v-akt murine thymoma viral oncogene homolog 1 | Nat Rev Mol Cell Biol. 2014 Mar;15(3):155-62. Making new contacts: the mTOR network in metabolism and signalling crosstalk. Shimobayashi M, Hall MN | mTOR and upstream pathways |
| *IGF2BP1* | ENSG00000159217 | CRD-BP|CRDBP|IMP-1|IMP1|VICKZ1|ZBP1 | 17 | 47074774 | 47133012 | mTORC2 substrates | insulin-like growth factor 2 mRNA binding protein 1 | Nat Rev Mol Cell Biol. 2014 Mar;15(3):155-62. Making new contacts: the mTOR network in metabolism and signalling crosstalk. Shimobayashi M, Hall MN | mTOR and upstream pathways |
| *MRAS* | ENSG00000158186 | M-RAs|NS11|R-RAS3|RRAS3 | 3 | 138066539 | 138124375 | RAS family | muscle RAS oncogene homolog | Oncotarget. 2014 Feb 15;5(3):577-86. Ras and autophagy in cancer development and therapy. Schmukler E, Kloog Y, Pinkas-Kramarski R. | mTOR and upstream pathways |
| *KRAS* | ENSG00000133703 | 'C-K-RAS|C-K-RAS|CFC2|K-RAS2A|K-RAS2B|K-RAS4A|K-RAS4B|K-Ras|K-Ras 2|KI-RAS|KRAS1|KRAS2|NS|NS3|OES|RALD|RASK2|c-Ki-ras|c-Ki-ras2 | 12 | 25357723 | 25403870 | RAS family | Kirsten rat sarcoma viral oncogene homolog | Oncotarget. 2014 Feb 15;5(3):577-86. Ras and autophagy in cancer development and therapy. Schmukler E, Kloog Y, Pinkas-Kramarski R. | mTOR and upstream pathways |
| *RRAS* | ENSG00000126458 | R-Ras | 19 | 50138549 | 50143458 | RAS family | related RAS viral (r-ras) oncogene homolog | Oncotarget. 2014 Feb 15;5(3):577-86. Ras and autophagy in cancer development and therapy. Schmukler E, Kloog Y, Pinkas-Kramarski R. | mTOR and upstream pathways |
| *HRAS* | ENSG00000174775|ENSG00000276536 | C-BAS/HAS|C-H-RAS|C-HA-RAS1|CTLO|H-RASIDX|HAMSV|HRAS1|RASH1|p21ras | 11 | 532242 | 537287 | RAS family | Harvey rat sarcoma viral oncogene homolog | Oncotarget. 2014 Feb 15;5(3):577-86. Ras and autophagy in cancer development and therapy. Schmukler E, Kloog Y, Pinkas-Kramarski R. | mTOR and upstream pathways |
| *NRAS* | ENSG00000213281 | ALPS4|CMNS|N-ras|NCMS|NRAS1|NS6 | 1 | 115247090 | 115259515 | RAS family | neuroblastoma RAS viral (v-ras) oncogene homolog | Oncotarget. 2014 Feb 15;5(3):577-86. Ras and autophagy in cancer development and therapy. Schmukler E, Kloog Y, Pinkas-Kramarski R. | mTOR and upstream pathways |
| *ULK3* | ENSG00000140474 | - | 15 | 75128457 | 75135687 | ULK1 complex | unc-51 like kinase 3 | FEBS J. 2016 Nov; 283(21): 3889–3897. Atg1-independent induction of autophagy by the Drosophila Ulk3 homolog, ADUKBraden C R and Neufeld T P | Autophagy core |
| *C12orf44* | ENSG00000123395 | ATG101 | 12 | 52463030 | 52471278 | ULK1 complex | chromosome 12 open reading frame 44 | Autophagy. 2009 Oct;5(7):973–979. Atg101, a novel mammalian autophagy protein interacting with Atg13. Hosokawa N, Sasaki T, Iemura S, Natsume T, Hara T, Mizushima N. | Autophagy core |
| *AMBRA1* | ENSG00000110497 | DCAF3|WDR94 | 11 | 46417964 | 46615675 | ULK1 complex | autophagy/beclin-1 regulator 1 | Nat Cell Biol. 2013 Apr;15(4):406–416mTOR inhibits autophagy by controlling ULK1 ubiquitylation, self-association and function through AMBRA1 and TRAF6. Nazio F, Strappazzon F, Antonioli M, Bielli P, Cianfanelli V, Bordi M, Gretzmeier C, Dengjel J, Piacentini M, Fimia GM, Cecconi F. | Autophagy core |
| *ULK1* | ENSG00000177169 | ATG1|ATG1A|UNC51|Unc51.1|hATG1 | 12 | 132379196 | 132407712 | ULK1 complex | unc-51 like autophagy activating kinase 1 | Cell Res. 2014 Jan;24(1):24–41 The machinery of macroautophagy. Feng Y, He D, Yao Z, Klionsky DJ. | Autophagy core |
| *TRAF6* | ENSG00000175104 | MGC:3310|RNF85 | 11 | 36508577 | 36531822 | ULK1 complex | TNF receptor-associated factor 6, E3 ubiquitin protein ligase | Nat Cell Biol. 2013 Apr;15(4):406–416mTOR inhibits autophagy by controlling ULK1 ubiquitylation, self-association and function through AMBRA1 and TRAF6. Nazio F, Strappazzon F, Antonioli M, Bielli P, Cianfanelli V, Bordi M, Gretzmeier C, Dengjel J, Piacentini M, Fimia GM, Cecconi F. | Autophagy core |
| *RB1CC1* | ENSG00000023287 | ATG17|CC1|FIP200|PPP1R131 | 8 | 53535016 | 53658403 | ULK1 complex | RB1-inducible coiled-coil 1 | Cell Res. 2014 Jan;24(1):24–41 The machinery of macroautophagy. Feng Y, He D, Yao Z, Klionsky DJ. | Autophagy core |
| *ULK2* | ENSG00000083290 | ATG1B|Unc51.2 | 17 | 19674142 | 19771249 | ULK1 complex | unc-51 like autophagy activating kinase 2 | Autophagy. 2011 Jul; 7(7): 689–695. The requirement of uncoordinated 51-like kinase 1 (ULK1) and ULK2 in the regulation of autophagyLee E J and Tournier C | Autophagy core |
| *ATG13* | ENSG00000175224 | KIAA0652|PARATARG8 | 11 | 46638826 | 46696368 | ULK1 complex | autophagy related 13 | Cell Res. 2014 Jan;24(1):24–41 The machinery of macroautophagy. Feng Y, He D, Yao Z, Klionsky DJ. | Autophagy core |
| *BECN2* | ENSG00000196289 | BECLIN2 | 1 | 242121039 | 242122364 | PI3K complexes | beclin 1, autophagy related, pseudogene 1 | Cell Res. 2014 Jan;24(1):24–41 The machinery of macroautophagy. Feng Y, He D, Yao Z, Klionsky DJ. J Cell Sci. 2012 May 15;125(Pt10):2343–2348Ubiquitin-like proteins and autophagy at a glance. Shpilka T, Mizushima N, Elazar Z. | Autophagy core |
| *BECN1* | ENSG00000126581 | ATG6|VPS30|beclin1 | 17 | 40962152 | 40985367 | PI3K complexes | beclin 1, autophagy related | Cell Res. 2014 Jan;24(1):24–41 The machinery of macroautophagy. Feng Y, He D, Yao Z, Klionsky DJ. J Cell Sci. 2012 May 15;125(Pt10):2343–2348Ubiquitin-like proteins and autophagy at a glance. Shpilka T, Mizushima N, Elazar Z. | Autophagy core |
| *PIK3C3* | ENSG00000078142 | VPS34|Vps34|hVps34 | 18 | 39535171 | 39667794 | PI3K complexes | phosphatidylinositol 3-kinase, catalytic subunit type 3 | Cell Res. 2014 Jan;24(1):24–41 The machinery of macroautophagy. Feng Y, He D, Yao Z, Klionsky DJ. J Cell Sci. 2012 May 15;125(Pt10):2343–2348Ubiquitin-like proteins and autophagy at a glance. Shpilka T, Mizushima N, Elazar Z. | Autophagy core |
| *PIK3R4* | ENSG00000196455 | VPS15|p150 | 3 | 130397779 | 130465673 | PI3K complexes | phosphoinositide-3-kinase, regulatory subunit 4 | Cell Res. 2014 Jan;24(1):24–41 The machinery of macroautophagy. Feng Y, He D, Yao Z, Klionsky DJ. J Cell Sci. 2012 May 15;125(Pt10):2343–2348Ubiquitin-like proteins and autophagy at a glance. Shpilka T, Mizushima N, Elazar Z. | Autophagy core |
| *ATG14* | ENSG00000126775 | ATG14L|BARKOR|KIAA0831 | 14 | 55833110 | 55878576 | Beclin1/PI3K complex interacting proteins | autophagy related 14 | Nat Rev Mol Cell Biol. 2018 Jun;19(6):349–364. Mechanism and medical implications of mammalian autophagy. Dikic I, Elazar Z. | Autophagy core |
| *UVRAG* | ENSG00000198382 | DHTX|VPS38|p63 | 11 | 75526212 | 75854239 | Beclin1/PI3K complex interacting proteins | UV radiation resistance associated | Nat Rev Mol Cell Biol. 2018 Jun;19(6):349–364. Mechanism and medical implications of mammalian autophagy. Dikic I, Elazar Z. | Autophagy core |
| *KIAA0226* | ENSG00000145016 | RUBICON | 3 | 197398264 | 197476598 | Beclin1/PI3K complex interacting proteins | KIAA0226 | Nat Rev Mol Cell Biol. 2018 Jun;19(6):349–364. Mechanism and medical implications of mammalian autophagy. Dikic I, Elazar Z. | Autophagy core |
| *RNF2* | ENSG00000121481 | BAP-1|BAP1|DING|HIPI3|RING1B|RING2 | 1 | 185014496 | 185071740 | Beclin1/PI3K complex interacting proteins | ring finger protein 2 | Cell Res. 2014 Aug;24(8):943–958RNF2 is recruited by WASH to ubiquitinate AMBRA1 leading to downregulation of autophagy. Xia P, Wang S, Huang G, Du Y, Zhu P, Li M, Fan Z. | Autophagy core |
| *TLR4* | ENSG00000136869 | ARMD10|CD284|TLR-4|TOLL | 9 | 120466610 | 120479149 | Beclin1/PI3K complex interacting proteins | toll-like receptor 4 | Sci Signal. 2010 May 25;3(123):ra42. TRAF6 and A20 regulate lysine 63-linked ubiquitination of Beclin-1 to control TLR4-induced autophagy. Shi CS, Kehrl JH. | Autophagy core |
| *SH3GLB1* | ENSG00000097033 | Bif-1|CGI-61|PPP1R70|dJ612B15.2 | 1 | 87170259 | 87213867 | Beclin1/PI3K complex interacting proteins | SH3-domain GRB2-like endophilin B1 | Nat Rev Mol Cell Biol. 2018 Jun;19(6):349–364. Mechanism and medical implications of mammalian autophagy. Dikic I, Elazar Z. | Autophagy core |
| *VMP1* | ENSG00000062716 | EPG3|TANGO5|TMEM49 | 17 | 57784553 | 57919616 | Beclin1/PI3K complex interacting proteins | vacuole membrane protein 1 | Nat Rev Mol Cell Biol. 2018 Jun;19(6):349–364. Mechanism and medical implications of mammalian autophagy. Dikic I, Elazar Z. | Autophagy core |
| *BCL2L1* | ENSG00000171552 | BCL-XL/S|BCL2L|BCLX|Bcl-X|PPP1R52 | 20 | 30252255 | 30311792 | Beclin1/PI3K complex interacting proteins | BCL2-like 1 | J Biol Chem. 2008 Sep 19;283(38):26274–26282. Bcl-xL and UVRAG cause a monomer-dimer switch in Beclin1. Noble CG, Dong JM, Manser E, Song H. | Autophagy core |
| *BCL2* | ENSG00000171791 | Bcl-2|PPP1R50 | 18 | 60790579 | 60987361 | Beclin1/PI3K complex interacting proteins | B-cell CLL/lymphoma 2 | Nat Rev Mol Cell Biol. 2018 Jun;19(6):349–364. Mechanism and medical implications of mammalian autophagy. Dikic I, Elazar Z. | Autophagy core |
| *BIRC5* | ENSG00000089685 | API4|EPR-1 | 17 | 76210267 | 76221717 | Beclin1/PI3K complex interacting proteins | baculoviral IAP repeat containing 5 | Nat Rev Mol Cell Biol. 2018 Jun;19(6):349–364. Mechanism and medical implications of mammalian autophagy. Dikic I, Elazar Z. | Autophagy core |
| *HMGB1* | ENSG00000189403 | HMG-1|HMG1|HMG3|SBP-1 | 13 | 31032884 | 31191734 | Beclin1/PI3K complex interacting proteins | high mobility group box 1 | Nat Rev Mol Cell Biol. 2018 Jun;19(6):349–364. Mechanism and medical implications of mammalian autophagy. Dikic I, Elazar Z. | Autophagy core |
| *RHOQ* | ENSG00000119729 | ARHQ|HEL-S-42|RASL7A|TC10|TC10A | 2 | 46768945 | 46810260 | Beclin1/PI3K complex interacting proteins | ras homolog family member Q | Nat Rev Mol Cell Biol. 2018 Jun;19(6):349–364. Mechanism and medical implications of mammalian autophagy. Dikic I, Elazar Z. | Autophagy core |
| *WAS* | ENSG00000015285 | IMD2|SCNX|THC|THC1|WASP|WASPA | X | 48534985 | 48549818 | Beclin1/PI3K complex interacting proteins | Wiskott-Aldrich syndrome | Cell Res. 2014 Aug;24(8):943–958RNF2 is recruited by WASH to ubiquitinate AMBRA1 leading to downregulation of autophagy. Xia P, Wang S, Huang G, Du Y, Zhu P, Li M, Fan Z. | Autophagy core |
| *PAQR3* | ENSG00000163291 | RKTG | 4 | 79808281 | 79860592 | Beclin1/PI3K complex interacting proteins | progestin and adipoQ receptor family member III | EMBO J. 2016 Mar 1;35(5):496–514PAQR3 controls autophagy by integrating AMPK signaling to enhance ATG14L-associated PI3K activity. Xu DQ, Wang Z, Wang CY, Zhang DY, Wan HD, Zhao ZL, Gu J, Zhang YX, Li ZG, Man KY, Pan Y, Wang ZF, Ke ZJ, Liu ZX, Liao LJ, Chen Y. | Autophagy core |
| *AMBRA1* | ENSG00000110497 | DCAF3|WDR94 | 11 | 46417964 | 46615675 | Beclin1/PI3K complex interacting proteins | autophagy/beclin-1 regulator 1 | Nat Rev Mol Cell Biol. 2018 Jun;19(6):349–364. Mechanism and medical implications of mammalian autophagy. Dikic I, Elazar Z. | Autophagy core |
| *SLAMF1* | ENSG00000117090 | CD150|CDw150|SLAM | 1 | 160577890 | 160617085 | Beclin1/PI3K complex interacting proteins | signaling lymphocytic activation molecule family member 1 | Nat Rev Mol Cell Biol. 2018 Jun;19(6):349–364. Mechanism and medical implications of mammalian autophagy. Dikic I, Elazar Z. | Autophagy core |
| *ITPR3* | ENSG00000096433 | IP3R|IP3R3 | 6 | 33588142 | 33664351 | Beclin1/PI3K complex interacting proteins | inositol 1,4,5-trisphosphate receptor, type 3 | Nat Rev Mol Cell Biol. 2018 Jun;19(6):349–364. Mechanism and medical implications of mammalian autophagy. Dikic I, Elazar Z. | Autophagy core |
| *PINK1* | ENSG00000158828 | BRPK|PARK6 | 1 | 20959948 | 20978004 | Beclin1/PI3K complex interacting proteins | PTEN induced putative kinase 1 | Cell Death Differ. 2010 Jun;17(6):962–974The Parkinson-associated protein PINK1 interacts with Beclin1 and promotes autophagy. Michiorri S, Gelmetti V, Giarda E, Lombardi F, Romano F, Marongiu R, Nerini-Molteni S, Sale P, Vago R, Arena G, Torosantucci L, Cassina L, Russo MA, Dallapiccola B, Valente EM, Casari G. | Autophagy core |
| *STK38* | ENSG00000112079 | NDR|NDR1 | 6 | 36461669 | 36515247 | Beclin1/PI3K complex interacting proteins | Serine/Threonine Kinase 38 | Curr Biol. 2015 Oct 5;25(19):2479–2492The Pro-apoptotic STK38 Kinase Is a New Beclin1 Partner Positively Regulating Autophagy. Joffre C, Dupont N, Hoa L, Gomez V, Pardo R, Gonçalves-Pimentel C, Achard P, Bettoun A, Meunier B, Bauvy C, Cascone I, Codogno P, Fanto M, Hergovich A, Camonis J. | Autophagy core |
| *NEDD4* | ENSG00000069869 | NEDD4-1|RPF1 | 15 | 56119120 | 56285944 | Beclin1/PI3K complex interacting proteins | neural precursor cell expressed, developmentally down-regulated 4, E3 ubiquitin protein ligase | FEBS Lett. 2012 Jun 4;586(11):1584–1591Ubiquitination and phosphorylation of Beclin 1 and its binding partners: Tuning class III phosphatidylinositol 3-kinase activity and tumor suppression. Abrahamsen H, Stenmark H, Platta HW. | Autophagy core |
| *GABARAPL1* | ENSG00000139112 | APG8-LIKE|APG8L|ATG8|ATG8B|ATG8L|GEC1 | 12 | 10365057 | 10375727 | ATG8 Ubiquitin-like conjugation systems | GABA(A) receptor-associated protein like 1 | Cell Res. 2014 Jan;24(1):24–41 The machinery of macroautophagy. Feng Y, He D, Yao Z, Klionsky DJ. J Cell Sci. 2012 May 15;125(Pt10):2343–2348Ubiquitin-like proteins and autophagy at a glance. Shpilka T, Mizushima N, Elazar Z. | Autophagy core |
| *ATG4A* | ENSG00000101844 | APG4A|AUTL2 | X | 107334898 | 107397901 | ATG8 Ubiquitin-like conjugation systems | autophagy related 4A, cysteine peptidase | Cell Res. 2014 Jan;24(1):24–41 The machinery of macroautophagy. Feng Y, He D, Yao Z, Klionsky DJ. J Cell Sci. 2012 May 15;125(Pt10):2343–2348Ubiquitin-like proteins and autophagy at a glance. Shpilka T, Mizushima N, Elazar Z. | Autophagy core |
| *ATG3* | ENSG00000144848 | APG3|APG3-LIKE|APG3L|PC3-96 | 3 | 112251356 | 112280893 | ATG8 Ubiquitin-like conjugation systems | autophagy related 3 | Cell Res. 2014 Jan;24(1):24–41 The machinery of macroautophagy. Feng Y, He D, Yao Z, Klionsky DJ. J Cell Sci. 2012 May 15;125(Pt10):2343–2348Ubiquitin-like proteins and autophagy at a glance. Shpilka T, Mizushima N, Elazar Z. | Autophagy core |
| *ATG7* | ENSG00000197548 | APG7-LIKE|APG7L|GSA7 | 3 | 11313995 | 11599139 | ATG8 Ubiquitin-like conjugation systems | autophagy related 7 | Cell Res. 2014 Jan;24(1):24–41 The machinery of macroautophagy. Feng Y, He D, Yao Z, Klionsky DJ. J Cell Sci. 2012 May 15;125(Pt10):2343–2348Ubiquitin-like proteins and autophagy at a glance. Shpilka T, Mizushima N, Elazar Z. | Autophagy core |
| *MAP1LC3C* | ENSG00000197769 | ATG8J|LC3C | 1 | 242158792 | 242162375 | ATG8 Ubiquitin-like conjugation systems | microtubule-associated protein 1 light chain 3 gamma | Cell Res. 2014 Jan;24(1):24–41 The machinery of macroautophagy. Feng Y, He D, Yao Z, Klionsky DJ. J Cell Sci. 2012 May 15;125(Pt10):2343–2348Ubiquitin-like proteins and autophagy at a glance. Shpilka T, Mizushima N, Elazar Z. | Autophagy core |
| *MAP1LC3B* | ENSG00000140941 | ATG8F|LC3B|MAP1A/1BLC3|MAP1LC3B-a | 16 | 87417601 | 87438385 | ATG8 Ubiquitin-like conjugation systems | microtubule-associated protein 1 light chain 3 beta | Cell Res. 2014 Jan;24(1):24–41 The machinery of macroautophagy. Feng Y, He D, Yao Z, Klionsky DJ. J Cell Sci. 2012 May 15;125(Pt10):2343–2348Ubiquitin-like proteins and autophagy at a glance. Shpilka T, Mizushima N, Elazar Z. | Autophagy core |
| *GABARAPL2* | ENSG00000034713 | ATG8|ATG8C|GATE-16|GATE16|GEF-2|GEF2 | 16 | 75600249 | 75611779 | ATG8 Ubiquitin-like conjugation systems | GABA(A) receptor-associated protein-like 2 | Cell Res. 2014 Jan;24(1):24–41 The machinery of macroautophagy. Feng Y, He D, Yao Z, Klionsky DJ. J Cell Sci. 2012 May 15;125(Pt10):2343–2348Ubiquitin-like proteins and autophagy at a glance. Shpilka T, Mizushima N, Elazar Z. | Autophagy core |
| *ATG4B* | ENSG00000168397 | APG4B|AUTL1 | 2 | 242576628 | 242613272 | ATG8 Ubiquitin-like conjugation systems | autophagy related 4B, cysteine peptidase | Cell Res. 2014 Jan;24(1):24–41 The machinery of macroautophagy. Feng Y, He D, Yao Z, Klionsky DJ. J Cell Sci. 2012 May 15;125(Pt10):2343–2348Ubiquitin-like proteins and autophagy at a glance. Shpilka T, Mizushima N, Elazar Z. | Autophagy core |
| *MAP1LC3A* | ENSG00000101460 | ATG8E|LC3|LC3A|MAP1ALC3|MAP1BLC3 | 20 | 33134658 | 33148149 | ATG8 Ubiquitin-like conjugation systems | microtubule-associated protein 1 light chain 3 alpha | Cell Res. 2014 Jan;24(1):24–41 The machinery of macroautophagy. Feng Y, He D, Yao Z, Klionsky DJ. J Cell Sci. 2012 May 15;125(Pt10):2343–2348Ubiquitin-like proteins and autophagy at a glance. Shpilka T, Mizushima N, Elazar Z. | Autophagy core |
| *ATG4C* | ENSG00000125703 | APG4-C|APG4C|AUTL1|AUTL3 | 1 | 63249806 | 63331184 | ATG8 Ubiquitin-like conjugation systems | autophagy related 4C, cysteine peptidase | Cell Res. 2014 Jan;24(1):24–41 The machinery of macroautophagy. Feng Y, He D, Yao Z, Klionsky DJ. J Cell Sci. 2012 May 15;125(Pt10):2343–2348Ubiquitin-like proteins and autophagy at a glance. Shpilka T, Mizushima N, Elazar Z. | Autophagy core |
| *ATG4D* | ENSG00000130734 | APG4-D|APG4D|AUTL4 | 19 | 10654571 | 10664094 | ATG8 Ubiquitin-like conjugation systems | autophagy related 4D, cysteine peptidase | Cell Res. 2014 Jan;24(1):24–41 The machinery of macroautophagy. Feng Y, He D, Yao Z, Klionsky DJ. J Cell Sci. 2012 May 15;125(Pt10):2343–2348Ubiquitin-like proteins and autophagy at a glance. Shpilka T, Mizushima N, Elazar Z. | Autophagy core |
| *GABARAP* | ENSG00000170296 | ATG8A|GABARAP-a|MM46 | 17 | 7143333 | 7146089 | ATG8 Ubiquitin-like conjugation systems | GABA(A) receptor-associated protein | Cell Res. 2014 Jan;24(1):24–41 The machinery of macroautophagy. Feng Y, He D, Yao Z, Klionsky DJ. J Cell Sci. 2012 May 15;125(Pt10):2343–2348Ubiquitin-like proteins and autophagy at a glance. Shpilka T, Mizushima N, Elazar Z. | Autophagy core |
| *MAP1LC3B2* | ENSG00000258102 | ATG8G | 12 | 116997186 | 117014425 | ATG8 Ubiquitin-like conjugation systems | microtubule-associated protein 1 light chain 3 beta 2 | Cell Res. 2014 Jan;24(1):24–41 The machinery of macroautophagy. Feng Y, He D, Yao Z, Klionsky DJ. J Cell Sci. 2012 May 15;125(Pt10):2343–2348Ubiquitin-like proteins and autophagy at a glance. Shpilka T, Mizushima N, Elazar Z. | Autophagy core |
| *GABARAPL3* | ENSG00000238244 | GABARAPL3 | 15 | 90890819 | 90892669 | ATG8 Ubiquitin-like conjugation systems | GABA(A) receptors associated protein like 3, pseudogene | Cell Res. 2014 Jan;24(1):24–41 The machinery of macroautophagy. Feng Y, He D, Yao Z, Klionsky DJ. J Cell Sci. 2012 May 15;125(Pt10):2343–2348Ubiquitin-like proteins and autophagy at a glance. Shpilka T, Mizushima N, Elazar Z. | Autophagy core |
| *ATG2B* | ENSG00000066739 | C14orf103 | 14 | 96747595 | 96830207 | ATG9 and its cycling system | autophagy related 2B | Cell Res. 2014 Jan;24(1):24–41 The machinery of macroautophagy. Feng Y, He D, Yao Z, Klionsky DJ. | Autophagy core |
| *ATG2A* | ENSG00000110046 | - | 11 | 64662007 | 64684722 | ATG9 and its cycling system | autophagy related 2A | Cell Res. 2014 Jan;24(1):24–41 The machinery of macroautophagy. Feng Y, He D, Yao Z, Klionsky DJ. | Autophagy core |
| *WIPI2* | ENSG00000157954 | ATG18B|Atg21|CGI-50|IDDSSA|WIPI-2 | 7 | 5229819 | 5273457 | ATG9 and its cycling system | WD repeat domain, phosphoinositide interacting 2 | Autophagy. 2010 May;6(4):506–522. Mammalian Atg18 (WIPI2) localizes to omegasome-anchored phagophores and positively regulates LC3 lipidation. Polson HE, de Lartigue J, Rigden DJ, Reedijk M, Urbé S, Clague MJ, Tooze SA. | Autophagy core |
| *WIPI1* | ENSG00000070540 | ATG18|ATG18A|WIPI49 | 17 | 66417089 | 66453654 | ATG9 and its cycling system | WD repeat domain, phosphoinositide interacting 1 | Methods Enzymol. 2009;452:247–260Assessing mammalian autophagy by WIPI-1/Atg18 puncta formation. Proikas-Cezanne T, Pfisterer SG. | Autophagy core |
| *ATG9A* | ENSG00000198925 | APG9L1|MGD3208|mATG9 | 2 | 220074494 | 220094439 | ATG9 and its cycling system | autophagy related 9A | Cell Res. 2014 Jan;24(1):24–41 The machinery of macroautophagy. Feng Y, He D, Yao Z, Klionsky DJ. | Autophagy core |
| *ATG9B* | ENSG00000181652 | APG9L2|NOS3AS|SONE | 7 | 150709297 | 150721586 | ATG9 and its cycling system | autophagy related 9B | Cell Res. 2014 Jan;24(1):24–41 The machinery of macroautophagy. Feng Y, He D, Yao Z, Klionsky DJ. | Autophagy core |
| *WDR45B* | ENSG00000141580 | NEDSBAS|WDR45L|WIPI-3|WIPI3 | 17 | 80572438 | 80606429 | ATG9 and its cycling system | WD repeat domain 45B | Mol Cell. 2014 Jul 17;55(2):238–252. WIPI2 links LC3 conjugation with PI3P, autophagosome formation, and pathogen clearance by recruiting Atg12-5-16L1. Dooley HC, Razi M, Polson HE, Girardin SE, Wilson MI, Tooze SA. | Autophagy core |
| *WDR45* | ENSG00000196998 | JM5|NBIA4|NBIA5|WDRX1|WIPI-4|WIPI4 | X | 48929385 | 48958108 | ATG9 and its cycling system | WD repeat domain 45 | Autophagy. 2015;11(6):881–890The autophagy gene Wdr45/Wipi4 regulates learning and memory function and axonal homeostasis. Zhao YG, Sun L, Miao G, Ji C, Zhao H, Sun H, Miao L, Yoshii SR, Mizushima N, Wang X, Zhang H. | Autophagy core |
| *ATG12* | ENSG00000145782 | APG12|APG12L|FBR93|HAPG12 | 5 | 115163893 | 115177555 | ATG12 Ubiquitin-like conjugation systems | autophagy related 12 | Cell Res. 2014 Jan;24(1):24–41 The machinery of macroautophagy. Feng Y, He D, Yao Z, Klionsky DJ. J Cell Sci. 2012 May 15;125(Pt10):2343–2348Ubiquitin-like proteins and autophagy at a glance. Shpilka T, Mizushima N, Elazar Z. | Autophagy core |
| *ATG7* | ENSG00000197548 | APG7-LIKE|APG7L|GSA7 | 3 | 11313995 | 11599139 | ATG12 Ubiquitin-like conjugation systems | autophagy related 7 | Cell Res. 2014 Jan;24(1):24–41 The machinery of macroautophagy. Feng Y, He D, Yao Z, Klionsky DJ. J Cell Sci. 2012 May 15;125(Pt10):2343–2348Ubiquitin-like proteins and autophagy at a glance. Shpilka T, Mizushima N, Elazar Z. | Autophagy core |
| *ATG16L1* | ENSG00000085978|ENSG00000281089 | APG16L|ATG16A|ATG16L|IBD10|WDR30 | 2 | 234118697 | 234204320 | ATG12 Ubiquitin-like conjugation systems | autophagy related 16-like 1 (S. cerevisiae) | Cell Res. 2014 Jan;24(1):24–41 The machinery of macroautophagy. Feng Y, He D, Yao Z, Klionsky DJ. J Cell Sci. 2012 May 15;125(Pt10):2343–2348Ubiquitin-like proteins and autophagy at a glance. Shpilka T, Mizushima N, Elazar Z. | Autophagy core |
| *ATG5* | ENSG00000057663 | APG5|APG5-LIKE|APG5L|ASP|SCAR25|hAPG5 | 6 | 106632351 | 106773666 | ATG12 Ubiquitin-like conjugation systems | autophagy related 5 | Cell Res. 2014 Jan;24(1):24–41 The machinery of macroautophagy. Feng Y, He D, Yao Z, Klionsky DJ. J Cell Sci. 2012 May 15;125(Pt10):2343–2348Ubiquitin-like proteins and autophagy at a glance. Shpilka T, Mizushima N, Elazar Z. | Autophagy core |
| *ATG10* | ENSG00000152348 | APG10|APG10L|pp12616 | 5 | 81267844 | 81572676 | ATG12 Ubiquitin-like conjugation systems | autophagy related 10 | Cell Res. 2014 Jan;24(1):24–41 The machinery of macroautophagy. Feng Y, He D, Yao Z, Klionsky DJ. J Cell Sci. 2012 May 15;125(Pt10):2343–2348Ubiquitin-like proteins and autophagy at a glance. Shpilka T, Mizushima N, Elazar Z. | Autophagy core |
| *ATG16L2* | ENSG00000168010 | ATG16B|WDR80 | 11 | 72525353 | 72554719 | ATG12 Ubiquitin-like conjugation systems | autophagy related 16-like 2 (S. cerevisiae) | Cell Res. 2014 Jan;24(1):24–41 The machinery of macroautophagy. Feng Y, He D, Yao Z, Klionsky DJ. J Cell Sci. 2012 May 15;125(Pt10):2343–2348Ubiquitin-like proteins and autophagy at a glance. Shpilka T, Mizushima N, Elazar Z. | Autophagy core |
| *SQSTM1* | ENSG00000161011|ENSG00000284099 | A170|DMRV|FTDALS3|NADGP|OSIL|PDB3|ZIP3|p60|p62|p62B | 5 | 179233388 | 179265078 | Cargo receptors for Selective Autophagy | sequestosome 1 | Nat Rev Mol Cell Biol. 2018 Jun;19(6):349-364. Mechanism and medical implications of mammalian autophagy. Dikic I, Elazar Z. | Autophagy core |
| *OPTN* | ENSG00000123240 | ALS12|FIP2|GLC1E|HIP7|HYPL|NRP|TFIIIA-INTP | 10 | 13141449 | 13180291 | Cargo receptors for Selective Autophagy | optineurin | Nat Rev Mol Cell Biol. 2018 Jun;19(6):349-364. Mechanism and medical implications of mammalian autophagy. Dikic I, Elazar Z. | Autophagy core |
| *NBR1* | ENSG00000188554 | 1A1-3B|IAI3B|M17S2|MIG19 | 17 | 41322498 | 41363708 | Cargo receptors for Selective Autophagy | neighbor of BRCA1 gene 1 | Nat Rev Mol Cell Biol. 2018 Jun;19(6):349-364. Mechanism and medical implications of mammalian autophagy. Dikic I, Elazar Z. | Autophagy core |
| *TAX1BP1* | ENSG00000106052 | CALCOCO3|T6BP|TXBP151 | 7 | 27778950 | 27884183 | Cargo receptors for Selective Autophagy | Tax1 (human T-cell leukemia virus type I) binding protein 1 | J Cell Sci. 2013 Aug 1;126(Pt 15):3237–3247The LIR motif - crucial for selective autophagy. Birgisdottir ÅB, Lamark T, Johansen T. | Autophagy core |
| *CBL* | ENSG00000110395 | C-CBL|CBL2|FRA11B|NSLL|RNF55 | 11 | 119076752 | 119178859 | Cargo receptors for Selective Autophagy | Cbl proto-oncogene, E3 ubiquitin protein ligase | Cell Death Differ. 2013 Jan;20(1):21–30. Ubiquitination and selective autophagy. Shaid S, Brandts CH, Serve H, Dikic I. | Autophagy core |
| *CALCOCO2* | ENSG00000136436 | NDP52 | 17 | 46908350 | 46943884 | Cargo receptors for Selective Autophagy | calcium binding and coiled-coil domain 2 | Nat Rev Mol Cell Biol. 2018 Jun;19(6):349-364. Mechanism and medical implications of mammalian autophagy. Dikic I, Elazar Z. | Autophagy core |
| *BNIP3L* | ENSG00000104765 | BNIP3a|NIX | 8 | 26240414 | 26363152 | Cargo receptors for Selective Autophagy | BCL2/adenovirus E1B 19kDa interacting protein 3-like | Nat Cell Biol. 2014 Jun;16(6):495–501Cargo recognition and trafficking in selective autophagy. Stolz A, Ernst A, Dikic I. | Autophagy core |
| *WDFY3* | ENSG00000163625 | ALFY|BCHS|MCPH18|ZFYVE25 | 4 | 85590704 | 85887544 | Cargo indirectly associated with ubiquitinated proteins or with LC3 | WD repeat and FYVE domain containing 3 | Cell Death Differ. 2013 Jan;20(1):21–30. Ubiquitination and selective autophagy. Shaid S, Brandts CH, Serve H, Dikic I. | Autophagy core |
| *BAG3* | ENSG00000151929 | BAG-3|BIS|CAIR-1|MFM6 | 10 | 121410882 | 121437331 | Cargo indirectly associated with ubiquitinated proteins or with LC3 | BCL2-associated athanogene 3 | Cell Death Differ. 2013 Jan;20(1):21–30. Ubiquitination and selective autophagy. Shaid S, Brandts CH, Serve H, Dikic I. | Autophagy core |
| *TECPR1* | ENSG00000205356 | - | 7 | 97843936 | 97881563 | Cargo indirectly associated with ubiquitinated proteins or with LC3 | tectonin beta-propeller repeat containing 1 | Cell Death Differ. 2013 Jan;20(1):21–30. Ubiquitination and selective autophagy. Shaid S, Brandts CH, Serve H, Dikic I. | Autophagy core |
| *HDAC6* | ENSG00000094631 | CPBHM|HD6|JM21|PPP1R90 | X | 48659784 | 48683392 | Cargo indirectly associated with ubiquitinated proteins or with LC3 | histone deacetylase 6 | Cell Death Differ. 2013 Jan;20(1):21–30. Ubiquitination and selective autophagy. Shaid S, Brandts CH, Serve H, Dikic I. | Autophagy core |
| *NEDD4L* | ENSG00000049759 | NEDD4-2|NEDD4.2|PVNH7|RSP5|hNEDD4-2 | 18 | 55711599 | 56068772 | Negative Regulator of Autophagy | neural precursor cell expressed, developmentally down-regulated 4-like, E3 ubiquitin protein ligase | J Cell Biol. 2016 Dec 19;215(6):841–856Fine-tuning of ULK1 mRNA and protein levels is required for autophagy oscillation. Nazio F, Carinci M, Valacca C, Bielli P, Strappazzon F, Antonioli M, Ciccosanti F, Rodolfo C, Campello S, Fimia GM, Sette C, Bonaldo P, Cecconi F. | Autophagy core |
| *CUL4A* | ENSG00000139842 | - | 13 | 113862552 | 113919399 | Negative Regulator of Autophagy | cullin 4A | Mol Cell Oncol. 2015 Feb 3;3(5):e1008304. Temporal regulation of autophagy response by the CULLIN 4-AMBRA1-CULLIN 5 axis. Antonioli M, Albiero F, Piacentini M, Fimia GM. | Autophagy core |
| *CAPN2* | ENSG00000162909 | CANP2|CANPL2|CANPml|mCANP | 1 | 223889295 | 223963720 | Negative Regulator of Autophagy | calpain 2, (m/II) large subunit | Cell Death Dis. 2016 Apr 14;7(4):e2186Calpain 2-mediated autophagy defect increases susceptibility of fatty livers to ischemia-reperfusion injury. Zhao Q, Guo Z, Deng W, Fu S, Zhang C, Chen M, Ju W, Wang D, He X. | Autophagy core |
| *CAPNS1* | ENSG00000126247 | CALPAIN4|CANP|CANPS|CAPN4|CDPS|CSS1 | 19 | 36630477 | 36641255 | Negative Regulator of Autophagy | calpain, small subunit 1 | Nat Cell Biol. 2006;8(10):1124–1132. Calpain-mediated cleavage of Atg5 switches autophagy to apoptosis. Yousefi S, Perozzo R, Schmid I, Ziemiecki A, Schaffner T, Scapozza L, Brunner T, Simon HU. | Autophagy core |
| *FEZ1* | ENSG00000149557 | UNC-76 | 11 | 125315646 | 125366213 | Negative Regulator of Autophagy | fasciculation and elongation protein zeta 1 (zygin I) | EMBO J. 2012 Apr 18;31(8):1931–1946Genome-wide siRNA screen reveals amino acid starvation-induced autophagy requires SCOC and WAC. McKnight NC, Jefferies HB, Alemu EA, Saunders RE, Howell M, Johansen T, Tooze SA. | Autophagy core |
| *STK39* | ENSG00000198648 | DCHT|PASK|SPAK | 2 | 168810530 | 169104651 | Negative Regulator of Autophagy | serine threonine kinase 39 | Proc Natl Acad Sci U S A. 2016 Dec 13;113(50):14342–14347Multistep regulation of autophagy by WNK1. Gallolu Kankanamalage S, Lee AY, Wichaidit C, Lorente-Rodriguez A, Shah AM, Stippec S, Whitehurst AW, Cobb MH. | Autophagy core |
| *WNK1* | ENSG00000060237 | HSAN2|HSN2|KDP|PPP1R167|PRKWNK1|PSK|p65 | 12 | 861759 | 1020618 | Negative Regulator of Autophagy | WNK lysine deficient protein kinase 1 | Proc Natl Acad Sci U S A. 2016 Dec 13;113(50):14342–14347Multistep regulation of autophagy by WNK1. Gallolu Kankanamalage S, Lee AY, Wichaidit C, Lorente-Rodriguez A, Shah AM, Stippec S, Whitehurst AW, Cobb MH. | Autophagy core |
| *CUL4B* | ENSG00000158290 | CUL-4B|MRXHF2|MRXS15|MRXSC|SFM2 | X | 119658464 | 119709649 | Negative Regulator of Autophagy | cullin 4B | Mol Cell Oncol. 2015 Feb 3;3(5):e1008304. Temporal regulation of autophagy response by the CULLIN 4-AMBRA1-CULLIN 5 axis. Antonioli M, Albiero F, Piacentini M, Fimia GM. | Autophagy core |
| *CUL3* | ENSG00000036257 | CUL-3|PHA2E | 2 | 225334867 | 225450110 | Negative Regulator of Autophagy | cullin 3 | Mol Cell. 2016 Jan 7;61(1):84–97Cul3-KLHL20 Ubiquitin Ligase Governs the Turnover of ULK1 and VPS34 Complexes to Control Autophagy Termination. Liu CC, Lin YC, Chen YH, Chen CM, Pang LY, Chen HA, Wu PR, Lin MY, Jiang ST, Tsai TF, Chen RH. | Autophagy core |
| *CUL5* | ENSG00000166266 | CUL-5|VACM-1|VACM1 | 11 | 107879459 | 107978503 | Negative Regulator of Autophagy | cullin 5 | Mol Cell Oncol. 2015 Feb 3;3(5):e1008304. Temporal regulation of autophagy response by the CULLIN 4-AMBRA1-CULLIN 5 axis. Antonioli M, Albiero F, Piacentini M, Fimia GM. | Autophagy core |
| *CAPN1* | ENSG00000014216 | CANP|CANP1|CANPL1|SPG76|muCANP|muCL | 11 | 64948037 | 64979477 | Negative Regulator of Autophagy | calpain 1, (mu/I) large subunit, | Autophagy. 2010 Jan;6(1):61–66Control of basal autophagy by calpain1 mediated cleavage of ATG5. Xia HG, Zhang L, Chen G, Zhang T, Liu J, Jin M, Ma X, Ma D, Yuan J. | Autophagy core |
| *MAPK14* | ENSG00000112062 | CSBP|CSBP1|CSBP2|CSPB1|EXIP|Mxi2|PRKM14|PRKM15|RK|SAPK2A|p38|p38ALPHA | 6 | 35995488 | 36079013 | Negative Regulator of Autophagy | mitogen-activated protein kinase 14 | EMBO J. 2010 Jan 6;29(1):27–40Coordinated regulation of autophagy by p38alpha MAPK through mAtg9 and p38IP. Webber JL, Tooze SA. | Autophagy core |
| *PPP2R2A* | ENSG00000221914 | B55A|B55ALPHA|PR52A|PR55A|PR55alpha | 8 | 26149007 | 26230196 | Negative Regulator of Autophagy | protein phosphatase 2, regulatory subunit B, alpha | J Biol Chem. 2016 May 13;291(20):10858–10866Regulation of Beclin 1 Protein Phosphorylation and Autophagy by Protein Phosphatase 2A (PP2A) and Death-associated Protein Kinase 3 (DAPK3). Fujiwara N, Usui T, Ohama T, Sato K. | Autophagy core |
| *CISD2* | ENSG00000145354 | ERIS|Miner1|NAF-1|WFS2|ZCD2 | 4 | 103790135 | 103810399 | Negative Regulator of Autophagy | CDGSH iron sulfur domain 2 | Cell Biol Int. 2015 Jul;39(7):816–823NAF-1 antagonizes starvation-induced autophagy through AMPK signaling pathway in cardiomyocytes. Du X, Xiao R, Xiao F, Chen Y, Hua F, Yu S, Xu G. | Autophagy core |
| *GOLGA2* | ENSG00000167110 | GM130 | 9 | 131018108 | 131038274 | Negative Regulator of Autophagy | golgin A2 | Mol Cell. 2015 Dec 17;60(6):899–913Activation of ULK Kinase and Autophagy by GABARAP Trafficking from the Centrosome Is Regulated by WAC and GM130. Joachim J, Jefferies HB, Razi M, Frith D, Snijders AP, Chakravarty P, Judith D, Tooze SA. | Autophagy core |
| *CYBB* | ENSG00000165168 | AMCBX2|CGD|GP91-1|GP91-PHOX|GP91PHOX|IMD34|NOX2|p91-PHOX | X | 37639264 | 37672714 | Negative Regulator of Autophagy | cytochrome b-245, beta polypeptide | Autophagy. 2016;12(4):705–706Enhanced autophagy as a potential mechanism for the improved physiological function by simvastatin in muscular dystrophy. Whitehead NP. | Autophagy core |
| *HSPA1A* | ENSG00000204389|ENSG00000215328|ENSG00000234475|ENSG00000235941|ENSG00000237724 | HEL-S-103|HSP70-1|HSP70-1A|HSP70-2|HSP70.1|HSP70.2|HSP70I|HSP72|HSPA1 | 6 | 31783291 | 31785723 | Negative Regulator of Autophagy | heat shock 70kDa protein 1A | J Biol Chem. 2013 May 24;288(21):14959–14972. Regulatory coordination between two major intracellular homeostatic systems: heat shock response and autophagy. Dokladny K, Zuhl MN, Mandell M, Bhattacharya D, Schneider S, Deretic V, Moseley PL. | Autophagy core |
| *UCP2* | ENSG00000175567 | BMIQ4|SLC25A8|UCPH | 11 | 73685712 | 73694352 | Negative Regulator of Autophagy | uncoupling protein 2 (mitochondrial, proton carrier) | Biochim Biophys Acta. 2013 Mar;1833(3):672–679UCP2 inhibition triggers ROS-dependent nuclear translocation of GAPDH and autophagic cell death in pancreatic adenocarcinoma cells. Dando I, Fiorini C, Pozza ED, Padroni C, Costanzo C, Palmieri M, Donadelli M. | Autophagy core |
| *C9orf72* | ENSG00000147894 | ALSFTD|DENND9|DENNL72|FTDALS|FTDALS1 | 9 | 27546544 | 27573864 | Negative Regulator of Autophagy | chromosome 9 open reading frame 72 | Autophagy. 2017 Jul 3;13(7):1254–1255Systemic deregulation of autophagy upon loss of ALS- and FTD-linked C9orf72. Ji YJ, Ugolino J, Brady NR, Hamacher-Brady A, Wang J. | Autophagy core |
| *HERC1* | ENSG00000103657 | MDFPMR|p532|p619 | 15 | 63900817 | 64126141 | Negative Regulator of Autophagy | HECT and RLD domain containing E3 ubiquitin protein ligase family member 1 | PLoS Genet. 2009 Dec;5(12):e1000784Progressive Purkinje cell degeneration in tambaleante mutant mice is a consequence of a missense mutation in HERC1 E3 ubiquitin ligase. Mashimo T, Hadjebi O, Amair-Pinedo F, Tsurumi T, Langa F, Serikawa T, Sotelo C, Guénet JL, Rosa JL. | Autophagy core |
| *TMEM173* | ENSG00000184584|ENSG00000288243 | MITA|STING1 | 5 | 138855119 | 138862520 | Negative Regulator of Autophagy | transmembrane protein 173 | Cell Signal. 2017 Jul;35:73–83MITA modulated autophagy flux promotes cell death in breast cancer cells. Bhatelia K, Singh K, Prajapati P, Sripada L, Roy M, Singh R. | Autophagy core |
| *MST1* | ENSG00000173531 | D3F15S2|DNF15S2|HGFL|MSP|NF15S2 | 3 | 49721380 | 49726934 | Negative Regulator of Autophagy | Macrophage Stimulating 1 | Nat Med. 2013 Nov;19(11):1478–1488Mst1 inhibits autophagy by promoting the interaction between Beclin1 and Bcl-2. Maejima Y, Kyoi S, Zhai P, Liu T, Li H, Ivessa A, Sciarretta S, Del Re DP, Zablocki DK, Hsu CP, Lim DS, Isobe M, Sadoshima J. | Autophagy core |
| *KIAA0226* | ENSG00000145016 | RUBICON | 3 | 197398264 | 197476598 | Negative Regulator of Autophagy | KIAA0226 | Biochim Biophys Acta. 2016 Apr;1861(4):269–284. Autophagy, lipophagy and lysosomal lipid storage disorders. Ward C, Martinez-Lopez N, Otten EG, Carroll B, Maetzel D, Singh R, Sarkar S, Korolchuk VI. | Autophagy core |
| *MAPK8* | ENSG00000107643 | JNK|JNK-46|JNK1|JNK1A2|JNK21B1/2|PRKM8|SAPK1|SAPK1c | 10 | 49514698 | 49647403 | Positive regulator of Autophagy | mitogen-activated protein kinase 8 | Biosci Rep. 2015 Apr 22;35(3):e00199MAPK/JNK signalling: a potential autophagy regulation pathway. Zhou YY, Li Y, Jiang WQ, Zhou LF. | Autophagy core |
| *MCOLN3* | ENSG00000055732 | TRP-ML3|TRPML3 | 1 | 85483765 | 85514182 | Positive regulator of Autophagy | mucolipin 3 | Cell Biol Int. 2015 Jul;39(7):816–823NAF-1 antagonizes starvation-induced autophagy through AMPK signaling pathway in cardiomyocytes. Du X, Xiao R, Xiao F, Chen Y, Hua F, Yu S, Xu G. | Autophagy core |
| *DRAM1* | ENSG00000136048 | DRAM | 12 | 102271129 | 102405908 | Positive regulator of Autophagy | DNA-damage regulated autophagy modulator 1 | PLoS One. 2013 May 17;8(5):e63245DRAM1 regulates autophagy flux through lysosomes. Zhang XD, Qi L, Wu JC, Qin ZH. | Autophagy core |
| *HDAC1* | ENSG00000116478 | GON-10|HD1|KDAC1|RPD3|RPD3L1 | 1 | 32757687 | 32799236 | Positive regulator of Autophagy | histone deacetylase 1 | Proc Natl Acad Sci U S A. 2012 Jan 31;109(5):1649–1654Histone deacetylases 1 and 2 regulate autophagy flux and skeletal muscle homeostasis in mice. Moresi V, Carrer M, Grueter CE, Rifki OF, Shelton JM, Richardson JA, Bassel-Duby R, Olson EN. | Autophagy core |
| *HDAC2* | ENSG00000196591 | HD2|KDAC2|RPD3|YAF1 | 6 | 114254192 | 114332472 | Positive regulator of Autophagy | histone deacetylase 2 | Proc Natl Acad Sci U S A. 2012 Jan 31;109(5):1649–1654Histone deacetylases 1 and 2 regulate autophagy flux and skeletal muscle homeostasis in mice. Moresi V, Carrer M, Grueter CE, Rifki OF, Shelton JM, Richardson JA, Bassel-Duby R, Olson EN. | Autophagy core |
| *TBK1* | ENSG00000183735 | FTDALS4|IIAE8|NAK|T2K | 12 | 64845660 | 64895888 | Positive regulator of Autophagy | TANK-binding kinase 1 | Proc Natl Acad Sci U S A. 2016 Apr 12;113(15):4039–4044Phosphorylation of OPTN by TBK1 enhances its binding to Ub chains and promotes selective autophagy of damaged mitochondria. Richter B, Sliter DA, Herhaus L, Stolz A, Wang C, Beli P, Zaffagnini G, Wild P, Martens S, Wagner SA, Youle RJ, Dikic I. | Autophagy core |
| *NOX4* | ENSG00000086991 | KOX|KOX-1|RENOX | 11 | 89057524 | 89322779 | Positive regulator of Autophagy | NADPH oxidase 4 | Autophagy. 2014 Apr;10(4):699–701NOX4 regulates autophagy during energy deprivation. Sciarretta S, Volpe M, Sadoshima J. | Autophagy core |
| *DAPK1* | ENSG00000196730 | DAPK|ROCO3 | 9 | 90112143 | 90323548 | Positive regulator of Autophagy | death-associated protein kinase 1 | Nat Rev Mol Cell Biol. 2018 Jun;19(6):349–364. Mechanism and medical implications of mammalian autophagy. Dikic I, Elazar Z. | Autophagy core |
| *DAPK2* | ENSG00000035664 | DRP-1|DRP1 | 15 | 64199235 | 64364232 | Positive regulator of Autophagy | death-associated protein kinase 2 | Cell Death Differ. 2015 Mar;22(3):465–475DAPK2 is a novel regulator of mTORC1 activity and autophagy. Ber Y, Shiloh R, Gilad Y, Degani N, Bialik S, Kimchi A. | Autophagy core |
| *CAST* | ENSG00000153113 | BS-17|PLACK | 5 | 95860971 | 96115299 | Positive regulator of Autophagy | calpastatin | Cell Death Differ. 2015 Mar;22(3):433–444Calpain inhibition mediates autophagy-dependent protection against polyglutamine toxicity. Menzies FM, Garcia-Arencibia M, Imarisio S, O'Sullivan NC, Ricketts T, Kent BA, Rao MV, Lam W, Green-Thompson ZW, Nixon RA, Saksida LM, Bussey TJ, O'Kane CJ, Rubinsztein DC. | Autophagy core |
| *PDCD6IP* | ENSG00000170248 | AIP1|ALIX|DRIP4|HP95 | 3 | 33839844 | 33911194 | Positive regulator of Autophagy | programmed cell death 6 interacting protein | Nat Cell Biol. 2015 Mar;17(3):300–310ATG12-ATG3 interacts with Alix to promote basal autophagic flux and late endosome function. Murrow L, Malhotra R, Debnath J. | Autophagy core |
| *PICALM* | ENSG00000073921 | CALM|CLTH|LAP | 11 | 85668727 | 85780924 | Positive regulator of Autophagy | phosphatidylinositol binding clathrin assembly protein | Nat Commun. 2014 Sep 22;5:4998. PICALM modulates autophagy activity and tau accumulation. Moreau K, Fleming A, Imarisio S, Lopez Ramirez A, Mercer JL, Jimenez-Sanchez M, Bento CF, Puri C, Zavodszky E, Siddiqi F, Lavau CP, Betton M, O'Kane CJ, Wechsler DS, Rubinsztein DC. | Autophagy core |
| *SMURF1* | ENSG00000198742|ENSG00000284126 | - | 7 | 98625061 | 98741723 | Positive regulator of Autophagy | SMAD specific E3 ubiquitin protein ligase 1 | Nature. 2011 Dec 1;480(7375):113–117Image-based genome-wide siRNA screen identifies selective autophagy factors. Orvedahl A, Sumpter R Jr, Xiao G, Ng A, Zou Z, Tang Y, Narimatsu M, Gilpin C, Sun Q, Roth M, Forst CV, Wrana JL, Zhang YE, Luby-Phelps K, Xavier RJ, Xie Y, Levine B. | Autophagy core |
| *SCOC* | ENSG00000153130 | HRIHFB2072|SCOCO|UNC-69 | 4 | 141178440 | 141306880 | Positive regulator of Autophagy | short coiled-coil protein | EMBO J. 2012 Apr 18;31(8):1931–1946Genome-wide siRNA screen reveals amino acid starvation-induced autophagy requires SCOC and WAC. McKnight NC, Jefferies HB, Alemu EA, Saunders RE, Howell M, Johansen T, Tooze SA. | Autophagy core |
| *WAC* | ENSG00000095787 | BM-016|DESSH|PRO1741|Wwp4 | 10 | 28821422 | 28912041 | Positive regulator of Autophagy | WW domain containing adaptor with coiled-coil | Mol Cell. 2015 Dec 17;60(6):899–913Activation of ULK Kinase and Autophagy by GABARAP Trafficking from the Centrosome Is Regulated by WAC and GM130. Joachim J, Jefferies HB, Razi M, Frith D, Snijders AP, Chakravarty P, Judith D, Tooze SA. | Autophagy core |
| *MYO6* | ENSG00000196586 | DFNA22|DFNB37 | 6 | 76458909 | 76629254 | Positive regulator of Autophagy | myosin VI | J Cell Sci. 2013 Jun 15;126(Pt 12):2561–2570Myosin VI and its cargo adaptors - linking endocytosis and autophagy. Tumbarello DA, Kendrick-Jones J, Buss F. | Autophagy core |
| *PIM2* | ENSG00000102096 | - | X | 48770459 | 48776301 | Positive regulator of Autophagy | pim-2 oncogene | J Cell Physiol. 2007 Oct;213(1):246–251PIM-2 is an independent regulator of chondrocyte survival and autophagy in the epiphyseal growth plate. Bohensky J, Shapiro IM, Leshinsky S, Watanabe H, Srinivas V. | Autophagy core |
| *DYNLL1* | ENSG00000088986 | DLC1|DLC8|DNCL1|DNCLC1|LC8|LC8a|PIN|hdlc1 | 12 | 120907653 | 120936296 | Positive regulator of Autophagy | Cytoplasmic dynein light chain | Proc Natl Acad Sci U S A. 2010 Jan 12;107(2):742–747Dynein light chain 1 is required for autophagy, protein clearance, and cell death in Drosophila. Batlevi Y, Martin DN, Pandey UB, Simon CR, Powers CM, Taylor JP, Baehrecke EH. | Autophagy core |
| *IRGM* | ENSG00000237693 | IFI1|IRGM1|LRG-47|LRG47 | 5 | 150226085 | 150280298 | Positive regulator of Autophagy | immunity-related GTPase family, M | Mol Cell. 2015 May 7;58(3):507–521IRGM governs the core autophagy machinery to conduct antimicrobial defense. Chauhan S, Mandell MA, Deretic V. | Autophagy core |
| *ABL2* | ENSG00000143322 | ABLL|ARG | 1 | 179068462 | 179198819 | Positive regulator of Autophagy | c-abl oncogene 2, non-receptor tyrosine kinase | J Biol Chem. 2008 Dec 19;283(51):35941–35953Abl kinases regulate autophagy by promoting the trafficking and function of lysosomal components. Yogalingam G, Pendergast AM. | Autophagy core |
| *ABL1* | ENSG00000097007 | ABL|BCR-ABL|CHDSKM|JTK7|bcr/abl|c-ABL|c-ABL1|p150|v-abl | 9 | 133589333 | 133763062 | Positive regulator of Autophagy | c-abl oncogene 1, non-receptor tyrosine kinase | J Biol Chem. 2008 Dec 19;283(51):35941–35953Abl kinases regulate autophagy by promoting the trafficking and function of lysosomal components. Yogalingam G, Pendergast AM. | Autophagy core |
| *TMEM59* | ENSG00000116209 | C1orf8|DCF1|HSPC001|PRO195|UNQ169 | 1 | 54497347 | 54519177 | Positive regulator of Autophagy | transmembrane protein 59 | EMBO J. 2013 Feb 20;32(4):566–582TMEM59 defines a novel ATG16L1-binding motif that promotes local activation of LC3. Boada-Romero E, Letek M, Fleischer A, Pallauf K, Ramón-Barros C, Pimentel-Muiños FX. | Autophagy core |
| *EI24* | ENSG00000149547 | EPG4|PIG8|TP53I8 | 11 | 125439112 | 125454575 | Positive regulator of Autophagy | etoposide induced 2.4 | J Biol Chem. 2012 Dec 7;287(50):42053–42063The p53-induced gene Ei24 is an essential component of the basal autophagy pathway. Zhao YG, Zhao H, Miao L, Wang L, Sun F, Zhang H. | Autophagy core |
| *MTDH* | ENSG00000147649 | 3D3|AEG-1|AEG1|LYRIC|LYRIC/3D3 | 8 | 98656407 | 98740998 | Positive regulator of Autophagy | metadherin | Tumour Biol. 2013 Aug;34(4):2433–2440Metadherin confers chemoresistance of cervical cancer cells by inducing autophagy and activating ERK/NF-κB pathway. Zhang J, Zhang Y, Liu S, Zhang Q, Wang Y, Tong L, Chen X, Ji Y, Shang Q, Xu B, Chu M, Wei L. | Autophagy core |
| *EVA1A* | ENSG00000115363 | FAM176A|TMEM166 | 2 | 75696428 | 75796848 | Positive regulator of Autophagy | eva-1 homolog A (C. elegans) | Stem Cell Reports. 2016 Mar 8;6(3):396–410EVA1A/TMEM166 Regulates Embryonic Neurogenesis by Autophagy. Li M, Lu G, Hu J, Shen X, Ju J, Gao Y, Qu L, Xia Y, Chen Y, Bai Y. | Autophagy core |
| *SUPT20H* | ENSG00000102710 | C13|C13orf19|FAM48A|FP757|P38IP|SPT20 | 13 | 37583449 | 37633850 | Positive regulator of Autophagy | suppressor of Ty 20 homolog (S. cerevisiae) | EMBO J. 2010 Jan 6;29(1):27–40Coordinated regulation of autophagy by p38alpha MAPK through mAtg9 and p38IP. Webber JL, Tooze SA. | Autophagy core |
| *DAPK3* | ENSG00000167657 | DLK|ZIP|ZIPK | 19 | 3958451 | 3971121 | Positive regulator of Autophagy | death-associated protein kinase 3 | J Biol Chem. 2016 May 13;291(20):10858–10866Regulation of Beclin 1 Protein Phosphorylation and Autophagy by Protein Phosphatase 2A (PP2A) and Death-associated Protein Kinase 3 (DAPK3). Fujiwara N, Usui T, Ohama T, Sato K. | Autophagy core |
| *BAG3* | ENSG00000151929 | BAG-3|BIS|CAIR-1|MFM6 | 10 | 121410882 | 121437331 | Positive regulator of Autophagy | BCL2-associated athanogene 3 | Autophagy. 2011 Jul;7(7):795–798BAG3 and friends: co-chaperones in selective autophagy during aging and disease. Behl C. | Autophagy core |
| *TMEM74* | ENSG00000164841 | NET36 | 8 | 109619079 | 109799844 | Positive regulator of Autophagy | transmembrane protein 74 | Biochem Biophys Res Commun. 2008 May 2;369(2):622–629. TMEM74, a lysosome and autophagosome protein, regulates autophagy. Yu C, Wang L, Lv B, Lu Y, Zeng L, Chen Y, Ma D, Shi T, Wang L. | Autophagy core |
| *TM9SF1* | ENSG00000100926|ENSG00000285465 | HMP70|MP70 | 14 | 24658349 | 24682679 | Positive regulator of Autophagy | transmembrane 9 superfamily member 1 | Autophagy. 2009 Jan;5(1):52–60High-throughput functional screening for autophagy-related genes and identification of TM9SF1 as an autophagosome-inducing gene. He P, Peng Z, Luo Y, Wang L, Yu P, Deng W, An Y, Shi T, Ma D. | Autophagy core |
| *ZFYVE1* | ENSG00000165861 | DFCP1|PPP1R172|SR3|TAFF1|ZNFN2A1 | 14 | 73436159 | 73493920 | Positive regulator of Autophagy | zinc finger, FYVE domain containing 1 | Semin Cancer Biol. 2013 Oct;23(5):301–309Autophagosome formation--the role of ULK1 and Beclin1-PI3KC3 complexes in setting the stage. Wirth M, Joachim J, Tooze SA. | Autophagy core |
| *KIAA1324* | ENSG00000116299 | EIG121 | 1 | 109656301 | 109749401 | Positive regulator of Autophagy | KIAA1324 | Cell Death Dis. 2010;1(4):e32The novel estrogen-induced gene EIG121 regulates autophagy and promotes cell survival under stress. Deng L, Feng J, Broaddus RR. | Autophagy core |
| *HDAC10* | ENSG00000100429 | HD10 | 22 | 50683612 | 50689834 | Positive regulator of Autophagy | histone deacetylase 10 | Proc Natl Acad Sci U S A. 2013 Jul 9;110(28):E2592–E2601Histone deacetylase 10 promotes autophagy-mediated cell survival. Oehme I, Linke JP, Böck BC, Milde T, Lodrini M, Hartenstein B, Wiegand I, Eckert C, Roth W, Kool M, Kaden S, Gröne HJ, Schulte JH, Lindner S, Hamacher-Brady A, Brady NR, Deubzer HE, Witt O. | Autophagy core |
| *SNAP29* | ENSG00000099940 | CEDNIK|SNAP-29 | 22 | 21213271 | 21245506 | Positive regulator of Autophagy | synaptosomal-associated protein, 29kDa | Autophagy. 2014;10(12):2251–2268Multiple functions of the SNARE protein Snap29 in autophagy, endocytic, and exocytic trafficking during epithelial formation in Drosophila. Morelli E, Ginefra P, Mastrodonato V, Beznoussenko GV, Rusten TE, Bilder D, Stenmark H, Mironov AA, Vaccari T. Nature. 2015 Apr 23;520(7548):563–566ATG14 promotes membrane tethering and fusion of autophagosomes to endolysosomes. Diao J, Liu R, Rong Y, Zhao M, Zhang J, Lai Y, Zhou Q, Wilz LM, Li J, Vivona S, Pfuetzner RA, Brunger AT, Zhong Q. | Autophagy core |
| *PXN* | ENSG00000089159 | - | 12 | 120648250 | 120703574 | Positive regulator of Autophagy | paxillin | Oncogene. 2017 Mar 23;36(12):1619–1630Autophagy in cancer metastasis. Mowers EE, Sharifi MN, Macleod KF. | Autophagy core |
| *TGM2* | ENSG00000198959 | TG(C)|TGC | 20 | 36756863 | 36794980 | Positive regulator of Autophagy | transglutaminase 2 | Autophagy. 2009 Nov;5(8):1145–1154. Transglutaminase 2 is involved in autophagosome maturation. D'Eletto M, Farrace MG, Falasca L, Reali V, Oliverio S, Melino G, Griffin M, Fimia GM, Piacentini M. | Autophagy core |
| *STUB1* | ENSG00000103266 | CHIP|HSPABP2|NY-CO-7|SCA48|SCAR16|SDCCAG7|UBOX1 | 16 | 730224 | 732870 | Positive regulator of Autophagy | STIP1 homology and U-box containing protein 1, E3 ubiquitin protein ligase | EMBO J. 2017 Sep 1;36(17):2544–2552STUB1 regulates TFEB-induced autophagy-lysosome pathway. Sha Y, Rao L, Settembre C, Ballabio A, Eissa NT. | Autophagy core |
| *PMAIP1* | ENSG00000141682 | APR|NOXA | 18 | 57567180 | 57571538 | Positive regulator of Autophagy | Phorbol-12-Myristate-13-Acetate-Induced Protein 1 | FEBS J. 2015 Mar;282(6):1006–1016BH3-only proteins: a 20-year stock-take. Doerflinger M, Glab JA, Puthalakath H. | Autophagy core |
| *PCM1* | ENSG00000078674 | PTC4|RET/PCM-1 | 8 | 17780349 | 17885478 | Positive regulator of Autophagy | Pericentriolar Material 1 | Curr Biol. 2017 Jul 24;27(14):2123–2136.e7Centriolar Satellites Control GABARAP Ubiquitination and GABARAP-Mediated Autophagy. Joachim J, Razi M, Judith D, Wirth M, Calamita E, Encheva V, Dynlacht BD, Snijders AP, O'Reilly N, Jefferies HBJ, Tooze SA. | Autophagy core |
| *RAB3GAP1* | ENSG00000115839 | P130|RAB3GAP|RAB3GAP130|WARBM1 | 2 | 135809835 | 135933964 | Positive regulator of Autophagy | RAB3 GTPase Activating Protein Catalytic Subunit 1 | Autophagy. 2014;10(12):2297–2309RAB3GAP1 and RAB3GAP2 modulate basal and rapamycin-induced autophagy. Spang N, Feldmann A, Huesmann H, Bekbulat F, Schmitt V, Hiebel C, Koziollek-Drechsler I, Clement AM, Moosmann B, Jung J, Behrends C, Dikic I, Kern A, Behl C. | Autophagy core |
| *RAB18* | ENSG00000099246 | RAB18LI1|WARBM3 | 10 | 27793197 | 27831143 | Positive regulator of Autophagy | RAB18, Member RAS Oncogene Family | Biochem Biophys Res Commun. 2017 May 6;486(3):738–743The RAB GTPase RAB18 modulates macroautophagy and proteostasis. Feldmann A, Bekbulat F, Huesmann H, Ulbrich S, Tatzelt J, Behl C, Kern A. | Autophagy core |
| *CLN3* | ENSG00000188603 | BTN1|BTS|JNCL | 16 | 28477983 | 28506896 | Its mutation leads to Autophagy defects | ceroid-lipofuscinosis, neuronal 3 | Neurology. 2014 Jun 10;82(23):2072–2076Novel CLN3 mutation causing autophagic vacuolar myopathy. Cortese A, Tucci A, Piccolo G, Galimberti CA, Fratta P, Marchioni E, Grampa G, Cereda C, Grieco G, Ricca I, Pittman A, Ciscato P, Napoli L, Lucchini V, Ripolone M, Violano R, Fagiolari G, Mole SE, Hardy J, Moglia A, Moggio M. | Autophagy core |
| *CFTR* | ENSG00000001626 | ABC35|ABCC7|CF|CFTR/MRP|MRP7|TNR-CFTR|dJ760C5.1 | 7 | 117105838 | 117356025 | Its mutation leads to Autophagy defects | cystic fibrosis transmembrane conductance regulator (ATP-binding cassette sub-family C, member 7) | Nat Cell Biol. 2010 Sep;12(9):863–875Defective CFTR induces aggresome formation and lung inflammation in cystic fibrosis through ROS-mediated autophagy inhibition. Luciani A, Villella VR, Esposito S, Brunetti-Pierri N, Medina D, Settembre C, Gavina M, Pulze L, Giardino I, Pettoello-Mantovani M, D'Apolito M, Guido S, Masliah E, Spencer B, Quaratino S, Raia V, Ballabio A, Maiuri L. | Autophagy core |
| *TP53INP1* | ENSG00000164938 | SIP|TP53DINP1|TP53INP1A|TP53INP1B|Teap|p53DINP1 | 8 | 95938200 | 95961639 | Autophagy-dependent cell death | tumor protein p53 inducible nuclear protein 1 | Cell Death Differ. 2012 Sep;19(9):1525–1535TP53INP1, a tumor suppressor, interacts with LC3 and ATG8-family proteins through the LC3-interacting region (LIR) and promotes autophagy-dependent cell death. Seillier M, Peuget S, Gayet O, Gauthier C, N'Guessan P, Monte M, Carrier A, Iovanna JL, Dusetti NJ. | Autophagy core |
| *TRIM13* | ENSG00000204977 | CAR|DLEU5|LEU5|RFP2|RNF77 | 13 | 50570024 | 50594617 | Autophagy-dependent cell death | tripartite motif containing 13 | Biochim Biophys Acta. 2013 Dec;1833(12):3134–3144TRIM13 regulates caspase-8 ubiquitination, translocation to autophagosomes and activation during ER stress induced cell death. Tomar D, Prajapati P, Sripada L, Singh K, Singh R, Singh AK, Singh R. | Autophagy core |
| *RAB11A* | ENSG00000103769 | YL8 | 15 | 66018392 | 66184329 | Rab proteins involved in autophagosome formation | RAB11A, member RAS oncogene family | Cell Death Differ. 2014 Mar;21(3):348–358Regulation of autophagy by the Rab GTPase network. Ao X, Zou L, Wu Y. | Autophagy core |
| *RAB23* | ENSG00000112210 | HSPC137 | 6 | 57053607 | 57087078 | Rab proteins involved in autophagosome formation | RAB23, member RAS oncogene family | Cell Death Differ. 2014 Mar;21(3):348–358Regulation of autophagy by the Rab GTPase network. Ao X, Zou L, Wu Y. | Autophagy core |
| *RAB1A* | ENSG00000138069 | RAB1|YPT1 | 2 | 65297835 | 65357240 | Rab proteins involved in autophagosome formation | RAB1A, member RAS oncogene family | Cell Death Differ. 2014 Mar;21(3):348–358Regulation of autophagy by the Rab GTPase network. Ao X, Zou L, Wu Y. | Autophagy core |
| *RAB5A* | ENSG00000144566 | RAB5 | 3 | 19988571 | 20026667 | Rab proteins involved in autophagosome formation | RAB5A, member RAS oncogene family | Cell Death Differ. 2014 Mar;21(3):348–358Regulation of autophagy by the Rab GTPase network. Ao X, Zou L, Wu Y. | Autophagy core |
| *RAB8B* | ENSG00000166128 | - | 15 | 63481668 | 63559981 | Rab proteins involved in autophagosome formation | RAB8B, member RAS oncogene family | Cell Death Differ. 2014 Mar;21(3):348–358Regulation of autophagy by the Rab GTPase network. Ao X, Zou L, Wu Y. | Autophagy core |
| *RAB1B* | ENSG00000174903 | - | 11 | 66036004 | 66044963 | Rab proteins involved in autophagosome formation | RAB1B, member RAS oncogene family | Cell Death Differ. 2014 Mar;21(3):348–358Regulation of autophagy by the Rab GTPase network. Ao X, Zou L, Wu Y. | Autophagy core |
| *RAB7A* | ENSG00000075785 | CMT2B|PRO2706|RAB7 | 3 | 128444965 | 128533639 | Rab proteins involved in autophagosome formation | RAB7A, member RAS oncogene family | Cell Death Differ. 2014 Mar;21(3):348–358Regulation of autophagy by the Rab GTPase network. Ao X, Zou L, Wu Y. | Autophagy core |
| *RAB5B* | ENSG00000111540 | - | 12 | 56367697 | 56388490 | Rab proteins involved in autophagosome formation | RAB5B, member RAS oncogene family | Cell Death Differ. 2014 Mar;21(3):348–358Regulation of autophagy by the Rab GTPase network. Ao X, Zou L, Wu Y. | Autophagy core |
| *RAB5C* | ENSG00000108774 | L1880|RAB5CL|RAB5L|RABL | 17 | 40276994 | 40307035 | Rab proteins involved in autophagosome formation | RAB5C, member RAS oncogene family | Cell Death Differ. 2014 Mar;21(3):348–358Regulation of autophagy by the Rab GTPase network. Ao X, Zou L, Wu Y. | Autophagy core |
| *RAB32* | ENSG00000118508 | - | 6 | 146864829 | 146876101 | Rab proteins involved in autophagosome formation | RAB32, member RAS oncogene family | Cell Death Differ. 2014 Mar;21(3):348–358Regulation of autophagy by the Rab GTPase network. Ao X, Zou L, Wu Y. | Autophagy core |
| *RAB24* | ENSG00000169228 | - | 5 | 176728199 | 176730745 | Rab proteins involved in autophagosome formation | RAB24, member RAS oncogene family | Cell Death Differ. 2014 Mar;21(3):348–358Regulation of autophagy by the Rab GTPase network. Ao X, Zou L, Wu Y. | Autophagy core |
| *RAB8A* | ENSG00000167461 | MEL|RAB8 | 19 | 16222439 | 16245044 | Rab proteins involved in autophagosome formation | RAB8A, member RAS oncogene family | Cell Death Differ. 2014 Mar;21(3):348–358Regulation of autophagy by the Rab GTPase network. Ao X, Zou L, Wu Y. | Autophagy core |
| *RAB11B* | ENSG00000185236 | H-YPT3|NDAGSCW | 19 | 8454865 | 8469318 | Rab proteins involved in autophagosome formation | RAB11B, member RAS oncogene family | Cell Death Differ. 2014 Mar;21(3):348–358Regulation of autophagy by the Rab GTPase network. Ao X, Zou L, Wu Y. | Autophagy core |
| *RAB33B* | ENSG00000172007 | SMC2 | 4 | 140374386 | 140397763 | Rab proteins involved in autophagosome formation | RAB33B, member RAS oncogene family | Cell Death Differ. 2014 Mar;21(3):348–358Regulation of autophagy by the Rab GTPase network. Ao X, Zou L, Wu Y. | Autophagy core |
| *RAB1C* | ENSG00000233111 | RAB1C | 9 | 37636685 | 37637290 | Rab proteins involved in autophagosome formation | RAB1C, member RAS oncogene family pseudogene | Cell Death Differ. 2014 Mar;21(3):348–358Regulation of autophagy by the Rab GTPase network. Ao X, Zou L, Wu Y. | Autophagy core |
| *RAB9A* | ENSG00000123595 | RAB9 | X | 13707244 | 13728625 | Rab proteins involved in autophagosome formation | RAB9A, member RAS oncogene family | Cell Death Differ. 2014 Mar;21(3):348–358Regulation of autophagy by the Rab GTPase network. Ao X, Zou L, Wu Y. | Autophagy core |
| *RAB25* | ENSG00000132698 | CATX-8|RAB11C | 1 | 156030951 | 156040295 | Rab proteins involved in autophagosome formation | RAB25, member RAS oncogene family | Cell Death Differ. 2014 Mar;21(3):348–358Regulation of autophagy by the Rab GTPase network. Ao X, Zou L, Wu Y. | Autophagy core |
| *RAB4A* | ENSG00000168118 | HRES-1|HRES-1/RAB4|HRES1|RAB4 | 1 | 229406822 | 229441641 | Rab proteins involved in autophagosome formation | RAB4A, member RAS oncogene family | PLoS One. 2014 Jan 3;9(1):e84392HRES-1/Rab4 promotes the formation of LC3(+) autophagosomes and the accumulation of mitochondria during autophagy. Talaber G, Miklossy G, Oaks Z, Liu Y, Tooze SA, Chudakov DM, Banki K, Perl A. | Autophagy core |
| *RAB4B* | ENSG00000167578 | - | 19 | 41284121 | 41302847 | Rab proteins involved in autophagosome formation | RAB4B, member RAS oncogene family | PLoS One. 2014 Jan 3;9(1):e84392HRES-1/Rab4 promotes the formation of LC3(+) autophagosomes and the accumulation of mitochondria during autophagy. Talaber G, Miklossy G, Oaks Z, Liu Y, Tooze SA, Chudakov DM, Banki K, Perl A. | Autophagy core |
| *USP25* | ENSG00000155313 | USP21 | 21 | 17102344 | 17252377 | Ubiquitin and Autophagy | ubiquitin specific peptidase 25 | Proc Natl Acad Sci U S A. 2015 Sep 8;112(36):11324–11329Induction of USP25 by viral infection promotes innate antiviral responses by mediating the stabilization of TRAF3 and TRAF6. Lin D, Zhang M, Zhang MX, Ren Y, Jin J, Zhao Q, Pan Z, Wu M, Shu HB, Dong C, Zhong B. | Autophagy core |
| *USP16* | ENSG00000156256 | UBP-M|UBPM | 21 | 30396950 | 30426809 | Ubiquitin and Autophagy | ubiquitin specific peptidase 16 | Biochem J. 2015 Jan 1;465(1):1–26DUBs, the regulation of cell identity and disease. Heideker J, Wertz IE. | Autophagy core |
| *U2AF1* | ENSG00000160201 | FP793|RN|RNU2AF1|U2AF35|U2AFBP | 21 | 44513066 | 44527697 | Ubiquitin and Autophagy | U2 small nuclear RNA auxiliary factor 1 | Mol Cell. 2016 May 19;62(4):473–474Defective Autophagy Initiates Malignant Transformation. Galluzzi L, Bravo-San Pedro JM, Kroemer G. | Autophagy core |
| *USP8* | ENSG00000138592 | HumORF8|PITA4|SPG59|UBPY | 15 | 50716577 | 50793280 | Ubiquitin and Autophagy | ubiquitin specific peptidase 8 | EMBO J. 2014 Nov 3;33(21):2473–2491USP8 regulates mitophagy by removing K6-linked ubiquitin conjugates from parkin. Durcan TM, Tang MY, Pérusse JR, Dashti EA, Aguileta MA, McLelland GL, Gros P, Shaler TA, Faubert D, Coulombe B, Fon EA. | Autophagy core |
| *USP7* | ENSG00000187555 | HAFOUS|HAUSP|TEF1 | 16 | 8985951 | 9058371 | Ubiquitin and Autophagy | ubiquitin specific peptidase 7 (herpes virus-associated) | Nature. 2010 Jul 1;466(7302):68–76Network organization of the human autophagy system. Behrends C, Sowa ME, Gygi SP, Harper JW. | Autophagy core |
| *USP11* | ENSG00000102226 | UHX1 | X | 47092089 | 47107727 | Ubiquitin and Autophagy | ubiquitin specific peptidase 11 | Nature. 2010 Jul 1;466(7302):68–76Network organization of the human autophagy system. Behrends C, Sowa ME, Gygi SP, Harper JW. | Autophagy core |
| *USP10* | ENSG00000103194 | UBPO | 16 | 84733584 | 84813528 | Ubiquitin and Autophagy | ubiquitin specific peptidase 10 | Nature. 2010 Jul 1;466(7302):68–76Network organization of the human autophagy system. Behrends C, Sowa ME, Gygi SP, Harper JW. | Autophagy core |
| *PIK3AP1* | ENSG00000155629 | BCAP | 10 | 98353069 | 98480271 | Candidate kinases required for basal Autophagy | phosphoinositide-3-kinase adaptor protein 1 | Autophagy. 2009 Oct;5(7):1018–1025Identification of novel autophagy regulators by a luciferase-based assay for the kinetics of autophagic flux. Farkas T, Høyer-Hansen M, Jäättelä M. | Autophagy core |
| *TSKS* | ENSG00000126467 | PPP1R161|STK22S1|TSKS1|TSSKS | 19 | 50243010 | 50266587 | Candidate kinases required for basal Autophagy | testis-specific serine kinase substrate | Autophagy. 2009 Oct;5(7):1018–1025Identification of novel autophagy regulators by a luciferase-based assay for the kinetics of autophagic flux. Farkas T, Høyer-Hansen M, Jäättelä M. | Autophagy core |
| *NRBP2* | ENSG00000185189 | TRG16|pp9320 | 8 | 144915764 | 144924200 | Candidate kinases required for basal Autophagy | nuclear receptor binding protein 2 | Autophagy. 2009 Oct;5(7):1018–1025Identification of novel autophagy regulators by a luciferase-based assay for the kinetics of autophagic flux. Farkas T, Høyer-Hansen M, Jäättelä M. | Autophagy core |
| *NEK9* | ENSG00000119638 | APUG|LCCS10|NC|NERCC|NERCC1 | 14 | 75548822 | 75594047 | Candidate kinases required for basal Autophagy | NIMA-related kinase 9 | Autophagy. 2009 Oct;5(7):1018–1025Identification of novel autophagy regulators by a luciferase-based assay for the kinetics of autophagic flux. Farkas T, Høyer-Hansen M, Jäättelä M. | Autophagy core |
| *PGK2* | ENSG00000170950 | HEL-S-272|PGKB|PGKPS|dJ417L20.2 | 6 | 49753366 | 49755053 | Candidate kinases required for basal Autophagy | phosphoglycerate kinase 2 | Autophagy. 2009 Oct;5(7):1018–1025Identification of novel autophagy regulators by a luciferase-based assay for the kinetics of autophagic flux. Farkas T, Høyer-Hansen M, Jäättelä M. | Autophagy core |
| *NME4* | ENSG00000103202 | NDPK-D|NM23H4|nm23-H4 | 16 | 446725 | 460367 | Candidate kinases required for basal Autophagy | NME/NM23 nucleoside diphosphate kinase 4 | Autophagy. 2009 Oct;5(7):1018–1025Identification of novel autophagy regulators by a luciferase-based assay for the kinetics of autophagic flux. Farkas T, Høyer-Hansen M, Jäättelä M. | Autophagy core |
| *PIK3R4* | ENSG00000196455 | VPS15|p150 | 3 | 130397779 | 130465673 | Candidate kinases required for basal Autophagy | phosphoinositide-3-kinase, regulatory subunit 4 | Autophagy. 2009 Oct;5(7):1018–1025Identification of novel autophagy regulators by a luciferase-based assay for the kinetics of autophagic flux. Farkas T, Høyer-Hansen M, Jäättelä M. | Autophagy core |
| *MOS* | ENSG00000172680 | MSV | 8 | 57025501 | 57026541 | Candidate kinases required for basal Autophagy | v-mos Moloney murine sarcoma viral oncogene homolog | Autophagy. 2009 Oct;5(7):1018–1025Identification of novel autophagy regulators by a luciferase-based assay for the kinetics of autophagic flux. Farkas T, Høyer-Hansen M, Jäättelä M. | Autophagy core |
| *IKBKB* | ENSG00000104365 | IKK-beta|IKK2|IKKB|IMD15|IMD15A|IMD15B|NFKBIKB | 8 | 42128820 | 42189973 | Candidate kinases required for basal Autophagy | inhibitor of kappa light polypeptide gene enhancer in B-cells, kinase beta | Autophagy. 2009 Oct;5(7):1018–1025Identification of novel autophagy regulators by a luciferase-based assay for the kinetics of autophagic flux. Farkas T, Høyer-Hansen M, Jäättelä M. | Autophagy core |
| *TPD52L3* | ENSG00000170777 | D55|NYDSP25|TPD55|hD55 | 9 | 6328349 | 6331900 | Candidate kinases required for basal Autophagy | tumor protein D52-like 3 | Autophagy. 2009 Oct;5(7):1018–1025Identification of novel autophagy regulators by a luciferase-based assay for the kinetics of autophagic flux. Farkas T, Høyer-Hansen M, Jäättelä M. | Autophagy core |
| *LIMK1* | ENSG00000106683 | LIMK|LIMK-1 | 7 | 73497263 | 73536855 | Candidate kinases required for basal Autophagy | LIM domain kinase 1 | Autophagy. 2009 Oct;5(7):1018–1025Identification of novel autophagy regulators by a luciferase-based assay for the kinetics of autophagic flux. Farkas T, Høyer-Hansen M, Jäättelä M. | Autophagy core |
| *GLYCTK* | ENSG00000168237 | HBEBP2|HBEBP4|HBeAgBP4A | 3 | 52321105 | 52329272 | Candidate kinases required for basal Autophagy | glycerate kinase | Autophagy. 2009 Oct;5(7):1018–1025Identification of novel autophagy regulators by a luciferase-based assay for the kinetics of autophagic flux. Farkas T, Høyer-Hansen M, Jäättelä M. | Autophagy core |
| *PRKACA* | ENSG00000072062|ENSG00000288516 | PKACA|PPNAD4 | 19 | 14202500 | 14228896 | Candidate kinases required for basal Autophagy | protein kinase, cAMP-dependent, catalytic, alpha | Autophagy. 2009 Oct;5(7):1018–1025Identification of novel autophagy regulators by a luciferase-based assay for the kinetics of autophagic flux. Farkas T, Høyer-Hansen M, Jäättelä M. | Autophagy core |
| *ALPK2* | ENSG00000198796 | HAK | 18 | 56148479 | 56296189 | Candidate kinases required for basal Autophagy | alpha-kinase 2 | Autophagy. 2009 Oct;5(7):1018–1025Identification of novel autophagy regulators by a luciferase-based assay for the kinetics of autophagic flux. Farkas T, Høyer-Hansen M, Jäättelä M. | Autophagy core |
| *ACVR2B* | ENSG00000114739 | ACTRIIB|ActR-IIB|HTX4 | 3 | 38495342 | 38534633 | Candidate kinases required for basal Autophagy | activin A receptor, type IIB | Autophagy. 2009 Oct;5(7):1018–1025Identification of novel autophagy regulators by a luciferase-based assay for the kinetics of autophagic flux. Farkas T, Høyer-Hansen M, Jäättelä M. | Autophagy core |
| *MARK4* | ENSG00000007047 | MARK4L|MARK4S|MARKL1|MARKL1L|PAR-1D | 19 | 45582546 | 45808541 | Candidate kinases required for basal Autophagy | MAP/microtubule affinity-regulating kinase 4 | Autophagy. 2009 Oct;5(7):1018–1025Identification of novel autophagy regulators by a luciferase-based assay for the kinetics of autophagic flux. Farkas T, Høyer-Hansen M, Jäättelä M. | Autophagy core |
| *MAP3K1* | ENSG00000095015 | MAPKKK1|MEKK|MEKK 1|MEKK1|SRXY6 | 5 | 56111401 | 56191979 | Candidate kinases required for basal Autophagy | mitogen-activated protein kinase kinase kinase 1, E3 ubiquitin protein ligase | Autophagy. 2009 Oct;5(7):1018–1025Identification of novel autophagy regulators by a luciferase-based assay for the kinetics of autophagic flux. Farkas T, Høyer-Hansen M, Jäättelä M. | Autophagy core |
| *NUAK1* | ENSG00000074590 | ARK5 | 12 | 106457118 | 106533811 | Candidate kinases required for basal Autophagy | NUAK family, SNF1-like kinase, 1 | Autophagy. 2009 Oct;5(7):1018–1025Identification of novel autophagy regulators by a luciferase-based assay for the kinetics of autophagic flux. Farkas T, Høyer-Hansen M, Jäättelä M. | Autophagy core |
| *LY6G5B* | ENSG00000239285|ENSG00000239497|ENSG00000240053|ENSG00000240433|ENSG00000241132|ENSG00000241713|ENSG00000244672 | C6orf19|G5b | 6 | 31637944 | 31641553 | Candidate kinases required for basal Autophagy | lymphocyte antigen 6 complex, locus G5B | Autophagy. 2009 Oct;5(7):1018–1025Identification of novel autophagy regulators by a luciferase-based assay for the kinetics of autophagic flux. Farkas T, Høyer-Hansen M, Jäättelä M. | Autophagy core |
| *PRDX5* | ENSG00000126432 | ACR1|AOEB166|B166|HEL-S-55|PLP|PMP20|PRDX6|PRXV|SBBI10|prx-V | 11 | 64085560 | 64089283 | Candidate kinases required for basal Autophagy | peroxiredoxin 5 | Autophagy. 2009 Oct;5(7):1018–1025Identification of novel autophagy regulators by a luciferase-based assay for the kinetics of autophagic flux. Farkas T, Høyer-Hansen M, Jäättelä M. | Autophagy core |
| *PRKDC* | ENSG00000253729 | DNA-PKC|DNA-PKcs|DNAPK|DNAPKc|DNPK1|HYRC|HYRC1|IMD26|XRCC7|p350 | 8 | 48685669 | 48872743 | Candidate kinases required for basal Autophagy | protein kinase, DNA-activated, catalytic polypeptide | Autophagy. 2009 Oct;5(7):1018–1025Identification of novel autophagy regulators by a luciferase-based assay for the kinetics of autophagic flux. Farkas T, Høyer-Hansen M, Jäättelä M. | Autophagy core |
| *AK7* | ENSG00000140057 | AK 7|CFAP75|FAP75|SPGF27 | 14 | 96858448 | 96955764 | Candidate kinases required for basal Autophagy | adenylate kinase 7 | Autophagy. 2009 Oct;5(7):1018–1025Identification of novel autophagy regulators by a luciferase-based assay for the kinetics of autophagic flux. Farkas T, Høyer-Hansen M, Jäättelä M. | Autophagy core |
| *PRKCE* | ENSG00000171132 | PKCE|nPKC-epsilon | 2 | 45878484 | 46415129 | Candidate kinases required for basal Autophagy | protein kinase C, epsilon | Autophagy. 2009 Oct;5(7):1018–1025Identification of novel autophagy regulators by a luciferase-based assay for the kinetics of autophagic flux. Farkas T, Høyer-Hansen M, Jäättelä M. | Autophagy core |
| *CDC42SE2* | ENSG00000158985 | SPEC2 | 5 | 130581186 | 130734140 | Candidate kinases required for basal Autophagy | CDC42 small effector 2 | Autophagy. 2009 Oct;5(7):1018–1025Identification of novel autophagy regulators by a luciferase-based assay for the kinetics of autophagic flux. Farkas T, Høyer-Hansen M, Jäättelä M. | Autophagy core |
| *MAPK4* | ENSG00000141639 | ERK-4|ERK4|PRKM4|p63-MAPK|p63MAPK | 18 | 48086448 | 48258194 | Candidate kinases required for basal Autophagy | mitogen-activated protein kinase 4 | Autophagy. 2009 Oct;5(7):1018–1025Identification of novel autophagy regulators by a luciferase-based assay for the kinetics of autophagic flux. Farkas T, Høyer-Hansen M, Jäättelä M. | Autophagy core |
| *TIE1* | ENSG00000066056 | JTK14|TIE | 1 | 43766664 | 43788779 | Candidate kinases required for basal Autophagy | tyrosine kinase with immunoglobulin-like and EGF-like domains 1 | Autophagy. 2009 Oct;5(7):1018–1025Identification of novel autophagy regulators by a luciferase-based assay for the kinetics of autophagic flux. Farkas T, Høyer-Hansen M, Jäättelä M. | Autophagy core |
| *SKAP1* | ENSG00000141293 | HEL-S-81p|SCAP1|SKAP55 | 17 | 46210802 | 46507637 | Candidate kinases required for basal Autophagy | src kinase associated phosphoprotein 1 | Autophagy. 2009 Oct;5(7):1018–1025Identification of novel autophagy regulators by a luciferase-based assay for the kinetics of autophagic flux. Farkas T, Høyer-Hansen M, Jäättelä M. | Autophagy core |
| *CLK3* | ENSG00000179335 | PHCLK3|PHCLK3/152 | 15 | 74890841 | 74932057 | Candidate kinases required for basal Autophagy | CDC-like kinase 3 | Autophagy. 2009 Oct;5(7):1018–1025Identification of novel autophagy regulators by a luciferase-based assay for the kinetics of autophagic flux. Farkas T, Høyer-Hansen M, Jäättelä M. | Autophagy core |
| *DGKB* | ENSG00000136267 | DAGK2|DGK|DGK-BETA | 7 | 14184674 | 15014402 | Candidate kinases required for basal Autophagy | diacylglycerol kinase, beta 90kDa | Autophagy. 2009 Oct;5(7):1018–1025Identification of novel autophagy regulators by a luciferase-based assay for the kinetics of autophagic flux. Farkas T, Høyer-Hansen M, Jäättelä M. | Autophagy core |
| *CHKA* | ENSG00000110721 | CHK|CK|CKI|EK | 11 | 67820326 | 67888911 | Candidate kinases required for basal Autophagy | choline kinase alpha | Autophagy. 2009 Oct;5(7):1018–1025Identification of novel autophagy regulators by a luciferase-based assay for the kinetics of autophagic flux. Farkas T, Høyer-Hansen M, Jäättelä M. | Autophagy core |
| *CLK2* | ENSG00000176444|ENSG00000261893 | - | 1 | 155232659 | 155248282 | Candidate kinases required for basal Autophagy | CDC-like kinase 2 | Autophagy. 2009 Oct;5(7):1018–1025Identification of novel autophagy regulators by a luciferase-based assay for the kinetics of autophagic flux. Farkas T, Høyer-Hansen M, Jäättelä M. | Autophagy core |
| *BMX* | ENSG00000102010 | ETK|PSCTK2|PSCTK3 | X | 15482369 | 15574652 | Candidate kinases required for basal Autophagy | BMX non-receptor tyrosine kinase | Autophagy. 2009 Oct;5(7):1018–1025Identification of novel autophagy regulators by a luciferase-based assay for the kinetics of autophagic flux. Farkas T, Høyer-Hansen M, Jäättelä M. | Autophagy core |
| *ATF2* | ENSG00000115966 | CRE-BP1|CREB-2|CREB2|HB16|TREB7 | 2 | 175936978 | 176033110 | Positive regulator of Autophagy genes | activating transcription factor 2 | Stem Cells. 2015 Jun;33(6):1863-77. Icariin promotes angiogenic differentiation and prevents oxidative stress-induced autophagy in endothelial progenitor cells. Tang Y | Autophagy regulators |
| *ATF3* | ENSG00000162772 | - | 1 | 212738676 | 212794119 | Positive regulator of Autophagy genes | activating transcription factor 3 | Mol Pharmacol. 2014 May;85(5):682-91. Activating transcription factor 3 protects against pressure-overload heart failure via the autophagy molecule Beclin-1 pathway. Lin H | Autophagy regulators |
| *ATF4* | ENSG00000128272 | CREB-2|CREB2|TAXREB67|TXREB | 22 | 39915700 | 39918691 | Positive regulator of Autophagy genes | activating transcription factor 4 | The eIF2α/ATF4 pathway is essential for stress-induced autophagy gene expression; 2) J Bone Miner Res. 2017 Mar 17. Cartilage-specific Autophagy Deficiency Promotes ER Stress and Impairs Chondrogenesis in PERK-ATF4-CHOP Dependent Manner. Kang X | Autophagy regulators |
| *ATF6* | ENSG00000118217 | ACHM7|ATF6A | 1 | 161736084 | 161933860 | Positive regulator of Autophagy genes | activating transcription factor 6 | Cell Biol Toxicol. 2016 Apr;32(2):141-52. Quinocetone triggered ER stress-induced autophagy via ATF6/DAPK1-modulated mAtg9a trafficking. Zhou Y | Autophagy regulators |
| *CEBPB* | ENSG00000172216 | C/EBP-beta|IL6DBP|NF-IL6|TCF5 | 20 | 48807376 | 48809212 | Positive regulator of Autophagy genes | CCAAT/enhancer binding protein beta | Autophagy. 2016 Aug 2;12(8):1292-309. The circadian clock regulates autophagy directly through the nuclear hormone receptor Nr1d1/Rev-erbα and indirectly via Cebpb/(C/ebpβ) in zebrafish. Huang G | Autophagy regulators |
| *CREB1* | ENSG00000118260 | CREB|CREB-1 | 2 | 208394461 | 208468155 | Positive regulator of Autophagy genes | cAMP responsive element binding protein 1 | Autophagy. 2013 Dec;9(12):2069-86. MTOR inhibition attenuates DNA damage and apoptosis through autophagy-mediated suppression of CREB1. Wang Y | Autophagy regulators |
| *CRTC2* | ENSG00000160741 | TORC-2|TORC2 | 1 | 153920145 | 153931101 | Positive regulator of Autophagy genes | CREB regulated transcription coactivator 2 | Nature. 2014 Dec 4;516(7529):108-11. Transcriptional regulation of autophagy by an FXR-CREB axis. Seok S | Autophagy regulators |
| *DDIT3* | ENSG00000175197 | AltDDIT3|C/EBPzeta|CEBPZ|CHOP|CHOP-10|CHOP10|GADD153 | 12 | 57910371 | 57914300 | Positive regulator of Autophagy genes | DNA damage inducible transcript 3 | Nucleic Acids Res. 2013 Sep;41(16):7683-99. The eIF2α/ATF4 pathway is essential for stress-induced autophagy gene expression. B'chir W: 2)EMBO J. 2016 Mar 1;35(5):479-95. TFEB and TFE3 are novel components of the integrated stress response. Martina JA | Autophagy regulators |
| *E2F1* | ENSG00000101412 | E2F-1|RBAP1|RBBP3|RBP3 | 20 | 32263489 | 32274210 | Positive regulator of Autophagy genes | E2F transcription factor 1 | J Biol Chem. 2016 Jan 29;291(5):2043-54. The Retinoblastoma Tumor Suppressor Protein (pRb)/E2 Promoter Binding Factor 1 (E2F1) Pathway as a Novel Mediator of TGFβ-induced Autophagy. Korah J | Autophagy regulators |
| *EGR1* | ENSG00000120738 | AT225|G0S30|KROX-24|NGFI-A|TIS8|ZIF-268|ZNF225 | 5 | 137801179 | 137805004 | Positive regulator of Autophagy genes | early growth response 1 | Exp Cell Res. 2016 Jan 1;340(1):62-70. Egr-1 promotes hypoxia-induced autophagy to enhance chemo-resistance of hepatocellular carcinoma cells. Peng WX | Autophagy regulators |
| *EIF2A* | ENSG00000144895 | CDA02|EIF-2A|MST089|MSTP004|MSTP089 | 3 | 150264465 | 150302029 | Positive regulator of Autophagy genes | eukaryotic translation initiation factor 2A | Autophagy. 2016 May 3;12(5):770-83. Activation of the EIF2AK4-EIF2A/eIF2α-ATF4 pathway triggers autophagy response to Crohn disease-associated adherent-invasive Escherichia coli infection. Bretin A | Autophagy regulators |
| *EPAS1* | ENSG00000116016 | ECYT4|HIF2A|HLF|MOP2|PASD2|bHLHe73 | 2 | 46520806 | 46613836 | Positive regulator of Autophagy genes | endothelial PAS domain protein 1 | Autophagy. 2015;11(6):967-9. EPAS1/HIF-2α is a driver of mammalian pexophagy. Schönenberger MJ | Autophagy regulators |
| *ESR2* | ENSG00000140009 | ER-BETA|ESR-BETA|ESRB|ESTRB|Erb|NR3A2|ODG8 | 14 | 64550950 | 64804830 | Positive regulator of Autophagy genes | estrogen receptor 2 | Autophagy. 2016 Sep;12(9):1593-613. Inhibition of autophagosome-lysosome fusion by ginsenoside Ro via the ESR2-NCF1-ROS pathway sensitizes esophageal cancer cells to 5-fluorouracil-induced cell death via the CHEK1-mediated DNA damage checkpoint. Zheng K | Autophagy regulators |
| *FOS* | ENSG00000170345 | AP-1|C-FOS|p55 | 14 | 75745477 | 75748933 | Positive regulator of Autophagy genes | Fos proto-oncogene, AP-1 transcription factor subunit | Autophagy. 2015 Nov 2;11(11):2057-2073. A pivotal role of FOS-mediated BECN1/Beclin 1 upregulation in dopamine D2 and D3 receptor agonist-induced autophagy activation. Wang JD | Autophagy regulators |
| *FOXO1* | ENSG00000150907 | FKH1|FKHR|FOXO1A | 13 | 41129804 | 41240734 | Positive regulator of Autophagy genes | forkhead box O1 | Alcohol Clin Exp Res. 2017 Mar 16. FOXO1-AMPK-ULK1 Regulates Ethanol-induced Autophagy in Muscle by Enhanced ATG14 Association with the BECN1-PIK3C3 Complex. Hong-Brown LQ; 2) Cell Cycle. 2016 Nov;15(21):2856-2857. FoxO1-autophagy axis regulates lipid droplet growth via FSP27. Jash S; 3) Zhao Y, 2010 Cytosolic FoxO1 is essential for the induction ofautophagy and tumour suppressor activity. Nature Cell Biology 12665–675. | Autophagy regulators |
| *FOXO3* | ENSG00000118689 | AF6q21|FKHRL1|FKHRL1P2|FOXO2|FOXO3A | 6 | 108881038 | 109005977 | Positive regulator of Autophagy genes | forkhead box O3 | Autophagy. 2016 Oct 2;12(10):1804-1816. Activation of autophagy by FOXO3 regulates redox homeostasis during osteogenic differentiation. Gómez-Puerto MC | Autophagy regulators |
| *FOXO4* | ENSG00000184481 | AFX|AFX1|MLLT7 | X | 70316047 | 70323385 | Positive regulator of Autophagy genes | forkhead box O4 | 1) Nat Rev Urol. 2014 Aug;11(8):465-75. Molecular genetics and cellular features of TFE3 and TFEB fusion kidney cancers. Kauffman EC, Ricketts CJ, Rais-Bahrami S, Yang Y, Merino MJ, Bottaro DP, Srinivasan R, Linehan WM; 2)Nat Commun. 2015 Apr 10;6:6670. Regulation of autophagy and the ubiquitin-proteasome system by the FoxO transcriptional network during muscle atrophy. Milan G | Autophagy regulators |
| *FOXO6* | ENSG00000281518|ENSG00000204060 | 0 | 1 | 41827594 | 41849262 | Positive regulator of Autophagy genes | forkhead box O6 | J Endocrinol. 2017 May;233(2):R67-R79. FoxO integration of insulin signaling with glucose and lipid metabolism. Lee S | Autophagy regulators |
| *HEY2* | ENSG00000135547 | CHF1|GRIDLOCK|GRL|HERP1|HESR2|HRT2|bHLHb32 | 6 | 126068810 | 126082415 | Positive regulator of Autophagy genes | hes related family bHLH transcription factor with YRPW motif 2 | Curr Genomics. 2010 Jun;11(4):287-96. Transcription Factor CHF1/Hey2 Regulates Specific Pathways in Serum Stimulated Primary Cardiac Myocytes: Implications for Cardiac Hypertrophy. Yu M | Autophagy regulators |
| *HIF1A* | ENSG00000100644 | HIF-1-alpha|HIF-1A|HIF-1alpha|HIF1|HIF1-ALPHA|MOP1|PASD8|bHLHe78 | 14 | 62162231 | 62214976 | Positive regulator of Autophagy genes | hypoxia inducible factor 1 alpha subunit | Curr Cancer Drug Targets. 2017 Mar 15. HIF1A is overexpressed in medulloblastoma and its inhibition reduces proliferation and increases EPAS1 and ATG16L1 methylation. Cruzeiro GA | Autophagy regulators |
| *HSF1* | ENSG00000185122|ENSG00000284774 | HSTF1 | 8 | 145515280 | 145538385 | Positive regulator of Autophagy genes | heat shock transcription factor 1 | 1) Nat Rev Urol. 2014 Aug;11(8):465-75. Molecular genetics and cellular features of TFE3 and TFEB fusion kidney cancers. Kauffman EC, Ricketts CJ, Rais-Bahrami S, Yang Y, Merino MJ, Bottaro DP, Srinivasan R, Linehan WM; 2) Autophagy. 2017 Jan 2;13(1):133-148. HSF1 stress response pathway regulates autophagy receptor SQSTM1/p62-associated proteostasis. Watanabe Y | Autophagy regulators |
| *IRF1* | ENSG00000125347 | IRF-1|MAR | 5 | 131817301 | 131826490 | Positive regulator of Autophagy genes | interferon regulatory factor 1 | Mol Cell Oncol. 2015 Apr 14;3(1):e1023928. Linking autophagy with inflammation through IRF1 signaling in ER+ breast cancer. Cook KL | Autophagy regulators |
| *LMX1A* | ENSG00000162761 | DFNA7|LMX1|LMX1.1 | 1 | 165171104 | 165325952 | Positive regulator of Autophagy genes | LIM homeobox transcription factor 1 alpha | Nat Neurosci. 2015 Jun;18(6):826-35Dopaminergic control of autophagic-lysosomal function implicates Lmx1b in Parkinson's disease. Laguna A | Autophagy regulators |
| *LMX1B* | ENSG00000136944 | LMX1.2|NPS1 | 9 | 129376722 | 129463311 | Positive regulator of Autophagy genes | LIM homeobox transcription factor 1 beta | Nat Neurosci. 2015 Jun;18(6):826-35Dopaminergic control of autophagic-lysosomal function implicates Lmx1b in Parkinson's disease. Laguna A | Autophagy regulators |
| *MAPK1* | ENSG00000100030 | ERK|ERK-2|ERK2|ERT1|MAPK2|P42MAPK|PRKM1|PRKM2|p38|p40|p41|p41mapk|p42-MAPK | 22 | 22108789 | 22221970 | Positive regulator of Autophagy genes | mitogen-activated protein kinase 1 | Nature. 2014 Dec 4;516(7529):108-11Transcriptional regulation of autophagy by an FXR-CREB axis. Seok S; 2) Autophagy. 2015;11(2):332-43. Mitophagy is primarily due to alternative autophagy and requires the MAPK1 and MAPK14 signaling pathways. Hirota Y | Autophagy regulators |
| *MAPK3* | ENSG00000102882 | ERK-1|ERK1|ERT2|HS44KDAP|HUMKER1A|P44ERK1|P44MAPK|PRKM3|p44-ERK1|p44-MAPK | 16 | 30125426 | 30134827 | Positive regulator of Autophagy genes | mitogen-activated protein kinase 3 | Autophagy. 2016;12(3):592-3. MAPK1/3 regulate hepatic lipid metabolism via ATG7-dependent autophagy. Xiao Y | Autophagy regulators |
| *MEF2A* | ENSG00000068305 | ADCAD1|RSRFC4|RSRFC9|mef2 | 15 | 100017370 | 100256671 | Positive regulator of Autophagy genes | myocyte enhancer factor 2A | Autophagy. 2014 Jun;10(6):1015-35. Disruption of chaperone-mediated autophagy-dependent degradation of MEF2A by oxidative stress-induced lysosome destabilization. Zhang L | Autophagy regulators |
| *MITF* | ENSG00000187098 | CMM8|COMMAD|MI|WS2|WS2A|bHLHe32 | 3 | 69788586 | 70017488 | Positive regulator of Autophagy genes | melanogenesis associated transcription factor | PLoS One. 2017 Mar 16;12(3):e0173771. Lysosomal adaptation: How cells respond to lysosomotropic compounds. Lu S | Autophagy regulators |
| *MYC* | ENSG00000136997 | MRTL|MYCC|bHLHe39|c-Myc | 8 | 128747680 | 128753674 | Positive regulator of Autophagy genes | MYC proto-oncogene, bHLH transcription factor | Autophagy. 2017 Mar 4;13(3):554-566. MIR7-3HG, a MYC-dependent modulator of cell proliferation, inhibits autophagy by a regulatory loop involving AMBRA1. Capizzi M | Autophagy regulators |
| *NACC1* | ENSG00000160877 | BEND8|BTBD14B|BTBD30|NAC-1|NAC1|NECFM | 19 | 13228917 | 13251955 | Positive regulator of Autophagy genes | nucleus accumbens associated 1 | Oncogene. 2012 Feb 23;31(8):1055-64. NAC1 modulates sensitivity of ovarian cancer cells to cisplatin by altering the HMGB1-mediated autophagic response. Zhang Y | Autophagy regulators |
| *NFE2L1* | ENSG00000082641 | LCR-F1|NRF1|TCF11 | 17 | 46125691 | 46138849 | Positive regulator of Autophagy genes | nuclear factor, erythroid 2 like 1 | Cell Cycle. 2015;14(13):2011-7. mTORC1 signaling activates NRF1 to increase cellular proteasome levels. Zhang Y | Autophagy regulators |
| *NFE2L2* | ENSG00000116044 | HEBP1|IMDDHH|NRF2|Nrf-2 | 2 | 178092323 | 178257425 | Positive regulator of Autophagy genes | nuclear factor, erythroid 2 like 2 | J Biol Chem. 2017 Mar 22. pii: jbc.M116.773986. Nuclear factor erythroid 2-related factor 2 enhances carcinogenesis by suppressing apoptosis and promoting autophagy in nickel-transformed cells. Son YO | Autophagy regulators |
| *NFKB1* | ENSG00000109320 | CVID12|EBP-1|KBF1|NF-kB|NF-kB1|NF-kappa-B1|NF-kappaB|NF-kappabeta|NFKB-p105|NFKB-p50|NFkappaB | 4 | 103422486 | 103538459 | Positive regulator of Autophagy genes | nuclear factor kappa B subunit 1 | Autophagy. 2017 Jan 2;13(1):149-168. GFRA1 promotes cisplatin-induced chemoresistance in osteosarcoma by inducing autophagy. Kim M | Autophagy regulators |
| *NRF1* | ENSG00000106459 | ALPHA-PAL | 7 | 129251555 | 129396922 | Positive regulator of Autophagy genes | nuclear respiratory factor 1 | Int J Mol Sci. 2016 Dec 13;17(12). Estrogenic Endocrine Disrupting Chemicals Influencing NRF1 Regulated Gene Networks in the Development of Complex Human Brain Diseases. Preciados M | Autophagy regulators |
| *PPARA* | ENSG00000186951 | NR1C1|PPAR|PPARalpha|hPPAR | 22 | 46546424 | 46639653 | Positive regulator of Autophagy genes | peroxisome proliferator activated receptor alpha | Nature. 2014 Dec 4;516(7529):112-5. Nutrient-sensing nuclear receptors coordinate autophagy. Lee JM | Autophagy regulators |
| *PPARG* | ENSG00000132170 | CIMT1|GLM1|NR1C3|PPARG1|PPARG2|PPARG5|PPARgamma | 3 | 12328867 | 12475855 | Positive regulator of Autophagy genes | peroxisome proliferator activated receptor gamma | Endocr Relat Cancer. 2016 Oct;23(10):839-56. Social isolation induces autophagy in the mouse mammary gland: link to increased mammary cancer risk. Sumis A | Autophagy regulators |
| *PPARGC1A* | ENSG00000109819 | LEM6|PGC-1(alpha)|PGC-1alpha|PGC-1v|PGC1|PGC1A|PPARGC1 | 4 | 23756664 | 23905712 | Positive regulator of Autophagy genes | PPARG coactivator 1 alpha | Physiol Rep. 2016 Feb;4(3). pii: e12698. PGC-1α promotes exercise-induced autophagy in mouse skeletal muscle. Halling JF | Autophagy regulators |
| *PPARGC1B* | ENSG00000155846 | ERRL1|PERC|PGC-1(beta)|PGC1B | 5 | 149109861 | 149234585 | Positive regulator of Autophagy genes | PPARG coactivator 1 beta | Oncol Rep. 2013 Oct;30(4):1631-8. Apoptosis induced by PGC-1β in breast cancer cells is mediated by the mTOR pathway. Wang L | Autophagy regulators |
| *RARA* | ENSG00000131759 | NR1B1|RAR | 17 | 38465444 | 38513094 | Positive regulator of Autophagy genes | retinoic acid receptor alpha | Autophagy. 2015;11(3):460-71. Retinoic acid-induced IgG production in TLR-activated human primary B cells involves ULK1-mediated autophagy. Eriksen AB | Autophagy regulators |
| *RELA* | ENSG00000173039 | CMCU|NFKB3|p65 | 11 | 65421067 | 65430565 | Positive regulator of Autophagy genes | RELA proto-oncogene, NF-kB subunit | J Cell Mol Med. 2017 Jun 23. Catalase ameliorates diabetes-induced cardiac injury through reduced p65/RelA- mediated transcription of BECN1. Wang X | Autophagy regulators |
| *RELB* | ENSG00000104856 | I-REL|IMD53|IREL|REL-B | 19 | 45504688 | 45541452 | Positive regulator of Autophagy genes | RELB proto-oncogene, NF-kB subunit | Cell Death Dis. 2015 Oct 22;6:e1942. Autophagy-induced RelB/p52 activation mediates tumour-associated macrophage repolarisation and suppression of hepatocellular carcinoma by natural compound baicalin. Tan HY | Autophagy regulators |
| *RFX1* | ENSG00000132005|ENSG00000288283 | EFC|RFX | 19 | 14072350 | 14117851 | Positive regulator of Autophagy genes | regulatory factor X1 | Oncotarget. 2014 Jul 15;5(13):4909-19. RFX1-dependent activation of SHP-1 induces autophagy by a novel obatoclax derivative in hepatocellular carcinoma cells. Su JC | Autophagy regulators |
| *SIRT1* | ENSG00000096717 | SIR2|SIR2L1|SIR2alpha | 10 | 69644427 | 69678147 | Positive regulator of Autophagy genes | sirtuin 1 | Int J Mol Med. 2019 May;43(5):2033-2043. Sirt1 promotes autophagy and inhibits apoptosis to protect cardiomyocytes from hypoxic stress. Luo G | Autophagy regulators |
| *SIRT2* | ENSG00000068903|ENSG00000283100 | SIR2|SIR2L|SIR2L2 | 19 | 39369197 | 39390502 | Positive regulator of Autophagy genes | sirtuin 2 | 1)Biochem Biophys Res Commun. 2017 Jul 8;488(4):603-608. Sirtuin inhibition leads to autophagy and apoptosis in porcine preimplantation blastocysts. Kim MG; 2) Mol Neurobiol. 2017 Aug;54(6):4021-4040. Mitochondrial Metabolism Power SIRT2-Dependent Deficient Traffic Causing Alzheimer's-Disease Related Pathology. Silva DF | Autophagy regulators |
| *SIRT3* | ENSG00000142082 | SIR2L3 | 11 | 215458 | 236931 | Positive regulator of Autophagy genes | sirtuin 3 | Biochim Biophys Acta. 2017 Aug;1863(8):1973-1983. Sirt3 deficiency exacerbates diabetic cardiac dysfunction: Role of Foxo3A-Parkin-mediated mitophagy. Yu W | Autophagy regulators |
| *SIRT4* | ENSG00000089163 | SIR2L4 | 12 | 120740119 | 120751052 | Positive regulator of Autophagy genes | sirtuin 4 | Autophagy. 2010 Oct;6(7):986-7. Traf6 and A20 differentially regulate TLR4-induced autophagy by affecting the ubiquitination of Beclin 1. Shi CS; 2) J Biomol Struct Dyn. 2016 Nov 18:1-14. Molecular modeling, dynamics studies and density functional theory approaches to identify potential inhibitors of SIRT4 protein from Homo sapiens : a novel target for the treatment of type 2 diabetes. Choubey SK | Autophagy regulators |
| *SIRT5* | ENSG00000124523 | SIR2L5 | 6 | 13574816 | 13614790 | Positive regulator of Autophagy genes | sirtuin 5 | Autophagy. 2015;11(2):253-70. SIRT5 regulation of ammonia-induced autophagy and mitophagy. Polletta L | Autophagy regulators |
| *SIRT6* | ENSG00000077463 | SIR2L6 | 19 | 4174106 | 4182601 | Positive regulator of Autophagy genes | sirtuin 6 | FEBS J. 2017 May;284(9):1324-1337. SIRT6 reduces macrophage foam cell formation by inducing autophagy and cholesterol efflux under ox-LDL condition. He J | Autophagy regulators |
| *SIRT7* | ENSG00000187531 | SIR2L7 | 17 | 79869815 | 79879199 | Positive regulator of Autophagy genes | sirtuin 7 | Circulation. 2015 Sep 22;132(12):1081-93. Sirt7 Contributes to Myocardial Tissue Repair by Maintaining Transforming Growth Factor-β Signaling Pathway. Araki S | Autophagy regulators |
| *SOX2* | ENSG00000181449 | ANOP3|MCOPS3 | 3 | 181429714 | 181432221 | Positive regulator of Autophagy genes | SRY-box 2 | Autophagy. 2017 Jul 19:0. BNIP3L-dependent Mitophagy Accounts for Mitochondrial Clearance during Three Factors Induced Somatic Cell Reprogramming. Xiang G | Autophagy regulators |
| *SP1* | ENSG00000185591 | - | 12 | 53773960 | 53810230 | Positive regulator of Autophagy genes | Sp1 transcription factor | Biochim Biophys Acta. 2017 Jul 13. pii: S0304-4165(17)30219-2. Pneumolysin-induced autophagy contributes to inhibition of osteoblast differentiation through downregulation of Sp1 in human osteosarcoma cells. Kim J; 2)Oncotarget. 2015 Jul 30;6(21):18469-83. PP2A inhibitors arrest G2/M transition through JNK/Sp1- dependent down-regulation of CDK1 and autophagy-dependent up-regulation of p21. Gong FR | Autophagy regulators |
| *STAT3* | ENSG00000168610 | ADMIO|ADMIO1|APRF|HIES | 17 | 40465342 | 40540586 | Positive regulator of Autophagy genes | signal transducer and activator of transcription 3 | PLoS One. 2017 Jul 7;12(7):e0179835. STAT3 balances myocyte hypertrophy vis-à-vis autophagy in response to Angiotensin II by modulating the AMPKα/mTOR axis. Chen L | Autophagy regulators |
| *TFE3* | ENSG00000068323 | RCCP2|RCCX1|TFEA|bHLHe33 | X | 48886242 | 48901012 | Positive regulator of Autophagy genes | transcription factor binding to IGHM enhancer 3 [Homo sapiens | PLoS One. 2017 Mar 16;12(3):e0173771. Lysosomal adaptation: How cells respond to lysosomotropic compounds. Lu S; Methods Enzymol. 2017;588:61-78. Methods to Monitor and Manipulate TFEB Activity During Autophagy. Medina DL | Autophagy regulators |
| *TFEB* | ENSG00000112561 | ALPHATFEB|BHLHE35|TCFEB | 6 | 41651716 | 41703997 | Positive regulator of Autophagy genes | transcription factor EB | PLoS One. 2017 Mar 16;12(3):e0173771. Lysosomal adaptation: How cells respond to lysosomotropic compounds. Lu S; Methods Enzymol. 2017;588:61-78. Methods to Monitor and Manipulate TFEB Activity During Autophagy. Medina DL | Autophagy regulators |
| *TFEC* | ENSG00000105967 | TCFEC|TFE-C|TFEC-L|TFECL|bHLHe34|hTFEC-L | 7 | 115575202 | 115799950 | Positive regulator of Autophagy genes | transcription factor EC | Pharmacol Res. 2015 Sep;99:36-43. The MITF family of transcription factors: Role in endolysosomal biogenesis, Wnt signaling, and oncogenesis. Ploper D | Autophagy regulators |
| *TNFAIP3* | ENSG00000118503 | A20|AISBL|OTUD7C|TNFA1P2 | 6 | 138188351 | 138204449 | Positive regulator of Autophagy genes | TNF alpha induced protein 3 | Autophagy. 2015;11(7):1052-62. TNFAIP3 promotes survival of CD4 T cells by restricting MTOR and promoting autophagy. Matsuzawa Y | Autophagy regulators |
| *TP53* | ENSG00000141510 | BCC7|BMFS5|LFS1|P53|TRP53 | 17 | 7565097 | 7590856 | Positive regulator of Autophagy genes | tumor protein p53 | J Cell Biol. 2013 Apr 29;201(3):427-37. Deacetylation of p53 induces autophagy by suppressing Bmf expression. Contreras AU | Autophagy regulators |
| *XBP1* | ENSG00000100219 | TREB-5|TREB5|XBP-1|XBP2 | 22 | 29190543 | 29196585 | Positive regulator of Autophagy genes | X-box binding protein 1 | Sci Rep. 2017 Jun 30;7(1):4442. XBP1-FoxO1 interaction regulates ER stress-induced autophagy in auditory cells. Kishino A | Autophagy regulators |
| *YY1* | ENSG00000100811 | DELTA|GADEVS|INO80S|NF-E1|UCRBP|YIN-YANG-1 | 14 | 100704635 | 100749129 | Positive regulator of Autophagy genes | YY1 transcription factor | Autophagy. 2014 Aug;10(8):1442-53. YY1-MIR372-SQSTM1 regulatory axis in autophagy. Feng L | Autophagy regulators |
| *ZEB1* | ENSG00000148516 | AREB6|BZP|DELTAEF1|FECD6|NIL2A|PPCD3|TCF8|ZFHEP|ZFHX1A | 10 | 31607424 | 31818742 | Positive regulator of Autophagy genes | zinc finger E-box binding homeobox 1 | Am J Transl Res. 2017 Mar 15;9(3):1357-1368. Downregulation of miR-429 contributes to the development of drug resistance in epithelial ovarian cancer by targeting ZEB1. Zou J | Autophagy regulators |
| *ATF5* | ENSG00000169136 | ATFX|HMFN0395 | 19 | 50431959 | 50437192 | Negative regulator of Autophagy genes | activating transcription factor 5 | Blood. 2011 Sep 8;118(10):2840-8. BCR-ABL suppresses autophagy through ATF5-mediated regulation of mTOR transcription. Sheng Z | Autophagy regulators |
| *BDNF* | ENSG00000176697 | ANON2|BULN2 | 11 | 27676440 | 27743605 | Negative regulator of Autophagy genes | brain derived neurotrophic factor | Int J Mol Sci. 2017 Mar 3;18(3). pii: E545. More Insight into BDNF against Neurodegeneration: Anti-Apoptosis, Anti-Oxidation, and Suppression of Autophagy. Chen SD | Autophagy regulators |
| *JUN* | ENSG00000177606 | AP-1|AP1|c-Jun|cJUN|p39 | 1 | 59246465 | 59249785 | Negative regulator of Autophagy genes | Jun proto-oncogene, AP-1 transcription factor subunit | Cell Res. 2017 Feb;27(2):184-201. Regulation of mATG9 trafficking by Src- and ULK1-mediated phosphorylation in basal and starvation-induced autophagy. Zhou C; 2)Autophagy. 2010 May;6(4):566-7Jun proteins inhibit autophagy and induce cell death. Yogev O | Autophagy regulators |
| *JUNB* | ENSG00000171223 | AP-1 | 19 | 12902310 | 12904124 | Negative regulator of Autophagy genes | JunB proto-oncogene, AP-1 transcription factor subunit | Autophagy. 2010 May;6(4):566-7. Jun proteins inhibit autophagy and induce cell death. Yogev O | Autophagy regulators |
| *KEAP1* | ENSG00000079999 | INrf2|KLHL19 | 19 | 10596796 | 10614417 | Negative regulator of Autophagy genes | kelch like ECH associated protein 1 | Acta Neuropathol Commun. 2016 Oct 31;4(1):115. Activation of the Keap1/Nrf2 stress response pathway in autophagic vacuolar myopathies. Duleh S | Autophagy regulators |
| *NR1H4* | ENSG00000012504 | BAR|FXR|HRR-1|HRR1|PFIC5|RIP14 | 12 | 100867486 | 100958191 | Negative regulator of Autophagy genes | nuclear receptor subfamily 1 group H member 4 | Nature. 2014 Dec 4;516(7529):112-5. Nutrient-sensing nuclear receptors coordinate autophagy. Lee JM | Autophagy regulators |
| *STAT1* | ENSG00000115415 | CANDF7|IMD31A|IMD31B|IMD31C|ISGF-3|STAT91 | 2 | 191829084 | 191885686 | Negative regulator of Autophagy genes | signal transducer and activator of transcription 1 | J Biol Chem. 2017 Feb 3;292(5):1899-1909. Regulation of ULK1 Expression and Autophagy by STAT1. Goldberg AA | Autophagy regulators |
| *ZKSCAN3* | ENSG00000189298 | ZF47|ZFP306|ZNF306|ZNF309|ZSCAN13|ZSCAN35|Zfp47|dJ874C20.1|dJ874C20.1.|zfp-47 | 6 | 28317691 | 28336947 | Negative regulator of Autophagy genes | zinc finger with KRAB and SCAN domains 3 | Mol Cell. 2013 Apr 11;50(1):16-28. ZKSCAN3 is a master transcriptional repressor of autophagy. Chauhan | Autophagy regulators |
| *BNIP3* | ENSG00000176171 | NIP3 | 10 | 133781578 | 133795435 | Mitophagy core | BCL2/adenovirus E1B 19kDa interacting protein 3 | J Biol Chem. 2012 Jun 1;287(23):19094-104. Microtubule-associated protein 1 light chain 3 (LC3) interacts with Bnip3 protein to selectively remove endoplasmic reticulum and mitochondria via autophagy. Hanna RA, Quinsay MN, Orogo AM, Giang K, Rikka S, Gustafsson ÅB. | Mitophagy |
| *MAPK1* | ENSG00000100030 | ERK|ERK-2|ERK2|ERT1|MAPK2|P42MAPK|PRKM1|PRKM2|p38|p40|p41|p41mapk|p42-MAPK | 22 | 22108789 | 22221970 | Mitophagy core | mitogen-activated protein kinase 1 | Autophagy. 2015;11(2):332-43. Mitophagy is primarily due to alternative autophagy and requires the MAPK1 and MAPK14 signaling pathways. Hirota Y, Yamashita S, Kurihara Y, Jin X, Aihara M, Saigusa T, Kang D, Kanki T. | Mitophagy |
| *MAPK14* | ENSG00000112062 | CSBP|CSBP1|CSBP2|CSPB1|EXIP|Mxi2|PRKM14|PRKM15|RK|SAPK2A|p38|p38ALPHA | 6 | 35995488 | 36079013 | Mitophagy core | mitogen-activated protein kinase 14 | Autophagy. 2015;11(2):332-43. Mitophagy is primarily due to alternative autophagy and requires the MAPK1 and MAPK14 signaling pathways. Hirota Y, Yamashita S, Kurihara Y, Jin X, Aihara M, Saigusa T, Kang D, Kanki T. | Mitophagy |
| *SMURF1* | ENSG00000198742|ENSG00000284126 | - | 7 | 98625061 | 98741723 | Mitophagy core | SMAD specific E3 ubiquitin protein ligase 1 | Nature. 2011 Dec 1;480(7375):113-7. Image-based genome-wide siRNA screen identifies selective autophagy factors. Orvedahl A, Sumpter R Jr, Xiao G, Ng A, Zou Z, Tang Y, Narimatsu M, Gilpin C, Sun Q, Roth M, Forst CV, Wrana JL, Zhang YE, Luby-Phelps K, Xavier RJ, Xie Y, Levine B. | Mitophagy |
| *ATP13A2* | ENSG00000159363 | CLN12|HSA9947|KRPPD|PARK9|SPG78 | 1 | 17312453 | 17338423 | Mitophagy core | ATPase type 13A2 | Autophagy. 2013 Nov 1;9(11):1828-36. Proteolytic processing of Atg32 by the mitochondrial i-AAA protease Yme1 regulates mitophagy. Wang K, Jin M, Liu X, Klionsky DJ. | Mitophagy |
| *SPATA18* | ENSG00000163071 | Mieap|SPETEX1 | 4 | 52917497 | 52963458 | Mitophagy core | spermatogenesis associated 18 | PLoS One. 2011 Jan 17;6(1):e16054. Possible existence of lysosome-like organella within mitochondria and its role in mitochondrial quality control. Miyamoto Y, Kitamura N, Nakamura Y, Futamura M, Miyamoto T, Yoshida M, Ono M, Ichinose S, Arakawa H. | Mitophagy |
| *TBK1* | ENSG00000183735 | FTDALS4|IIAE8|NAK|T2K | 12 | 64845660 | 64895888 | Mitophagy core | TANK-binding kinase 1 | Proc Natl Acad Sci U S A. 2016 Apr 12;113(15):4039-44. Phosphorylation of OPTN by TBK1 enhances its binding to Ub chains and promotes selective autophagy of damaged mitochondria. Richter B, Sliter DA, Herhaus L, Stolz A, Wang C, Beli P, Zaffagnini G, Wild P, Martens S, Wagner SA, Youle RJ, Dikic I. | Mitophagy |
| *CERS1* | ENSG00000223802 | EPM8|GDF-1|GDF1|LAG1|LASS1|UOG1 | 19 | 18979361 | 19007536 | Mitophagy core | ceramide synthase 1 | Nat Chem Biol. 2012 Oct;8(10):831-8. Ceramide targets autophagosomes to mitochondria and induces lethal mitophagy. Sentelle RD, Senkal CE, Jiang W, Ponnusamy S, Gencer S, Selvam SP, Ramshesh VK, Peterson YK, Lemasters JJ, Szulc ZM, Bielawski J, Ogretmen B. | Mitophagy |
| *VCP* | ENSG00000165280 | CDC48|TERA|p97 | 9 | 35056061 | 35073246 | Mitophagy core | valosin containing protein | J Cell Biol. 2010 Dec 27;191(7):1367-80. Proteasome and p97 mediate mitophagy and degradation of mitofusins induced by Parkin. Tanaka A, Cleland MM, Xu S, Narendra DP, Suen DF, Karbowski M, Youle RJ. | Mitophagy |
| *HDAC6* | ENSG00000094631 | CPBHM|HD6|JM21|PPP1R90 | X | 48659784 | 48683392 | Mitophagy core | histone deacetylase 6 | J Cell Biol. 2010 May 17;189(4):671-9. Disease-causing mutations in parkin impair mitochondrial ubiquitination, aggregation, and HDAC6-dependent mitophagy. Lee JY, Nagano Y, Taylor JP, Lim KL, Yao TP. | Mitophagy |
| *USP8* | ENSG00000138592 | HumORF8|PITA4|SPG59|UBPY | 15 | 50716577 | 50793280 | Mitophagy core | ubiquitin specific peptidase 8 | Autophagy. 2015 Nov 3;11(2):428-9. USP8 and PARK2/parkin-mediated mitophagy. Durcan TM, Fon EA. | Mitophagy |
| *PINK1* | ENSG00000158828 | BRPK|PARK6 | 1 | 20959948 | 20978004 | Mitophagy core | PTEN induced putative kinase 1 | Nature. 2015 Aug 20;524(7565):309-314. The ubiquitin kinase PINK1 recruits autophagy receptors to induce mitophagy. Lazarou M, Sliter DA, Kane LA, Sarraf SA, Wang C, Burman JL, Sideris DP, Fogel AI, Youle RJ. | Mitophagy |
| *MUL1* | ENSG00000090432 | C1orf166|GIDE|MAPL|MULAN|RNF218 | 1 | 20825943 | 20834654 | Mitophagy core | mitochondrial E3 ubiquitin protein ligase 1 | Autophagy. 2015;11(8):1216-29. Mitochondrial outer-membrane E3 ligase MUL1 ubiquitinates ULK1 and regulates selenite-induced mitophagy. Li J, Qi W, Chen G, Feng D, Liu J, Ma B, Zhou C, Mu C, Zhang W, Chen Q, Zhu Y. | Mitophagy |
| *FBXO7* | ENSG00000100225 | FBX|FBX07|FBX7|PARK15|PKPS | 22 | 32870663 | 32894818 | Mitophagy core | F-box protein 7 | Nat Neurosci. 2013 Sep;16(9):1257-65. The Parkinson's disease-linked proteins Fbxo7 and Parkin interact to mediate mitophagy. Burchell VS, Nelson DE, Sanchez-Martinez A, Delgado-Camprubi M, Ivatt RM, Pogson JH, Randle SJ, Wray S, Lewis PA, Houlden H, Abramov AY, Hardy J, Wood NW, Whitworth AJ, Laman H, Plun-Favreau H. | Mitophagy |
| *TBC1D17* | ENSG00000104946 | - | 19 | 50380682 | 50392005 | Mitophagy core | TBC1 domain family, member 17 | Elife. 2014 Feb 25;3:e01612. Mitochondrial Rab GAPs govern autophagosome biogenesis during mitophagy. Yamano K, Fogel AI, Wang C, van der Bliek AM, Youle RJ. | Mitophagy |
| *TBC1D15* | ENSG00000121749 | RAB7-GAP | 12 | 72233487 | 72320629 | Mitophagy core | TBC1 domain family, member 15 | Elife. 2014 Feb 25;3:e01612. Mitochondrial Rab GAPs govern autophagosome biogenesis during mitophagy. Yamano K, Fogel AI, Wang C, van der Bliek AM, Youle RJ. | Mitophagy |
| *RHEB* | ENSG00000106615 | RHEB2 | 7 | 151163098 | 151217206 | Mitophagy core | Ras homolog enriched in brain | Cell Metab. 2013 May 7;17(5):719-30. Rheb regulates mitophagy induced by mitochondrial energetic status. Melser S, Chatelain EH, Lavie J, Mahfouf W, Jose C, Obre E, Goorden S, Priault M, Elgersma Y, Rezvani HR, Rossignol R, Bénard G. | Mitophagy |
| *RNF185* | ENSG00000138942 | - | 22 | 31556168 | 31603005 | Mitophagy core | ring finger protein 185 | PLoS One. 2011;6(9):e24367. RNF185, a novel mitochondrial ubiquitin E3 ligase, regulates autophagy through interaction with BNIP1. Tang F, Wang B, Li N, Wu Y, Jia J, Suo T, Chen Q, Liu YJ, Tang J. | Mitophagy |
| *PARK7* | ENSG00000116288 | DJ-1|DJ1|GATD2|HEL-S-67p | 1 | 8014351 | 8045565 | Mitophagy core | parkinson protein 7 | Trends Biochem Sci. 2015 Apr;40(4):200–210. Mitochondrial dysfunction and mitophagy in Parkinson's: from familial to sporadic disease. Ryan BJ, Hoek S, Fon EA, Wade-Martins R. | Mitophagy |
| *PARK2* | ENSG00000185345 | PARKIN|PRKN | 6 | 161768452 | 163148803 | Mitophagy core | parkinson protein 2, E3 ubiquitin protein ligase (parkin) | J Cell Biol. 2008 Dec 1;183(5):795-803. Parkin is recruited selectively to impaired mitochondria and promotes their autophagy. Narendra D, Tanaka A, Suen DF, Youle RJ. | Mitophagy |
| *TGM2* | ENSG00000198959 | TG(C)|TGC | 20 | 36756863 | 36794980 | Mitophagy core | transglutaminase 2 | Cell Death Differ. 2015 Mar;22(3):408-18. Transglutaminase 2 ablation leads to mitophagy impairment associated with a metabolic shift towards aerobic glycolysis. Rossin F, D'Eletto M, Falasca L, Sepe S, Cocco S, Fimia GM, Campanella M, Mastroberardino PG, Farrace MG, Piacentini M. | Mitophagy |
| *BCL2L13* | ENSG00000099968 | BCL-RAMBO|Bcl2-L-13|MIL1 | 22 | 18111621 | 18213388 | Mitophagy core | BCL2-like 13 (apoptosis facilitator) | Autophagy. 2015;11(10):1932-3. BCL2L13 is a mammalian homolog of the yeast mitophagy receptor Atg32. Otsu K, Murakawa T, Yamaguchi O. | Mitophagy |
| *HSPA1L* | ENSG00000204390|ENSG00000206383|ENSG00000226704|ENSG00000234258|ENSG00000236251 | HSP70-1L|HSP70-HOM|HSP70T|hum70t | 6 | 31777396 | 31783437 | Mitophagy core | Heat Shock Protein Family A (Hsp70) Member 1 Like | Redox Biol. 2015;4:6–13. Mitochondrial dynamics and mitochondrial quality control. Ni HM, Williams JA, Ding WX. | Mitophagy |
| *BAG4* | ENSG00000156735 | BAG-4|SODD | 8 | 38034051 | 38070819 | Mitophagy core | Bcl2-associated athanogene 4 (BAG4, | Redox Biol. 2015;4:6–13. Mitochondrial dynamics and mitochondrial quality control. Ni HM, Williams JA, Ding WX. | Mitophagy |
| *TOMM7* | ENSG00000196683 | TOM7 | 7 | 22852251 | 22862470 | Mitophagy core | Translocase Of Outer Mitochondrial Membrane 7 | Redox Biol. 2015;4:6–13. Mitochondrial dynamics and mitochondrial quality control. Ni HM, Williams JA, Ding WX. | Mitophagy |
| *AMBRA1* | ENSG00000110497 | DCAF3|WDR94 | 11 | 46417964 | 46615675 | Mitophagy core | autophagy/beclin-1 regulator 1 | J Neurosci. 2011 Jul 13;31(28):10249-61. Parkin interacts with Ambra1 to induce mitophagy. Van Humbeeck C1, Cornelissen T, Hofkens H, Mandemakers W, Gevaert K, De Strooper B, Vandenberghe W. | Mitophagy |
| *BECN1* | ENSG00000126581 | ATG6|VPS30|beclin1 | 17 | 40962152 | 40985367 | Mitophagy core | beclin 1, autophagy related | Hum Mol Genet. 2011 Jun 1;20(11):2091–2102. Parkin mediates beclin-dependent autophagic clearance of defective mitochondria and ubiquitinated Abeta in AD models. Khandelwal PJ, Herman AM, Hoe HS, Rebeck GW, Moussa CE. | Mitophagy |
| *UBE2D2* | ENSG00000131508 | E2(17)KB2|PUBC1|UBC4|UBC4/5|UBCH4|UBCH5B | 5 | 138906016 | 139008018 | Mitophagy core | ubiquitin-conjugating enzyme E2D 2 | J Cell Sci. 2014 Aug 1;127(Pt 15):3280-93. The ubiquitin-conjugating enzymes UBE2N, UBE2L3 and UBE2D2/3 are essential for Parkin-dependent mitophagy. Geisler S, Vollmer S, Golombek S, Kahle PJ. | Mitophagy |
| *UBE2L3* | ENSG00000185651 | E2-F1|L-UBC|UBCH7|UbcM4 | 22 | 21903736 | 21978323 | Mitophagy core | ubiquitin-conjugating enzyme E2L 3 | J Cell Sci. 2014 Aug 1;127(Pt 15):3280-93. The ubiquitin-conjugating enzymes UBE2N, UBE2L3 and UBE2D2/3 are essential for Parkin-dependent mitophagy. Geisler S, Vollmer S, Golombek S, Kahle PJ. | Mitophagy |
| *UBE2D3* | ENSG00000109332 | E2(17)KB3|UBC4/5|UBCH5C | 4 | 103715540 | 103790053 | Mitophagy core | ubiquitin-conjugating enzyme E2D 3 | J Cell Sci. 2014 Aug 1;127(Pt 15):3280-93. The ubiquitin-conjugating enzymes UBE2N, UBE2L3 and UBE2D2/3 are essential for Parkin-dependent mitophagy. Geisler S, Vollmer S, Golombek S, Kahle PJ. | Mitophagy |
| *UBE2N* | ENSG00000177889 | HEL-S-71|UBC13|UBCHBEN|UBC13|UbcH-ben|UbcH13 | 12 | 93799449 | 93836038 | Mitophagy core | ubiquitin-conjugating enzyme E2N | J Cell Sci. 2014 Aug 1;127(Pt 15):3280-93. The ubiquitin-conjugating enzymes UBE2N, UBE2L3 and UBE2D2/3 are essential for Parkin-dependent mitophagy. Geisler S, Vollmer S, Golombek S, Kahle PJ. | Mitophagy |
| *RAB5A* | ENSG00000144566 | RAB5 | 3 | 19988571 | 20026667 | Mitophagy core | RAB5A, member RAS oncogene family | Nat Commun. 2017 Jan 30;8:14050. A Rab5 endosomal pathway mediates Parkin-dependent mitochondrial clearance. Hammerling BC, Najor RH, Cortez MQ, Shires SE, Leon LJ, Gonzalez ER, Boassa D, Phan S, Thor A, Jimenez RE, Li H, Kitsis RN, Dorn GW II, Sadoshima J, Ellisman MH, Gustafsson ÅB. | Mitophagy |
| *RAB5B* | ENSG00000111540 | - | 12 | 56367697 | 56388490 | Mitophagy core | RAB5B, member RAS oncogene family | Nat Commun. 2017 Jan 30;8:14050. A Rab5 endosomal pathway mediates Parkin-dependent mitochondrial clearance. Hammerling BC, Najor RH, Cortez MQ, Shires SE, Leon LJ, Gonzalez ER, Boassa D, Phan S, Thor A, Jimenez RE, Li H, Kitsis RN, Dorn GW II, Sadoshima J, Ellisman MH, Gustafsson ÅB. | Mitophagy |
| *RAB5C* | ENSG00000108774 | L1880|RAB5CL|RAB5L|RABL | 17 | 40276994 | 40307035 | Mitophagy core | RAB5C, member RAS oncogene family | Nat Commun. 2017 Jan 30;8:14050. A Rab5 endosomal pathway mediates Parkin-dependent mitochondrial clearance. Hammerling BC, Najor RH, Cortez MQ, Shires SE, Leon LJ, Gonzalez ER, Boassa D, Phan S, Thor A, Jimenez RE, Li H, Kitsis RN, Dorn GW II, Sadoshima J, Ellisman MH, Gustafsson ÅB. | Mitophagy |
| *RAB7A* | ENSG00000075785 | CMT2B|PRO2706|RAB7 | 3 | 128444965 | 128533639 | Mitophagy core | Ras-Associated Protein RAB7 | Sci Adv. 2018 Nov 21;4(11):eaav0443. RAB7A phosphorylation by TBK1 promotes mitophagy via the PINK-PARKIN pathway. Heo JM, Ordureau A, Swarup S, Paulo JA, Shen K, Sabatini DM, Harper JW. | Mitophagy |
| *FLCN* | ENSG00000154803 | BHD|DENND8B|FLCL | 17 | 17115526 | 17140502 | Mitophagy core | Folliculin | Sci Adv. 2018 Nov 21;4(11):eaav0443. RAB7A phosphorylation by TBK1 promotes mitophagy via the PINK-PARKIN pathway. Heo JM, Ordureau A, Swarup S, Paulo JA, Shen K, Sabatini DM, Harper JW. | Mitophagy |
| *FNIP1* | ENSG00000217128 | - | 5 | 130977407 | 131132710 | Mitophagy core | Folliculin Interacting Protein 1 | Sci Adv. 2018 Nov 21;4(11):eaav0443. RAB7A phosphorylation by TBK1 promotes mitophagy via the PINK-PARKIN pathway. Heo JM, Ordureau A, Swarup S, Paulo JA, Shen K, Sabatini DM, Harper JW. | Mitophagy |
| *NIPSNAP1* | ENSG00000184117 | - | 22 | 29950797 | 29977326 | Mitophagy core | Nipsnap Homolog 1 | Dev Cell. 2019 May 20;49(4):509-525.e12. NIPSNAP1 and NIPSNAP2 Act as .Eat Me. Signals for Mitophagy. Princely Abudu Y, Pankiv S, Mathai BJ, Håkon Lystad A, Bindesbøll C, Brenne HB, Yoke Wui Ng M, Thiede B, Yamamoto A, Mutugi Nthiga T, Lamark T, Esguerra CV, Johansen T, Simonsen A. | Mitophagy |
| *NIPSNAP2* | ENSG00000146729 | GBAS | 7 | 56019486 | 56067874 | Mitophagy core | Nipsnap Homolog 2 | Dev Cell. 2019 May 20;49(4):509-525.e12. NIPSNAP1 and NIPSNAP2 Act as .Eat Me. Signals for Mitophagy. Princely Abudu Y, Pankiv S, Mathai BJ, Håkon Lystad A, Bindesbøll C, Brenne HB, Yoke Wui Ng M, Thiede B, Yamamoto A, Mutugi Nthiga T, Lamark T, Esguerra CV, Johansen T, Simonsen A. | Mitophagy |
| *SESN2* | ENSG00000130766|ENSG00000285069 | HI95|SES2|SEST2 | 1 | 28586038 | 28609002 | Mitophagy core | sestrin 2 | Sci Rep. 2018 Jan 12;8(1):615. SESN2 facilitates mitophagy by helping Parkin translocation through ULK1 mediated Beclin1 phosphorylation. Kumar A, Shaha C. | Mitophagy |
| *RB1CC1* | ENSG00000023287 | ATG17|CC1|FIP200|PPP1R131 | 8 | 53535016 | 53658403 | Mitophagy core | RB1-inducible coiled-coil 1 | Mol Cell. 2019 Apr 18;74(2):347-362.e6. Spatiotemporal Control of ULK1 Activation by NDP52 and TBK1 during Selective Autophagy. Vargas JNS, Wang C, Bunker E, Hao L, Maric D, Schiavo G, Randow F, Youle RJ. | Mitophagy |
| *ULK1* | ENSG00000177169 | ATG1|ATG1A|UNC51|Unc51.1|hATG1 | 12 | 132379196 | 132407712 | Mitophagy core | unc-51 like autophagy activating kinase 1 | Mol Cell. 2019 Apr 18;74(2):347-362.e6. Spatiotemporal Control of ULK1 Activation by NDP52 and TBK1 during Selective Autophagy. Vargas JNS, Wang C, Bunker E, Hao L, Maric D, Schiavo G, Randow F, Youle RJ. | Mitophagy |
| *SQSTM1* | ENSG00000161011|ENSG00000284099 | A170|DMRV|FTDALS3|NADGP|OSIL|PDB3|ZIP3|p60|p62|p62B | 5 | 179233388 | 179265078 | Mitophagy-specific cargo receptors | sequestosome 1 | Nat Cell Biol. 2010 Feb;12(2):119-31. PINK1/Parkin-mediated mitophagy is dependent on VDAC1 and p62/SQSTM1. Geisler S, Holmström KM, Skujat D, Fiesel FC, Rothfuss OC, Kahle PJ, Springer W. | Mitophagy |
| *BNIP3L* | ENSG00000104765 | BNIP3a|NIX | 8 | 26240414 | 26363152 | Mitophagy-specific cargo receptors | BCL2/adenovirus E1B 19kDa interacting protein 3-like | Mol Cell. 2014 Jan 23;53(2):167-78. Interactions between autophagy receptors and ubiquitin-like proteins form the molecular basis for selective autophagy. Rogov V, Dötsch V, Johansen T, Kirkin V. | Mitophagy |
| *FUNDC1* | ENSG00000069509 | - | X | 44382885 | 44402247 | Mitophagy-specific cargo receptors | FUN14 domain containing 1 | Mol Cell. 2014 Jan 23;53(2):167-78. Interactions between autophagy receptors and ubiquitin-like proteins form the molecular basis for selective autophagy. Rogov V, Dötsch V, Johansen T, Kirkin V. | Mitophagy |
| *OPTN* | ENSG00000123240 | ALS12|FIP2|GLC1E|HIP7|HYPL|NRP|TFIIIA-INTP | 10 | 13141449 | 13180291 | Mitophagy-specific cargo receptors | optineurin | Proc Natl Acad Sci U S A. 2016 Apr 12;113(15):4039-44. Phosphorylation of OPTN by TBK1 enhances its binding to Ub chains and promotes selective autophagy of damaged mitochondria. Richter B, Sliter DA, Herhaus L, Stolz A, Wang C, Beli P, Zaffagnini G, Wild P, Martens S, Wagner SA, Youle RJ, Dikic I. | Mitophagy |
| *CALCOCO2* | ENSG00000136436 | NDP52 | 17 | 46908350 | 46943884 | Mitophagy-specific cargo receptors | calcium binding and coiled-coil domain 2 | Nature. 2015 Aug 20;524(7565):309-314. The ubiquitin kinase PINK1 recruits autophagy receptors to induce mitophagy. Lazarou M, Sliter DA, Kane LA, Sarraf SA, Wang C, Burman JL, Sideris DP, Fogel AI, Youle RJ. | Mitophagy |
| *PHB* | ENSG00000167085 | HEL-215|HEL-S-54e|PHB1 | 17 | 47481414 | 47492246 | Mitophagy-specific cargo receptors | prohibitin | Cell. 2017 Jan 12;168(1-2):224-238.e10. Prohibitin 2 Is an Inner Mitochondrial Membrane Mitophagy Receptor. Wei Y, Chiang WC, Sumpter R Jr, Mishra P, Levine B. | Mitophagy |
| *PHB2* | ENSG00000215021 | BAP|BCAP37|Bap37|PNAS-141|REA|hBAP|p22 | 12 | 7074490 | 7079988 | Mitophagy-specific cargo receptors | prohibitin 2 | Cell. 2017 Jan 12;168(1-2):224-238.e10. Prohibitin 2 Is an Inner Mitochondrial Membrane Mitophagy Receptor. Wei Y, Chiang WC, Sumpter R Jr, Mishra P, Levine B. | Mitophagy |
| *TAX1BP1* | ENSG00000106052 | CALCOCO3|T6BP|TXBP151 | 7 | 27778950 | 27884183 | Mitophagy-specific cargo receptors | Tax1 (human T-cell leukemia virus type I) binding protein 1 | Nature. 2015 Aug 20;524(7565):309-314. The ubiquitin kinase PINK1 recruits autophagy receptors to induce mitophagy. Lazarou M, Sliter DA, Kane LA, Sarraf SA, Wang C, Burman JL, Sideris DP, Fogel AI, Youle RJ. | Mitophagy |
| *LRPPRC* | ENSG00000138095 | CLONE-23970|GP130|LRP130|LSFC | 2 | 44113647 | 44223144 | Negative regulator of Mitophagy | leucine-rich pentatricopeptide repeat containing | PLoS One. 2014 Apr 10;9(4):e94903. Autophagy inhibitor LRPPRC suppresses mitophagy through interaction with mitophagy initiator Parkin. Zou J, Yue F, Li W, Song K, Jiang X, Yi J, Liu L. | Mitophagy |
| *USP30* | ENSG00000135093 | - | 12 | 109460894 | 109525831 | Negative regulator of Mitophagy | ubiquitin specific peptidase 30 | Nature. 2014 Jun 19;510(7505):370-5. The mitochondrial deubiquitinase USP30 opposes parkin-mediated mitophagy. Bingol B, Tea JS, Phu L, Reichelt M, Bakalarski CE, Song Q, Foreman O, Kirkpatrick DS, Sheng M. | Mitophagy |
| *USP35* | ENSG00000118369 | - | 11 | 77899858 | 77925757 | Negative regulator of Mitophagy | ubiquitin specific peptidase 35 | Autophagy. 2015 Apr 3;11(4):595–606. Deubiquitinating enzymes regulate PARK2-mediated mitophagy. Wang Y, Serricchio M, Jauregui M, Shanbhag R, Stoltz T, Di Paolo CT, Kim PK, McQuibban GA. | Mitophagy |
| *USP15* | ENSG00000135655 | UNPH-2|UNPH4 | 12 | 62654119 | 62811211 | Negative regulator of Mitophagy | ubiquitin specific peptidase 15 | Hum Mol Genet. 2014 Oct 1;23(19):5227-42. The deubiquitinase USP15 antagonizes Parkin-mediated mitochondrial ubiquitination and mitophagy. Cornelissen T, Haddad D, Wauters F, Van Humbeeck C, Mandemakers W, Koentjoro B, Sue C, Gevaert K, De Strooper B, Verstreken P, Vandenberghe W. | Mitophagy |
| *TMEM173* | ENSG00000184584|ENSG00000288243 | MITA|STING1 | 5 | 138855119 | 138862520 | Negative regulator of Mitophagy | Transmembrane Protein 173 | Cell Signal. 2017 Jul;35:73-83. MITA modulated autophagy flux promotes cell death in breast cancer cells. Bhatelia K, Singh K, Prajapati P, Sripada L, Roy M, Singh R. | Mitophagy |
| *SIAH3* | ENSG00000215475 | - | 13 | 46354405 | 46425871 | Negative regulator of Mitophagy | Siah E3 Ubiquitin Protein Ligase Family Member 3 | Redox Biol. 2015;4:6–13. Mitochondrial dynamics and mitochondrial quality control. Ni HM, Williams JA, Ding WX. | Mitophagy |
| *BCL2L1* | ENSG00000171552 | BCL-XL/S|BCL2L|BCLX|Bcl-X|PPP1R52 | 20 | 30252255 | 30311792 | Negative regulator of Mitophagy | BCL2-like 1 | Mol Cell. 2014 Aug 7;55(3):451-66. Bcl-2 family proteins participate in mitochondrial quality control by regulating Parkin/PINK1-dependent mitophagy. Hollville E, Carroll RG, Cullen SP, Martin SJ. | Mitophagy |
| *MCL1* | ENSG00000143384 | BCL2L3|EAT|MCL1-ES|MCL1L|MCL1S|Mcl-1|TM|bcl2-L-3|mcl1/EAT | 1 | 150547032 | 150552066 | Negative regulator of Mitophagy | Myeloid Cell Leukemia 1 | Mol Cell. 2014 Aug 7;55(3):451-66. Bcl-2 family proteins participate in mitochondrial quality control by regulating Parkin/PINK1-dependent mitophagy. Hollville E, Carroll RG, Cullen SP, Martin SJ. | Mitophagy |
| *LRRK2* | ENSG00000188906 | AURA17|DARDARIN|PARK8|RIPK7|ROCO2 | 12 | 40590546 | 40763087 | Negative regulator of Mitophagy | Leucine Rich Repeat Kinase 2 | Hum Mol Genet. 2019 May 15;28(10):1645-1660. LRRK2 impairs PINK1/Parkin-dependent mitophagy via its kinase activity: pathologic insights into Parkinson's disease. Bonello F, Hassoun SM, Mouton-Liger F, Shin YS, Muscat A, Tesson C, Lesage S, Beart PM, Brice A, Krupp J, Corvol JC, Corti O. | Mitophagy |
| *ZNF746* | ENSG00000181220 | PARIS | 7 | 149169885 | 149194908 | Negative regulator of PGC1alpha | zinc finger protein 746 | J Neurosci. 2015 Sep 16;35(37):12833–12844. Mitochondrial Quality Control via the PGC1α-TFEB Signaling Pathway Is Compromised by Parkin Q311X Mutation But Independently Restored by Rapamycin. Siddiqui A, Bhaumik D, Chinta SJ, Rane A, Rajagopalan S, Lieu CA, Lithgow GJ, Andersen JK. | Mitophagy |
| *MFN1* | ENSG00000171109 | hfzo1|hfzo2 | 3 | 179065480 | 179112719 | Fusion | mitofusin 1 | J Cell Biol. 2003 Jan 20;160(2):189-200. Epub 2003 Jan 13. Mitofusins Mfn1 and Mfn2 coordinately regulate mitochondrial fusion and are essential for embryonic development. Chen H, Detmer SA, Ewald AJ, Griffin EE, Fraser SE, Chan DC. | Mitophagy |
| *MFN2* | ENSG00000116688 | CMT2A|CMT2A2|CMT2A2A|CMT2A2B|CPRP1|HMSN6A|HSG|MARF | 1 | 12040238 | 12073571 | Fusion | mitofusin 2 | J Cell Biol. 2003 Jan 20;160(2):189-200. Epub 2003 Jan 13. Mitofusins Mfn1 and Mfn2 coordinately regulate mitochondrial fusion and are essential for embryonic development. Chen H, Detmer SA, Ewald AJ, Griffin EE, Fraser SE, Chan DC. | Mitophagy |
| *OPA1* | ENSG00000198836 | BERHS|MGM1|MTDPS14|NPG|NTG|largeG | 3 | 193310933 | 193415612 | Fusion | optic atrophy 1 (autosomal dominant) | Proc Natl Acad Sci U S A. 2004 Nov 9;101(45):15927-32. OPA1 requires mitofusin 1 to promote mitochondrial fusion. Cipolat S, Martins de Brito O, Dal Zilio B, Scorrano L. | Mitophagy |
| *ROCK1* | ENSG00000067900 | P160ROCK|ROCK-I | 18 | 18526867 | 18691812 | Fission | Rho-associated, coiled-coil containing protein kinase 1 | Cell Metab. 2012 Feb 8;15(2):186-200. Mitochondrial fission triggered by hyperglycemia is mediated by ROCK1 activation in podocytes and endothelial cells. Wang W, Wang Y, Long J, Wang J, Haudek SB, Overbeek P, Chang BH, Schumacker PT, Danesh FR. | Mitophagy |
| *MIEF1* | ENSG00000100335 | AltMIEF1|HSU79252|MID51|MIEF1-MP|SMCR7L|dJ1104E15.3 | 22 | 39895437 | 39914137 | Fission | Mitochondrial Elongation Factor 1 | Mol Biol Cell. 2013 Mar;24(5):659-67. Fis1, Mff, MiD49, and MiD51 mediate Drp1 recruitment in mitochondrial fission. Losón OC, Song Z, Chen H, Chan DC. | Mitophagy |
| *MIEF2* | ENSG00000177427|ENSG00000284495 | MID49|SMCR7 | 17 | 18163848 | 18169866 | Fission | Mitochondrial Elongation Factor 2 | Mol Biol Cell. 2013 Mar;24(5):659-67. Fis1, Mff, MiD49, and MiD51 mediate Drp1 recruitment in mitochondrial fission. Losón OC, Song Z, Chen H, Chan DC. | Mitophagy |
| *DNM1L* | ENSG00000087470 | DLP1|DRP1|DVLP|DYMPLE|EMPF|EMPF1|HDYNIV|OPA5 | 12 | 32832134 | 32898486 | Fission | dynamin 1-like | J Cell Biol. 2016 Feb 15;212(4):379–387. Metabolic regulation of mitochondrial dynamics. Mishra P, Chan DC. | Mitophagy |
| *FIS1* | ENSG00000214253 | CGI-135|TTC11 | 7 | 100882739 | 100895597 | Fission | Fission, Mitochondrial 1 | Mol Biol Cell. 2013 Mar;24(5):659-67. Fis1, Mff, MiD49, and MiD51 mediate Drp1 recruitment in mitochondrial fission. Losón OC, Song Z, Chen H, Chan DC. | Mitophagy |
| *HTRA2* | ENSG00000115317 | MGCA8|OMI|PARK13|PRSS25 | 2 | 74756504 | 74760472 | Fission | HtrA serine peptidase 2 | Exp Cell Res. 2010 Apr 15;316(7):1213-24. Modulation of mitochondrial function and morphology by interaction of Omi/HtrA2 with the mitochondrial fusion factor OPA1. Kieper N, Holmström KM, Ciceri D, Fiesel FC, Wolburg H, Ziviani E, Whitworth AJ, Martins LM, Kahle PJ, Krüger R. | Mitophagy |
| *MFF* | ENSG00000168958 | C2orf33|EMPF2|GL004 | 2 | 228189867 | 228222550 | Fission | mitochondrial fission factor | Mol Biol Cell. 2013 Mar;24(5):659-67. Fis1, Mff, MiD49, and MiD51 mediate Drp1 recruitment in mitochondrial fission. Losón OC, Song Z, Chen H, Chan DC. | Mitophagy |
| *SIRT2* | ENSG00000068903|ENSG00000283100 | SIR2|SIR2L|SIR2L2 | 19 | 39369197 | 39390502 | Transcription Factors involved in Mitophagy | sirtuin 2 | 1)Biochem Biophys Res Commun. 2017 Jul 8;488(4):603-608. Sirtuin inhibition leads to autophagy and apoptosis in porcine preimplantation blastocysts. Kim MG; 2) Mol Neurobiol. 2017 Aug;54(6):4021-4040. Mitochondrial Metabolism Power SIRT2-Dependent Deficient Traffic Causing Alzheimer's-Disease Related Pathology. Silva DF | Mitophagy |
| *SIRT3* | ENSG00000142082 | SIR2L3 | 11 | 215458 | 236931 | Transcription Factors involved in Mitophagy | sirtuin 3 | Biochim Biophys Acta. 2017 Aug;1863(8):1973-1983. Sirt3 deficiency exacerbates diabetic cardiac dysfunction: Role of Foxo3A-Parkin-mediated mitophagy. Yu W | Mitophagy |
| *SIRT5* | ENSG00000124523 | SIR2L5 | 6 | 13574816 | 13614790 | Transcription Factors involved in Mitophagy | sirtuin 5 | Autophagy. 2015;11(2):253-70. SIRT5 regulation of ammonia-induced autophagy and mitophagy. Polletta L | Mitophagy |
| *SOX2* | ENSG00000181449 | ANOP3|MCOPS3 | 3 | 181429714 | 181432221 | Transcription Factors involved in Mitophagy | SRY-box 2 | Autophagy. 2017 Jul 19:0. BNIP3L-dependent Mitophagy Accounts for Mitochondrial Clearance during Three Factors Induced Somatic Cell Reprogramming. Xiang G | Mitophagy |
| *MITF* | ENSG00000187098 | CMM8|COMMAD|MI|WS2|WS2A|bHLHe32 | 3 | 69788586 | 70017488 | Transcription Factors involved in Mitophagy | melanogenesis associated transcription factor | J Cell Biol. 2015 Aug 3;210(3):435-50. MiT/TFE transcription factors are activated during mitophagy downstream of Parkin and Atg5. Nezich CL, Wang C, Fogel AI, Youle RJ. | Mitophagy |
| *TFE3* | ENSG00000068323 | RCCP2|RCCX1|TFEA|bHLHe33 | X | 48886242 | 48901012 | Transcription Factors involved in Mitophagy | transcription factor binding to IGHM enhancer 3 [Homo sapiens | J Cell Biol. 2015 Aug 3;210(3):435-50. MiT/TFE transcription factors are activated during mitophagy downstream of Parkin and Atg5. Nezich CL, Wang C, Fogel AI, Youle RJ. | Mitophagy |
| *TFEB* | ENSG00000112561 | ALPHATFEB|BHLHE35|TCFEB | 6 | 41651716 | 41703997 | Transcription Factors involved in Mitophagy | transcription factor EB | J Cell Biol. 2015 Aug 3;210(3):435-50. MiT/TFE transcription factors are activated during mitophagy downstream of Parkin and Atg5. Nezich CL, Wang C, Fogel AI, Youle RJ. | Mitophagy |
| *TFEC* | ENSG00000105967 | TCFEC|TFE-C|TFEC-L|TFECL|bHLHe34|hTFEC-L | 7 | 115575202 | 115799950 | Transcription Factors involved in Mitophagy | transcription factor EC | Pharmacol Res. 2015 Sep;99:36-43. The MITF family of transcription factors: Role in endolysosomal biogenesis, Wnt signaling, and oncogenesis. Ploper D | Mitophagy |
| *SREBF1* | ENSG00000072310 | SREBP1|bHLHd1 | 17 | 17713713 | 17740325 | Transcription Factors involved in Mitophagy | sterol regulatory element binding transcription factor 1 | Autophagy. 2014 Aug;10(8):1476-7. SREBF1 links lipogenesis to mitophagy and sporadic Parkinson disease. Ivatt RM | Mitophagy |
| *SREBF2* | ENSG00000198911 | SREBP-2|SREBP2|bHLHd2 | 22 | 42229109 | 42303312 | Transcription Factors involved in Mitophagy | sterol regulatory element binding transcription factor 2 | Int J Biochem Cell Biol. 2014 Oct;55:196-208. Leishmania donovani activates SREBP2 to modulate macrophage membrane cholesterol and mitochondrial oxidants for establishment of infection. Mukherjee M | Mitophagy |
| *VAMP3* | ENSG00000049245 | CEB | 1 | 7831329 | 7841492 | SNAREs | Vesicle Associated Membrane Protein 3 | Biochim Biophys Acta. 2009 Dec;1793(12):1901–1916TI-VAMP/VAMP7 and VAMP3/cellubrevin: two v-SNARE proteins involved in specific steps of the autophagy/multivesicular body pathways. Fader CM, Sánchez DG, Mestre MB, Colombo MI. | Docking and fusion |
| *STX17* | ENSG00000136874 | - | 9 | 102668915 | 102732618 | SNAREs | Syntaxin 17 | .Cell. 2012 Dec 7;151(6):1256–1269The hairpin-type tail-anchored SNARE syntaxin 17 targets to autophagosomes for fusion with endosomes/lysosomes. Itakura E, Kishi-Itakura C, Mizushima N. | Docking and fusion |
| *YKT6* | ENSG00000106636 | - | 7 | 44240567 | 44253893 | SNAREs | Synaptobrevin homolog YKT6 | Cell. 2011 Jul 22;146(2):290–302SNARE proteins are required for macroautophagy. Nair U, Jotwani A, Geng J, Gammoh N, Richerson D, Yen WL, Griffith J, Nag S, Wang K, Moss T, Baba M, McNew JA, Jiang X, Reggiori F, Melia TJ, Klionsky DJ. | Docking and fusion |
| *VTI1A* | ENSG00000151532 | MMDS3|MVti1|VTI1RP2|Vti1-rp2 | 10 | 114206756 | 114578503 | SNAREs | Vesicle Transport Through Interaction With T-SNAREs 1A | Mol Cell. 2013 Oct 24;52(2):264–271Syntaxin 13, a genetic modifier of mutant CHMP2B in frontotemporal dementia, is required for autophagosome maturation. Lu Y, Zhang Z, Sun D, Sweeney ST, Gao FB. | Docking and fusion |
| *VAMP7* | ENSG00000124333 | SYBL1|TI-VAMP|TIVAMP|VAMP-7 | X | 155110956 | 155173433 | SNAREs | Vesicle Associated Membrane Protein 7 | [Biochim Biophys Acta. 2009 Dec;1793(12):1901–1916TI-VAMP/VAMP7 and VAMP3/cellubrevin: two v-SNARE proteins involved in specific steps of the autophagy/multivesicular body pathways. Fader CM, Sánchez DG, Mestre MB, Colombo MI.](https://www.ncbi.nlm.nih.gov/pubmed/19781582) | Docking and fusion |
| *SNAP29* | ENSG00000099940 | CEDNIK|SNAP-29 | 22 | 21213271 | 21245506 | SNAREs | Synaptosome Associated Protein 29 | J Mol Biol. 2019 Nov 2. pii: S0022-2836(19)30624-2. Autophagosome-Lysosome Fusion. Lőrincz P, Juhász G. | Docking and fusion |
| *VPS33A* | ENSG00000139719 | MPSPS | 12 | 122714111 | 122751068 | HOPS | Vacuolar Protein Sorting-Associated Protein 33A | Mol Biol Cell. 2014 Apr;25(8):1327–1337The HOPS complex mediates autophagosome-lysosome fusion through interaction with syntaxin 17. Jiang P, Nishimura T, Sakamaki Y, Itakura E, Hatta T, Natsume T, Mizushima N. | Docking and fusion |
| *RAB7A* | ENSG00000075785 | CMT2B|PRO2706|RAB7 | 3 | 128444965 | 128533639 | HOPS | Ras-Associated Protein RAB7 | [J Cell Sci. 2015 May 1;128(9):1696–1706HBV secretion is regulated through the activation of endocytic and autophagic compartments mediated by Rab7 stimulation. Inoue J, Krueger EW, Chen J, Cao H, Ninomiya M, McNiven MA.](https://www.ncbi.nlm.nih.gov/pubmed/25770103) | Docking and fusion |
| *VPS41* | ENSG00000006715 | HVPS41|HVSP41|hVps41p | 7 | 38762563 | 38971994 | HOPS | Vacuolar Protein Sorting-Associated Protein 41 Homolog | Traffic. 2015 Jul;16(7):727–742Recruitment of VPS33A to HOPS by VPS16 Is Required for Lysosome Fusion with Endosomes and Autophagosomes. Wartosch L, Günesdogan U, Graham SC, Luzio JP. | Docking and fusion |
| *VPS11* | ENSG00000160695|ENSG00000280616 | END1|HLD12|PEP5|RNF108|hVPS11 | 11 | 118938403 | 118952688 | HOPS | Vacuolar Protein Sorting-Associated Protein 11 Homolog | Traffic. 2015 Jul;16(7):727–742Recruitment of VPS33A to HOPS by VPS16 Is Required for Lysosome Fusion with Endosomes and Autophagosomes. Wartosch L, Günesdogan U, Graham SC, Luzio JP. | Docking and fusion |
| *VPS16* | ENSG00000215305 | hVPS16 | 20 | 2821349 | 2847378 | HOPS | Vacuolar Protein Sorting-Associated Protein 16 Homolog | [Traffic. 2015 Jul;16(7):727–742Recruitment of VPS33A to HOPS by VPS16 Is Required for Lysosome Fusion with Endosomes and Autophagosomes. Wartosch L, Günesdogan U, Graham SC, Luzio JP.](https://www.ncbi.nlm.nih.gov/pubmed/25783203) | Docking and fusion |
| *VPS39* | ENSG00000166887 | TLP|VAM6|hVam6p | 15 | 42450899 | 42500514 | HOPS | Vacuolar Protein Sorting 39 Homolog | [Traffic. 2015 Jul;16(7):727–742Recruitment of VPS33A to HOPS by VPS16 Is Required for Lysosome Fusion with Endosomes and Autophagosomes. Wartosch L, Günesdogan U, Graham SC, Luzio JP.](https://www.ncbi.nlm.nih.gov/pubmed/25783203) | Docking and fusion |
| *VPS18* | ENSG00000104142 | PEP3 | 15 | 41186628 | 41196173 | HOPS | Vacuolar Protein Sorting Protein 18 | [Traffic. 2015 Jul;16(7):727–742Recruitment of VPS33A to HOPS by VPS16 Is Required for Lysosome Fusion with Endosomes and Autophagosomes. Wartosch L, Günesdogan U, Graham SC, Luzio JP.](https://www.ncbi.nlm.nih.gov/pubmed/25783203) | Docking and fusion |
| *EPG5* | ENSG00000152223 | HEEW1|KIAA1632|VICIS | 18 | 43427574 | 43547240 | Tethers, Adaptors | Ectopic P-Granules Autophagy Protein 5 Homolog | J Mol Biol. 2019 Nov 2. pii: S0022-2836(19)30624-2. Autophagosome-Lysosome Fusion. Lőrincz P, Juhász G. | Docking and fusion |
| *ATG14* | ENSG00000126775 | ATG14L|BARKOR|KIAA0831 | 14 | 55833110 | 55878576 | Tethers, Adaptors | autophagy related 14 | J Mol Biol. 2019 Nov 2. pii: S0022-2836(19)30624-2. Autophagosome-Lysosome Fusion. Lőrincz P, Juhász G. | Docking and fusion |
| *TECPR1* | ENSG00000205356 | - | 7 | 97843936 | 97881563 | Tethers, Adaptors | Tectonin Beta-Propeller Repeat Containing 1 | J Mol Biol. 2019 Nov 2. pii: S0022-2836(19)30624-2. Autophagosome-Lysosome Fusion. Lőrincz P, Juhász G. | Docking and fusion |
| *GORASP2* | ENSG00000115806 | GOLPH6|GRASP55|GRS2|p59 | 2 | 171784974 | 171823639 | Tethers, Adaptors | Golgi Reassembly Stacking Protein 2 | J Mol Biol. 2019 Nov 2. pii: S0022-2836(19)30624-2. Autophagosome-Lysosome Fusion. Lőrincz P, Juhász G. | Docking and fusion |
| *BIRC6* | ENSG00000115760 | APOLLON|BRUCE | 2 | 32582096 | 32843966 | Tethers, Adaptors | Baculoviral IAP Repeat Containing 6 | J Mol Biol. 2019 Nov 2. pii: S0022-2836(19)30624-2. Autophagosome-Lysosome Fusion. Lőrincz P, Juhász G. | Docking and fusion |
| *RUFY4* | ENSG00000188282 | ZFYVE31 | 2 | 218899683 | 218955304 | Tethers, Adaptors | RUN And FYVE Domain Containing 4 | J Mol Biol. 2019 Nov 2. pii: S0022-2836(19)30624-2. Autophagosome-Lysosome Fusion. Lőrincz P, Juhász G. | Docking and fusion |
| *ARL8B* | ENSG00000134108 | ARL10C|Gie1 | 3 | 5163905 | 5222596 | recruitment HOPS on lysosome | ADP Ribosylation Factor Like GTPase 8B | [Traffic. 2012 Dec;13(12):1667–1679Rab7 and Arl8 GTPases are necessary for lysosome tubulation in macrophages. Mrakovic A, Kay JG, Furuya W, Brumell JH, Botelho RJ.](https://www.ncbi.nlm.nih.gov/pubmed/22909026) | Docking and fusion |
| *RAB20* | ENSG00000139832 | - | 13 | 111175417 | 111214080 | Autophagosome-lysosome fusion | RAB20, Member RAS Oncogene Family | Nanoscale. 2017 Mar 2;9(9):3269–3282Systematic investigation on the intracellular trafficking network of polymeric nanoparticles. Zhang J, Chang D, Yang Y, Zhang X, Tao W, Jiang L, Liang X, Tsai H, Huang L, Mei L. | Docking and fusion |
| *RAB39A* | ENSG00000179331 | RAB39 | 11 | 107799229 | 107834208 | Autophagosome-lysosome fusion | RAB39A, Member RAS Oncogene Family | PLoS One. 2013 Dec 13;8(12):e83324Rab39a interacts with phosphatidylinositol 3-kinase and negatively regulates autophagy induced by lipopolysaccharide stimulation in macrophages. Seto S, Sugaya K, Tsujimura K, Nagata T, Horii T, Koide Y. | Docking and fusion |
| *CTSB* | ENSG00000164733|ENSG00000285132 | APPS|CPSB|RECEUP | 8 | 11700033 | 11726957 | Lysosomal cysteine proteinases | cathepsin B | Brix, K. (2005) Lysosomal proteases. Revival of the Sleeping Beauty. in Lysosomes (Saftig, P., ed) pp. 50 –59, Springer, New York | Lysosome |
| *CTSK* | ENSG00000143387 | CTS02|CTSO|CTSO1|CTSO2|PKND|PYCD | 1 | 150768684 | 150780799 | Lysosomal endopeptidases | cathepsin K | Brix, K. (2005) Lysosomal proteases. Revival of the Sleeping Beauty. in Lysosomes (Saftig, P., ed) pp. 50 –59, Springer, New York | Lysosome |
| *CTSS* | ENSG00000163131 | - | 1 | 150702672 | 150738433 | lysosomal cysteine proteinase; Cat S Deficiency Increases the Accumulation of Autophagosomes in Macrophages | cathepsin S | PLoS One. 2012;7(4):e35315. Cathepsin S deficiency results in abnormal accumulation of autophagosomes in macrophages and enhances Ang II-induced cardiac inflammation. Pan L | Lysosome |
| *PPT1* | ENSG00000131238 | CLN1|INCL|PPT | 1 | 40538379 | 40563375 | lysosomal hydrolase; Mutations in palmitoyl protein thioesterase-1 (PPT1) have been found to cause the infantile form of neuronal ceroid lipofuscinosis, which is a lysosomal storage disorder | palmitoyl-protein thioesterase 1 | EMBO J. 1996 Oct 1;15(19):5240-5. Human palmitoyl protein thioesterase: evidence for lysosomal targeting of the enzyme and disturbed cellular routing in infantile neuronal ceroid lipofuscinosis. Hellsten E | Lysosome |
| *ASAH1* | ENSG00000104763 | AC|ACDase|ASAH|PHP|PHP32|SMAPME | 8 | 17913934 | 17942494 | lysosomal lumen | N-acylsphingosine amidohydrolase (acid ceramidase) 1 |  | Lysosome |
| *CD1E* | ENSG00000158488 | CD1A|R2 | 1 | 158323254 | 158327343 | lysosomal lumen | CD1e molecule |  | Lysosome |
| *GALNS* | ENSG00000141012 | GALNAC6S|GAS|GalN6S|MPS4A | 16 | 88880142 | 88923378 | lysosomal lumen | galactosamine (N-acetyl)-6-sulfate sulfatase |  | Lysosome |
| *GLB1* | ENSG00000170266 | EBP|ELNR1|MPS4B | 3 | 33038100 | 33138722 | lysosomal lumen | galactosidase, beta 1 |  | Lysosome |
| *GM2A* | ENSG00000196743 | GM2-AP|SAP-3 | 5 | 150591711 | 150650001 | lysosomal lumen | GM2 ganglioside activator |  | Lysosome |
| *GNS* | ENSG00000135677 | G6S | 12 | 65107225 | 65153227 | lysosomal lumen | glucosamine (N-acetyl)-6-sulfatase |  | Lysosome |
| *GUSB* | ENSG00000169919 | BG|MPS7 | 7 | 65425671 | 65447301 | lysosomal lumen | glucuronidase, beta |  | Lysosome |
| *HEXA* | ENSG00000213614 | TSD | 15 | 72635775 | 72668817 | lysosomal lumen | hexosaminidase A (alpha polypeptide) |  | Lysosome |
| *HEXB* | ENSG00000049860 | ENC-1AS|HEL-248|HEL-S-111 | 5 | 73935848 | 74018472 | Catabolic enzyme | hexosaminidase B (beta polypeptide) |  | Lysosome |
| *IDS* | ENSG00000010404 | ID2S|MPS2|SIDS | X | 148558521 | 148615470 | lysosomal lumen | iduronate 2-sulfatase |  | Lysosome |
| *LGMN* | ENSG00000100600 | AEP|LGMN1|PRSC1 | 14 | 93170152 | 93215047 | lysosomal cysteine proteinase: it mediates the biosynthetic processing of the ubiquitously expressed cathepsins B, H, and L which also belong to the family of lysosomal cysteine proteinases. | legumain | Brix, K. (2005) Lysosomal proteases. Revival of the Sleeping Beauty. in Lysosomes (Saftig, P., ed) pp. 50 –59, Springer, New York | Lysosome |
| *PLBD2* | ENSG00000151176 | P76 | 12 | 113796371 | 113827203 | lysosomal phospholipase | phospholipase B domain containing 2 | Biochem J. 2007 Mar 15;402(3):449-58. Biochemical characterization and lysosomal localization of the mannose-6-phosphate protein p76 (hypothetical protein LOC196463). Jensen AG | Lysosome |
| *SGSH* | ENSG00000181523 | HSS|MPS3A|SFMD | 17 | 78180515 | 78194722 | lysosomal lumen | N-sulfoglucosamine sulfohydrolase |  | Lysosome |
| *SMPD1* | ENSG00000166311 | ASM|ASMASE|NPD | 11 | 6411655 | 6416228 | lysosomal lumen | sphingomyelin phosphodiesterase 1, acid lysosomal |  | Lysosome |
| *ABCA2* | ENSG00000107331 | ABC2|IDPOGSA | 9 | 139901686 | 139923367 | ATP-binding cassette (ABC) transporters | ATP-binding cassette, sub-family A (ABC1), member 2 |  | Lysosome |
| *ABCA5* | ENSG00000154265 | ABC13|EST90625|HTC3 | 17 | 67240452 | 67323385 | lysosomal membrane | ATP-binding cassette, sub-family A (ABC1), member 5 |  | Lysosome |
| *ABCB9* | ENSG00000150967 | EST122234|TAPL | 12 | 123405498 | 123466196 | lysosomal membrane | ATP-binding cassette, sub-family B (MDR/TAP), member 9 |  | Lysosome |
| *AP1G1* | ENSG00000166747 | ADTG|CLAPG1 | 16 | 71762913 | 71843104 | lysosomal membrane | adaptor-related protein complex 1, gamma 1 subunit |  | Lysosome |
| *AP3B1* | ENSG00000132842 | ADTB3|ADTB3A|HPS|HPS2|PE | 5 | 77296349 | 77590579 | involved in the sorting of integral membrane proteins to lysosomes and that impairment of this process may underlie the clinical symptoms of Hermansky-Pudlak syndrome type 2 | adaptor-related protein complex 3, beta 1 subunit | Mol Cell. 1999 Jan;3(1):11-21. Altered trafficking of lysosomal proteins in Hermansky-Pudlak syndrome due to mutations in the beta 3A subunit of the AP-3 adaptor. Dell'Angelica EC | Lysosome |
| *ARL8A* | ENSG00000143862 | ARL10B|GIE2 | 1 | 202102532 | 202113869 | lysosomal membrane | ADP-ribosylation factor-like 8A |  | Lysosome |
| *ARL8B* | ENSG00000134108 | ARL10C|Gie1 | 3 | 5163905 | 5222596 | lysosomal membrane | ADP-ribosylation factor-like 8B |  | Lysosome |
| *ATP13A2* | ENSG00000159363 | CLN12|HSA9947|KRPPD|PARK9|SPG78 | 1 | 17312453 | 17338423 | lysosomal membrane | ATPase type 13A2 |  | Lysosome |
| *ATP6V0A2* | ENSG00000185344 | A2|ARCL|ARCL2A|ATP6A2|ATP6N1D|J6B7|RTF|STV1|TJ6|TJ6M|TJ6S|VPH1|WSS | 12 | 124196865 | 124246302 | lysosomal acidification | ATPase, H+ transporting, lysosomal V0 subunit a2 | 1) Hum Mol Genet. 2011 Oct 1;20(19):3852-66. Characterization of the CLEAR network reveals an integrated control of cellular clearance pathways. Palmieri M; 2) Traffic. 2007 Dec;8(12):1676-86. Integral and associated lysosomal membrane proteins. Schröder B. | Lysosome |
| *ATP6V0A4* | ENSG00000105929 | A4|ATP6N1B|ATP6N2|RDRTA2|RTA1C|RTADR|STV1|VPH1|VPP2 | 7 | 138391040 | 138484305 | regulation of the acidification of autophagosomes and autophagic flux | ATPase, H+ transporting, lysosomal V0 subunit a4 | 1) Autophagy. 2015;11(5):756-68. The integral membrane protein ITM2A, a transcriptional target of PKA-CREB, regulates autophagic flux via interaction with the vacuolar ATPase. Namkoong SPalmieri M; 2) Traffic. 2007 Dec;8(12):1676-86. Integral and associated lysosomal membrane proteins. Schröder B. | Lysosome |
| *ATP6V0C* | ENSG00000185883 | ATP6C|ATP6L|ATPL|VATL|VPPC|Vma3 | 16 | 2563871 | 2570219 | lysosomal lumen acidification | ATPase, H+ transporting, lysosomal 16kDa, V0 subunit c | 1)PLoS One. 2014 Apr 2;9(4):e93257. ATP6V0C knockdown in neuroblastoma cells alters autophagy-lysosome pathway function and metabolism of proteins that accumulate in neurodegenerative disease. Mangieri LR; 2) Traffic. 2007 Dec;8(12):1676-86. Integral and associated lysosomal membrane proteins. Schröder B. | Lysosome |
| *ATP6V0D1* | ENSG00000159720 | ATP6D|ATP6DV|P39|VATX|VMA6|VPATPD | 16 | 67471917 | 67515140 | lysosomal acidification (Palmieri_Table 1) | ATPase, H+ transporting, lysosomal 38kDa, V0 subunit d1 | 1) Hum Mol Genet. 2011 Oct 1;20(19):3852-66. Characterization of the CLEAR network reveals an integrated control of cellular clearance pathways. Palmieri M; 2) Traffic. 2007 Dec;8(12):1676-86. Integral and associated lysosomal membrane proteins. Schröder B. | Lysosome |
| *CCZ1* | ENSG00000122674 | C7orf28A|CCZ1A|CGI-43|H_DJ1163J12.2 | 7 | 5938356 | 5965605 | lysosomal membrane | CCZ1 vacuolar protein trafficking and biogenesis associated homolog (S. cerevisiae) |  | Lysosome |
| *CD1B* | ENSG00000158485 | CD1|CD1A|R1 | 1 | 158297741 | 158301321 | lysosomal membrane | CD1b molecule |  | Lysosome |
| *CD68* | ENSG00000129226 | GP110|LAMP4|SCARD1 | 17 | 7482785 | 7485429 | lysosomal membrane | CD68 molecule |  | Lysosome |
| *CLCN5* | ENSG00000171365 | CLC5|CLCK2|ClC-5|DENTS|NPHL1|NPHL2|XLRH|XRN|hCIC-K2 | X | 49687225 | 49863892 | Cl− transporter | chloride channel, voltage-sensitive 5 | J Physiol. 2015 Sep 15; 593(Pt 18): 4139–4150. A tale of two CLCs: biophysical insights toward understanding ClC-5 and ClC-7 function in endosomes and lysosomesGiovanni Zifarelli | Lysosome |
| *CLCN6* | ENSG00000011021 | CLC-6 | 1 | 11866207 | 11903201 | lysosomal membrane | chloride channel, voltage-sensitive 6 |  | Lysosome |
| *CLCN7* | ENSG00000103249 | CLC-7|CLC7|HOD|OPTA2|OPTB4|PPP1R63 | 16 | 1494935 | 1525581 | Cl− transporter | chloride channel, voltage-sensitive 7 | EMBO J. 2011 Jun 1;30(11):2140-52. ClC-7 is a slowly voltage-gated 2Cl(-)/1H(+)-exchanger and requires Ostm1 for transport activity. Leisle L; Kasper D, Planells-Cases R, Fuhrmann JC, Scheel O, Zeitz O, Ruether K, Schmitt A, Poët M, Steinfeld R, Schweizer M, Kornak U, Jentsch TJ (2005) Loss of the chloride channel ClC-7 leads to lysosomal storage disease and neurodegeneration. EMBO J 24: 1079–1091; .J Physiol. 2015 Sep 15; 593(Pt 18): 4139–4150. A tale of two CLCs: biophysical insights toward understanding ClC-5 and ClC-7 function in endosomes and lysosomesGiovanni Zifarelli. | Lysosome |
| *CLN5* | ENSG00000102805 | - | 13 | 77564795 | 77576652 | soluble lysosomal glycoprotein causes neuronal ceroid lipofuscinosis, an inherited neurodegenerative lysosomal storage disorder | ceroid-lipofuscinosis, neuronal 5 | Exp Cell Res. 2015 Oct 15;338(1):45-53. Proteolytic processing of the neuronal ceroid lipofuscinosis related lysosomal protein CLN5. De Silva B | Lysosome |
| *CTNS* | ENSG00000040531 | CTNS-LSB|PQLC4|SLC66A4 | 17 | 3539762 | 3564836 | lysosomal membrane | cystinosin, lysosomal cystine transporter |  | Lysosome |
| *DAB2* | ENSG00000153071 | DOC-2|DOC2 | 5 | 39371780 | 39462402 | lysosomal membrane | Dab, mitogen-responsive phosphoprotein, homolog 2 (Drosophila) |  | Lysosome |
| *DNAJC5* | ENSG00000101152 | CLN4|CLN4B|CSP|DNAJC5A|NCL|mir-941-2|mir-941-3|mir-941-4|mir-941-5 | 20 | 62526518 | 62567384 | lysosomal membrane | DnaJ (Hsp40) homolog, subfamily C, member 5 |  | Lysosome |
| *DRAM1* | ENSG00000136048 | DRAM | 12 | 102271129 | 102405908 | lysosomal membrane | DNA-damage regulated autophagy modulator 1 |  | Lysosome |
| *EGF* | ENSG00000138798 | HOMG4|URG | 4 | 110834040 | 110933422 | lysosomal membrane | epidermal growth factor |  | Lysosome |
| *GAA* | ENSG00000171298 | LYAG | 17 | 78075355 | 78093678 | lysosomal alpha-glucosidase, which is essential for the degradation of glycogen to glucose in lysosomes. Defects in this gene are the cause of glycogen storage disease II, also known as Pompe's disease. | glucosidase, alpha; acid | Pediatr Res. 1998 Mar;43(3):374-80. Recombinant human acid alpha-glucosidase corrects acid alpha-glucosidase-deficient human fibroblasts, quail fibroblasts, and quail myoblasts. Yang HW | Lysosome |
| *GBA* | ENSG00000177628|ENSG00000262446 | GBA1|GCB|GLUC | 1 | 155204243 | 155214490 | lysosomal membrane | glucosidase, beta, acid |  | Lysosome |
| *GPR137B* | ENSG00000077585 | TM7SF1 | 1 | 236305832 | 236385165 | lysosomal membrane | G protein-coupled receptor 137B |  | Lysosome |
| *HGSNAT* | ENSG00000165102 | HGNAT|MPS3C|RP73|TMEM76 | 8 | 42995556 | 43057998 | lysosomal membrane | heparan-alpha-glucosaminide N-acetyltransferase |  | Lysosome |
| *LAMP2* | ENSG00000005893 | CD107b|DND|LAMP-2|LAMPB|LGP-96|LGP110 | X | 119561682 | 119603220 | lysosomal membrane | LAMP2 |  | Lysosome |
| *LAMP3* | ENSG00000078081 | CD208|DC LAMP|DC-LAMP|DCLAMP|LAMP|LAMP-3|TSC403 | 3 | 182840001 | 182881627 | lysosomal membrane | lysosomal-associated membrane protein 3 |  | Lysosome |
| *LITAF* | ENSG00000189067 | PIG7|SIMPLE|TP53I7 | 16 | 11641853 | 11730237 | lysosomal membrane | lipopolysaccharide-induced TNF factor |  | Lysosome |
| *LMBRD1* | ENSG00000168216 | C6orf209|LMBD1|MAHCF|NESI | 6 | 70385694 | 70507003 | lysosomal membrane | LMBR1 domain containing 1 |  | Lysosome |
| *MCOLN1* | ENSG00000090674 | MG-2|ML1|ML4|MLIV|MST080|MSTP080|TRP-ML1|TRPM-L1|TRPML1 | 19 | 7587512 | 7598895 | lysosomal membrane | mucolipin 1 |  | Lysosome |
| *MFSD8* | ENSG00000164073 | CCMD|CLN7 | 4 | 128838960 | 128887150 | lysosomal membrane protein.Mutations in this gene are correlated with a variant form of late infantile-onset neuronal ceroid lipofuscinoses (vLINCL). | major facilitator superfamily domain containing 8 | Hum Mol Genet. 2010 Nov 15;19(22):4497-514. doi: 10.1093/hmg/ddq381. Epub 2010 Sep 7. Expression and lysosomal targeting of CLN7, a major facilitator superfamily transporter associated with variant late-infantile neuronal ceroid lipofuscinosis. Sharifi A | Lysosome |
| *MYO7A* | ENSG00000137474 | DFNA11|DFNB2|MYOVIIA|MYU7A|NSRD2|USH1B | 11 | 76839310 | 76926284 | lysosome-associated molecular motor. Mutations in the myosin-VIIa (MYO7a) gene cause human Usher disease. | myosin VIIA | Cell Motil Cytoskeleton. 2005 Sep;62(1):13-26. The unconventional myosin-VIIa associates with lysosomes. Soni LE | Lysosome |
| *NCSTN* | ENSG00000162736 | ATAG1874 | 1 | 160313062 | 160328742 | proteolysis | nicastrin | Biochem Biophys Res Commun. 2003 Jan 17;300(3):615-8. Nicastrin is a resident lysosomal membrane protein. Bagshaw RD; 2) J Biol Chem. 2003 Jul 18;278(29):26687-94. Presenilin-1, nicastrin, amyloid precursor protein, and gamma-secretase activity are co-localized in the lysosomal membrane. Pasternak SH | Lysosome |
| *NPC1* | ENSG00000141458 | NPC|POGZ|SLC65A1 | 18 | 21086148 | 21166862 | lysosomal membrane | NPC1 | Cell. 2007 Nov 16;131(4):770-83. LIMP-2 is a receptor for lysosomal mannose-6-phosphate-independent targeting of beta-glucocerebrosidase. Reczek D, Schwake M, Schröder J, Hughes H, Blanz J, Jin X, Brondyk W, Van Patten S, Edmunds T, Saftig P. | Lysosome |
| *PI4K2A* | ENSG00000155252 | PI4KII|PIK42A | 10 | 99344131 | 99436191 | lysosomal membrane | phosphatidylinositol 4-kinase type 2 alpha |  | Lysosome |
| *PLD1* | ENSG00000075651 | CVDD | 3 | 171318195 | 171528740 | lysosomal membrane | phospholipase D1, phosphatidylcholine-specific |  | Lysosome |
| *PSEN1* | ENSG00000080815 | ACNINV3|AD3|FAD|PS-1|PS1|S182 | 14 | 73603126 | 73690399 | regulation of lysosomal vATPase assembly and function | presenilin 1 | Cell Rep. 2015 Sep 1;12(9):1430-44. Presenilin 1 Maintains Lysosomal Ca(2+) Homeostasis via TRPML1 by Regulating vATPase-Mediated Lysosome Acidification. Lee JH, McBrayer MK, Wolfe DM, Haslett LJ, Kumar A, Sato Y, Lie PP, Mohan P, Coffey EE, Kompella U, Mitchell CH, Lloyd-Evans E, Nixon RA. A role for presenilins in autophagy revisited: normal acidification of lysosomes in cells lacking PSEN1 and PSEN2. Zhang X | Lysosome |
| *RAB14* | ENSG00000119396 | FBP|RAB-14 | 9 | 123940415 | 123985292 | lysosomal membrane | RAB14, member RAS oncogene family |  | Lysosome |
| *RAB5C* | ENSG00000108774 | L1880|RAB5CL|RAB5L|RABL | 17 | 40276994 | 40307035 | lysosomal membrane | RAB5C, member RAS oncogene family |  | Lysosome |
| *RILP* | ENSG00000167705|ENSG00000274145 | PP10141 | 17 | 1549444 | 1553371 | lysosomal membrane | Rab interacting lysosomal protein |  | Lysosome |
| *RNF13* | ENSG00000082996 | EIEE73|RZF | 3 | 149530495 | 149679926 | lysosomal membrane | ring finger protein 13 |  | Lysosome |
| *SCARB2* | ENSG00000138760 | AMRF|CD36L2|EPM4|HLGP85|LGP85|LIMP-2|LIMPII|SR-BII | 4 | 77079890 | 77135046 | lysosomal membrane | LIPM2 |  | Lysosome |
| *SIDT2* | ENSG00000149577 | CGI-40 | 11 | 117049449 | 117068160 | lysosomal membrane | SID1 transmembrane family, member 2 |  | Lysosome |
| *SLC11A2* | ENSG00000110911 | AHMIO1|DCT1|DMT1|NRAMP2 | 12 | 51373184 | 51422349 | lysosomal membrane | solute carrier family 11 (proton-coupled divalent metal ion transporters), member 2 |  | Lysosome |
| *SLC15A3* | ENSG00000110446 | OCTP|PHT2|PTR3 | 11 | 60704556 | 60720002 | Transports histidine and oligopeptides from inside the lysosome to the cytosol | solute carrier family 15, member 3 | Traffic. 2007 Dec;8(12):1676-86. Integral and associated lysosomal membrane proteins. Schröder B; Clin Pharmacol Ther. 2016 Nov;100(5):431-436. Lysosomal solute carrier transporters gain momentum in research. Bissa B | Lysosome |
| *SLC17A5* | ENSG00000119899 | AST|ISSD|NSD|SD|SIALIN|SIASD|SLD | 6 | 74303102 | 74363878 | lysosomal membrane | solute carrier family 17 (anion/sugar transporter), member 5 |  | Lysosome |
| *SLC29A3* | ENSG00000198246 | ENT3|HCLAP|HJCD|PHID | 10 | 73079015 | 73123142 | Mediates nucleoside efflux across lysosome membrane | solute carrier family 29 (equilibrative nucleoside transporter), member 3 | Clin Pharmacol Ther. 2016 Nov;100(5):431-436. Lysosomal solute carrier transporters gain momentum in research. Bissa B | Lysosome |
| *SLC36A1* | ENSG00000123643 | Dct1|LYAAT1|PAT1|TRAMD3 | 5 | 150816607 | 150871942 | lysosomal membrane | solute carrier family 36 (proton/amino acid symporter), member 1 |  | Lysosome |
| *SPHK2* | ENSG00000063176 | SK 2|SK-2|SPK 2|SPK-2 | 19 | 49122548 | 49133974 | lysosomal membrane | sphingosine kinase 2 |  | Lysosome |
| *STARD3* | ENSG00000131748 | CAB1|MLN64|es64 | 17 | 37793318 | 37819737 | lysosomal membrane | StAR-related lipid transfer (START) domain containing 3 |  | Lysosome |
| *STX7* | ENSG00000079950 | - | 6 | 132767006 | 132834337 | lysosomal membrane | syntaxin 7 |  | Lysosome |
| *TCIRG1* | ENSG00000110719 | ATP6N1C|ATP6V0A3|Atp6i|OC-116kDa|OC116|OPTB1|Stv1|TIRC7|Vph1|a3 | 11 | 67806483 | 67818362 | acidification of the lysosomal compartment in osteoclasts | T-cell, immune regulator 1, ATPase, H+ transporting, lysosomal V0 subunit A3 | "Sci Rep. 2015; 5: 14827. Mapping the H+ (V)-ATPase interactome: identification of proteins involved in trafficking, folding, assembly and phosphorylationMaria Merkulova, Teodor G. Păunescu, Anie Azroyan, Vladimir Marshansky, Sylvie Breton, and Dennis Browna." | Lysosome |
| *TECPR1* | ENSG00000205356 | - | 7 | 97843936 | 97881563 | lysosomal membrane | tectonin beta-propeller repeat containing 1 |  | Lysosome |
| *TMEM192* | ENSG00000170088 | - | 4 | 165995574 | 166129701 | lysosomal membrane | transmembrane protein 192 |  | Lysosome |
| *TMEM55A* | ENSG00000155099 | PIP4P2 | 8 | 92006024 | 92053292 | lysosomal membrane | transmembrane protein 55A |  | Lysosome |
| *TMEM55B* | ENSG00000165782 | PIP4P1 | 14 | 20925878 | 20929771 | lysosomal membrane | transmembrane protein 55B |  | Lysosome |
| *TMEM59* | ENSG00000116209 | C1orf8|DCF1|HSPC001|PRO195|UNQ169 | 1 | 54497347 | 54519177 | lysosomal membrane | transmembrane protein 59 |  | Lysosome |
| *TMEM74* | ENSG00000164841 | NET36 | 8 | 109619079 | 109799844 | lysosomal membrane | transmembrane protein 74 |  | Lysosome |
| *PGAP6* | ENSG00000129925 | TMEM8A|GPI-PLA2|M83|TMEM6|TMEM8 | 16 | 420773 | 437113 | lysosomal membrane | Post-GPI Attachment To Proteins 6 |  | Lysosome |
| *TMEM9* | ENSG00000116857 | DERM4|TMEM9A | 1 | 201103900 | 201140702 | lysosomal membrane | TMEM9 |  | Lysosome |
| *TPCN1* | ENSG00000186815 | TPC1 | 12 | 113658855 | 113736390 | positive regulation of autophagy | two pore segment channel 1 | Sci Signal. 2014 May 20;7(326):ra46. Reconstituted human TPC1 is a proton-permeable ion channel and is activated by NAADP or Ca2+. Pitt SJ; Hum Mol Genet. 2012 Feb 1;21(3):511-25. Leucine-rich repeat kinase 2 regulates autophagy through a calcium-dependent pathway involving NAADP. Gómez-Suaga P | Lysosome |
| *TPCN2* | ENSG00000162341 | SHEP10|TPC2 | 11 | 68816365 | 68858072 | endolysosomal ion channel | two pore segment channel 2 | J Cell Sci. 2015 Jan 15;128(2):232-8. Dysregulation of lysosomal morphology by pathogenic LRRK2 is corrected by TPC2 inhibition. Hockey LN | Lysosome |
| *TSPAN1* | ENSG00000117472 | NET1|TM4C|TM4SF | 1 | 46640745 | 46651630 | lysosomal membrane | tetraspanin 1 |  | Lysosome |
| *VPS11* | ENSG00000160695|ENSG00000280616 | END1|HLD12|PEP5|RNF108|hVPS11 | 11 | 118938403 | 118952688 | lysosomal membrane | vacuolar protein sorting 11 homolog (S. cerevisiae) |  | Lysosome |
| *VPS16* | ENSG00000215305 | hVPS16 | 20 | 2821349 | 2847378 | lysosomal membrane | vacuolar protein sorting 16 homolog (S. cerevisiae) |  | Lysosome |
| *VPS18* | ENSG00000104142 | PEP3 | 15 | 41186628 | 41196173 | mediate vesicle trafficking steps in the endosome/lysosome pathway | vacuolar protein sorting 18 homolog (S. cerevisiae) | Traffic. 2015 Jul;16(7):727-42. Recruitment of VPS33A to HOPS by VPS16 Is Required for Lysosome Fusion with Endosomes and Autophagosomes. Wartosch L | Lysosome |
| *VPS35* | ENSG00000069329 | MEM3|PARK17 | 16 | 46690054 | 46723430 | lysosomal membrane | vacuolar protein sorting 35 homolog (S. cerevisiae) |  | Lysosome |
| *VPS39* | ENSG00000166887 | TLP|VAM6|hVam6p | 15 | 42450899 | 42500514 | lysosomal membrane | vacuolar protein sorting 39 homolog (S. cerevisiae) |  | Lysosome |
| *ZNRF2* | ENSG00000180233 | RNF202 | 7 | 30323923 | 30452118 | lysosomal membrane | zinc and ring finger 2 |  | Lysosome |
| *TRIM23* | ENSG00000113595 | ARD1|ARFD1|RNF46 | 5 | 64885507 | 64921802 | lysosomal membrane and Golgi membrane | tripartite motif containing 23 |  | Lysosome |
| *ACP2* | ENSG00000134575 | LAP | 11 | 47260853 | 47270457 | beta subunit of lysosomal acid phosphatase (LAP); lysosomal membrane protein. | acid phosphatase 2, lysosomal | 1) J Biol Chem. 1997 Jul 25;272(30):18628-35. Mice deficient in lysosomal acid phosphatase develop lysosomal storage in the kidney and central nervous system. Saftig P; 2) J Cell Sci. 2002 Jan 1;115(Pt 1):185-94. The tyrosine motifs of Lamp 1 and LAP determine their direct and indirect targetting to lysosomes. Obermüller S | Lysosome |
| *ACP5* | ENSG00000102575 | HPAP|TRACP5a|TRACP5b|TRAP|TrATPase | 19 | 11685475 | 11689823 | lysosome | acid phosphatase 5, tartrate resistant |  | Lysosome |
| *ADA* | ENSG00000196839 | ADA1 | 20 | 43248163 | 43280874 | lysosome | adenosine deaminase |  | Lysosome |
| *AGA* | ENSG00000038002 | AGU|ASRG|GA | 4 | 178351924 | 178363657 | lysosome | aspartylglucosaminidase |  | Lysosome |
| *ARSB* | ENSG00000113273 | ASB|G4S|MPS6 | 5 | 78073032 | 78281910 | sulfatase; lysosome organization; Mucopolysaccharidosis type VI is an autosomal recessive lysosomal storage disorder resulting from a deficiency of arylsulfatase B | arylsulfatase B | J Biol Chem. 1990 Feb 25;265(6):3374-81. Phylogenetic conservation of arylsulfatases. cDNA cloning and expression of human arylsulfatase B. Peters C; 2) J Biol Chem. 1991 Nov 15;266(32):21386-91. Mucopolysaccharidosis VI (Maroteaux-Lamy syndrome). An intermediate clinical phenotype caused by substitution of valine for glycine at position 137 of arylsulfatase B. Wicker G | Lysosome |
| *ARSD* | ENSG00000006756 | ASD | X | 2822011 | 2847392 | lysosome | arylsulfatase D |  | Lysosome |
| *ARSG* | ENSG00000141337 | USH4 | 17 | 66255323 | 66418872 | lysosome | arylsulfatase G |  | Lysosome |
| *BCL10* | ENSG00000142867 | CARMEN|CIPER|CLAP|IMD37|c-E10|mE10 | 1 | 85731931 | 85742773 | lysosome | B-cell CLL/lymphoma 10 |  | Lysosome |
| *CHID1* | ENSG00000177830 | GL008|SI-CLP|SICLP | 11 | 867357 | 915058 | lysosome | chitinase domain containing 1 |  | Lysosome |
| *CHIT1* | ENSG00000133063 | CHI3|CHIT|CHITD | 1 | 203181955 | 203242769 | lysosome | chitinase 1 (chitotriosidase) |  | Lysosome |
| *CST3* | ENSG00000101439 | ARMD11|HEL-S-2 | 20 | 23608534 | 23619110 | is a powerful endogenous inhibitor of the lysosomal cysteine proteases CB,CL and cathepsin H (CH) | cystatin C | Neurotoxicology. 2006 Mar;27(2):260-76. Enhanced cystatin C and lysosomal protease expression following 6-hydroxydopamine exposure. Lee DC | Lysosome |
| *CTBS* | ENSG00000117151 | CTB | 1 | 85015289 | 85040163 | lysosome | chitobiase, di-N-acetyl- | Proteomics. 2005 Apr;5(6):1520-32. The human brain mannose 6-phosphate glycoproteome: a complex mixture composed of multiple isoforms of many soluble lysosomal proteins. Sleat DE | Lysosome |
| *CTSA* | ENSG00000064601 | GLB2|GSL|NGBE|PPCA|PPGB | 20 | 44518783 | 44527459 | Lysosomal serine protease | cathepsin A | Hiraiwa M. Cathepsin A/protective protein: An unusual lysosomal multifunctional protein. Cell Mol Life Sci. 1999;56(11-12):894–907. | Lysosome |
| *CTSC* | ENSG00000109861 | CPPI|DPP-I|DPP1|DPPI|HMS|JP|JPD|PALS|PDON1|PLS | 11 | 88026760 | 88070955 | Lysosomal cysteine proteinases hat appears to be a central coordinator for activation of many serine proteinases in cells of the immune system. | cathepsin C | J Biol Chem. 1995 Sep 15;270(37):21626-31. Oligomeric structure and substrate induced inhibition of human cathepsin C. Dolenc I | Lysosome |
| *CTSD* | ENSG00000117984 | CLN10|CPSD|HEL-S-130P | 11 | 1773982 | 1785222 | Lysosomal aspartic protease | cathepsin D | Brix, K. (2005) Lysosomal proteases. Revival of the Sleeping Beauty. in Lysosomes (Saftig, P., ed) pp. 50 –59, Springer, New York | Lysosome |
| *CTSF* | ENSG00000174080 | CATSF|CLN13 | 11 | 66330934 | 66336312 | Lysosomal endopeptidase | cathepsin F | J Biol Chem. 1998 Nov 27;273(48):32000-8. Human cathepsin F. Molecular cloning, functional expression, tissue localization, and enzymatic characterization. Wang B | Lysosome |
| *CTSH* | ENSG00000103811 | ACC-4|ACC-5|ACC4|ACC5|CPSB | 15 | 79213400 | 79241916 | Lysosomal cysteine proteinases important in the overall degradation of lysosomal proteins | cathepsin H | Brix, K. (2005) Lysosomal proteases. Revival of the Sleeping Beauty. in Lysosomes (Saftig, P., ed) pp. 50 –59, Springer, New York | Lysosome |
| *CTSO* | ENSG00000256043|ENSG00000263238 | CTSO1 | 4 | 156845270 | 156875069 | Proteolysis | cathepsin O | Proteomics. 2010 Nov;10(22):4053-76. The proteome of lysosomes. Schröder BA | Lysosome |
| *CTSZ* | ENSG00000101160 | CTSX | 20 | 57570240 | 57582302 | lysosomal cysteine proteases that digest polyQ proteins and peptides | cathepsin Z | J Biol Chem. 2012 May 18;287(21):17471-82. Cathepsins L and Z are critical in degrading polyglutamine-containing proteins within lysosomes. Bhutani N | Lysosome |
| *DNASE2* | ENSG00000105612 | DNASE2A|DNL|DNL2 | 19 | 12986025 | 12992282 | lysosome | deoxyribonuclease II, lysosomal |  | Lysosome |
| *DOC2A* | ENSG00000149927 | Doc2 | 16 | 30016830 | 30034591 | lysosome | double C2-like domains, alpha |  | Lysosome |
| *DRAM2* | ENSG00000156171 | CORD21|PRO180|TMEM77|WWFQ154 | 1 | 111659955 | 111682838 | lysosome | DNA-damage regulated autophagy modulator 2 |  | Lysosome |
| *EPDR1* | ENSG00000086289 | EPDR|MERP-1|MERP1|UCC1 | 7 | 37723446 | 37991543 | lysosome | ependymin related protein 1 (zebrafish) |  | Lysosome |
| *FNBP1* | ENSG00000187239 | FBP17 | 9 | 132649466 | 132805473 | lysosome | formin binding protein 1 |  | Lysosome |
| *FUCA1* | ENSG00000179163 | FUCA | 1 | 24171567 | 24194784 | lysosome | fucosidase, alpha-L- 1, tissue |  | Lysosome |
| *GALC* | ENSG00000054983 | - | 14 | 88304164 | 88460009 | lysosome | galactosylceramidase |  | Lysosome |
| *GGH* | ENSG00000137563 | GH | 8 | 63927638 | 63951730 | lysosome | gamma-glutamyl hydrolase (conjugase, folylpolygammaglutamyl hydrolase) | Proteomics. 2005 Apr;5(6):1520-32. The human brain mannose 6-phosphate glycoproteome: a complex mixture composed of multiple isoforms of many soluble lysosomal proteins. Sleat DE | Lysosome |
| *GLA* | ENSG00000102393 | GALA | X | 100652791 | 100662913 | lysosome | galactosidase, alpha |  | Lysosome |
| *HCK* | ENSG00000101336 | JTK9|p59Hck|p61Hck | 20 | 30639991 | 30689659 | lysosome | hemopoietic cell kinase |  | Lysosome |
| *HPS1* | ENSG00000107521 | BLOC3S1|HPS | 10 | 100175955 | 100206684 | components of a protein complex that regulates the intracellular localization of lysosomes and late endosomes | Hermansky-Pudlak syndrome 1 | Proc Natl Acad Sci U S A. 2003 Jul 22;100(15):8770-5. Biogenesis of lysosome-related organelles complex 3 (BLOC-3): a complex containing the Hermansky-Pudlak syndrome (HPS) proteins HPS1 and HPS4. Nazarian R | Lysosome |
| *HPS4* | ENSG00000100099 | BLOC3S2|LE | 22 | 26839389 | 26879803 | biogenesis of lysosome-related organelles | Hermansky-Pudlak syndrome 4 | J Biol Chem. 2010 Mar 5;285(10):7794-804. Assembly of the biogenesis of lysosome-related organelles complex-3 (BLOC-3) and its interaction with Rab9. Kloer DP, Rojas R, Ivan V, Moriyama K, van Vlijmen T, Murthy N, Ghirlando R, van der Sluijs P, Hurley JH, Bonifacino JS. | Lysosome |
| *HPSE* | ENSG00000173083 | HPA|HPA1|HPR1|HPSE1|HSE1 | 4 | 84213614 | 84256306 | lysosome | heparanase |  | Lysosome |
| *HYAL1* | ENSG00000114378 | HYAL-1|LUCA1|MPS9|NAT6 | 3 | 50337320 | 50349812 | lysosome | hyaluronoglucosaminidase 1 |  | Lysosome |
| *HYAL2* | ENSG00000068001 | LUCA2 | 3 | 50355221 | 50360337 | lysosome | hyaluronoglucosaminidase 2 |  | Lysosome |
| *HYAL3* | ENSG00000186792 | HYAL-3|LUCA-3|LUCA3 | 3 | 50330262 | 50336899 | lysosome | hyaluronoglucosaminidase 3 |  | Lysosome |
| *IDUA* | ENSG00000127415 | IDA|MPS1|MPSI | 4 | 980785 | 998316 | lysosomal degradation of these glycosaminoglycans; Mutations in this gene that result in enzymatic deficiency lead to the autosomal recessive disease mucopolysaccharidosis type I (MPS I). | iduronidase, alpha-L- | Clin Genet. 2015 Oct;88(4):376-80. p.L18P: a novel IDUA mutation that causes a distinct attenuated phenotype in mucopolysaccharidosis type I patients. Pasqualim G | Lysosome |
| *IFI30* | ENSG00000216490 | GILT|IFI-30|IP-30|IP30 | 19 | 18283972 | 18288927 | lysosome | interferon, gamma-inducible protein 30 |  | Lysosome |
| *ITM2C* | ENSG00000135916 | BRI3|BRICD2C|E25|E25C|ITM3 | 2 | 231729354 | 231743963 | lysosome | integral membrane protein 2C |  | Lysosome |
| *KCNE1* | ENSG00000180509 | ISK|JLNS|JLNS2|LQT2/5|LQT5|MinK | 21 | 35818988 | 35884573 | lysosome | potassium voltage-gated channel, Isk-related family, member 1 |  | Lysosome |
| *LAMP1* | ENSG00000185896 | CD107a|LAMPA|LGP120 | 13 | 113951556 | 113977987 | Structural protein | LAMP1 | Nat Rev Mol Cell Biol. 2013 May;14(5):283-96. Signals from the lysosome: a control centre for cellular clearance and energy metabolism. Settembre C, Fraldi A, Medina DL, Ballabio A. | Lysosome |
| *LIPA* | ENSG00000107798 | CESD|LAL | 10 | 90973326 | 91174314 | This enzyme functions in the lysosome to catalyze the hydrolysis of cholesteryl esters and triglycerides. Mutations in this gene can result in Wolman disease | lipase A, lysosomal acid, cholesterol esterase | BMJ Case Rep. 2014 May 15;2014. Infant case of lysosomal acid lipase deficiency: Wolman's disease. Sadhukhan M | Lysosome |
| *LRBA* | ENSG00000198589 | BGL|CDC4L|CVID8|LAB300|LBA | 4 | 151185594 | 151936879 | lysosome | LPS-responsive vesicle trafficking, beach and anchor containing |  | Lysosome |
| *LRP2* | ENSG00000081479 | DBS|GP330|LRP-2 | 2 | 169983619 | 170219195 | lysosome | low density lipoprotein receptor-related protein 2 |  | Lysosome |
| *MAN2B1* | ENSG00000104774 | LAMAN|MANB | 19 | 12757325 | 12777556 | lysosome | mannosidase, alpha, class 2B, member 1 |  | Lysosome |
| *MAN2B2* | ENSG00000013288 | - | 4 | 6576902 | 6625089 | lysosome | mannosidase, alpha, class 2B, member 2 |  | Lysosome |
| *MANBA* | ENSG00000109323 | MANB1 | 4 | 103552660 | 103682151 | lysosome | mannosidase, beta A, lysosomal |  | Lysosome |
| *MARCH8* | ENSG00000165406 | CMIR|MARCH-VIII|MARCHF8|MIR|RNF178|c-MIR | 10 | 45950035 | 46090354 | lysosome | membrane-associated ring finger (C3HC4) 8, E3 ubiquitin protein ligase |  | Lysosome |
| *NAGA* | ENSG00000198951 | D22S674|GALB | 22 | 42454358 | 42466846 | lysosome | N-acetylgalactosaminidase, alpha- |  | Lysosome |
| *NAGLU* | ENSG00000108784 | CMT2V|MPS-IIIB|MPS3B|NAG|UFHSD | 17 | 40688190 | 40696467 | enzyme that degrades heparan sulfate by hydrolysis of terminal N-acetyl-D-glucosamine residues in N-acetyl-alpha-D-glucosaminides. Defects in this gene are the cause of mucopolysaccharidosis type IIIB (MPS-IIIB), also known as Sanfilippo syndrome B. | N-acetylglucosaminidase, alpha | Proc Natl Acad Sci U S A. 1996 Jun 11;93(12):6101-5. The molecular basis of Sanfilippo syndrome type B. Zhao HG | Lysosome |
| *NEU1* | ENSG00000184494|ENSG00000204386|ENSG00000223957|ENSG00000227129|ENSG00000227315|ENSG00000228691|ENSG00000234343|ENSG00000234846 | NANH|NEU|SIAL1 | 6 | 31825436 | 31830683 | lysosome | sialidase 1 (lysosomal sialidase) |  | Lysosome |
| *NEU4* | ENSG00000204099|ENSG00000277926 | - | 2 | 242749920 | 242758739 | lysosome | sialidase 4 |  | Lysosome |
| *NPC2* | ENSG00000119655 | EDDM1|HE1 | 14 | 74942895 | 74960880 | lysosome | NPC2 |  | Lysosome |
| *PCSK9* | ENSG00000169174 | FH3|FHCL3|HCHOLA3|LDLCQ1|NARC-1|NARC1|PC9 | 1 | 55505221 | 55530525 | lysosome | proprotein convertase subtilisin/kexin type 9 |  | Lysosome |
| *PCYOX1* | ENSG00000116005 | PCL1 | 2 | 70484518 | 70508323 | lysosome | prenylcysteine oxidase 1 |  | Lysosome |
| *PEBP4* | ENSG00000134020 | CORK-1|CORK1|GWTM1933|HEL-S-300|PEBP-4|PRO4408|hPEBP4 | 8 | 22570769 | 22857513 | lysosome | phosphatidylethanolamine-binding protein 4 |  | Lysosome |
| *PPT2* | ENSG00000168452|ENSG00000206256|ENSG00000206329|ENSG00000221988|ENSG00000227600|ENSG00000228116|ENSG00000231618|ENSG00000236649 | C6orf8|G14|PPT-2 | 6 | 32121218 | 32134011 | lysosomal hydrolase | palmitoyl-protein thioesterase 2 | J Biol Chem. 2003 Sep 26;278(39):37957-64. The crystal structure of palmitoyl protein thioesterase-2 (PPT2) reveals the basis for divergent substrate specificities of the two lysosomal thioesterases, PPT1 and PPT2. Calero G | Lysosome |
| *PRCP* | ENSG00000137509 | HUMPCP|PCP | 11 | 82534544 | 82681626 | enzyme highly concentrated in lysosomes; may also be active extracellularly after their release from lysosomes in soluble form or in a plasma membrane-bound complex | prolylcarboxypeptidase (angiotensinase C) | Immunol Rev. 1998 Feb;161:129-41. Cellular carboxypeptidases. Skidgel RA | Lysosome |
| *PRDX6* | ENSG00000117592 | 1-Cys|AOP2|HEL-S-128m|NSGPx|PRX|aiPLA2|p29 | 1 | 173446405 | 173457946 | lysosome | peroxiredoxin 6 |  | Lysosome |
| *RAB27A* | ENSG00000069974 | GS2|HsT18676|RAB27|RAM | 15 | 55495164 | 55611311 | lysosome | RAB27A, member RAS oncogene family |  | Lysosome |
| *RAMP2* | ENSG00000131477 | - | 17 | 40910465 | 40915059 | lysosomal transport | RAMP2 | Nat Rev Mol Cell Biol. 2013 May;14(5):283-96. Signals from the lysosome: a control centre for cellular clearance and energy metabolism. Settembre C, Fraldi A, Medina DL, Ballabio A. | Lysosome |
| *RAMP3* | ENSG00000122679 | - | 7 | 45197390 | 45225901 | lysosome | receptor (G protein-coupled) activity modifying protein 3 |  | Lysosome |
| *RNASET2* | ENSG00000026297 | RNASE6PL|bA514O12.3 | 6 | 167342992 | 167370679 | lysosome | ribonuclease T2 |  | Lysosome |
| *SIAE* | ENSG00000110013 | AIS6|CSE-C|CSEC|LSE|YSG2 | 11 | 124503009 | 124565603 | lysosome | sialic acid acetylesterase |  | Lysosome |
| *SPACA3* | ENSG00000141316 | ALLP17|CT54|LYC3|LYZC|LYZL3|SLLP1 | 17 | 31297394 | 31324895 | lysosome | sperm acrosome associated 3 |  | Lysosome |
| *SRC* | ENSG00000197122 | ASV|SRC1|THC6|c-SRC|p60-Src | 20 | 35973088 | 36034453 | lysosome | v-src avian sarcoma (Schmidt-Ruppin A-2) viral oncogene homolog |  | Lysosome |
| *STS* | ENSG00000101846 | ARSC|ARSC1|ASC|ES|SSDD|XLI | X | 7137497 | 7272851 | lysosome | steroid sulfatase (microsomal), isozyme S |  | Lysosome |
| *TPP1* | ENSG00000166340 | CLN2|GIG1|LPIC|SCAR7|TPP-1 | 11 | 6634000 | 6640692 | lysosomal aminopeptidase | tripeptidyl peptidase I | Hum Mutat. 2010 Jun;31(6):710-21. Functional consequences and rescue potential of pathogenic missense mutations in tripeptidyl peptidase I. Walus M | Lysosome |
| *UNC93B1* | ENSG00000110057 | IIAE1|UNC93|UNC93B|Unc-93B1 | 11 | 67758575 | 67772452 | lysosome | unc-93 homolog B1 (C. elegans) |  | Lysosome |
| *VMA21* | ENSG00000160131 | MEAX|XMEA | X | 150564987 | 150577836 | lysosomal V-ATPase assembly chaperone | VMA21 vacuolar H+-ATPase homolog (S. cerevisiae) | Acta Neuropathol. 2013 Mar;125(3):439-57. VMA21 deficiency prevents vacuolar ATPase assembly and causes autophagic vacuolar myopathy. Ramachandran N, Munteanu I, Wang P, Ruggieri A, Rilstone JJ, Israelian N, Naranian T, Paroutis P, Guo R, Ren ZP, Nishino I, Chabrol B, Pellissier JF, Minetti C, Udd B, Fardeau M, Tailor CS, Mahuran DJ, Kissel JT, Kalimo H, Levy N, Manolson MF, Ackerley CA, Minassian BA. | Lysosome |
| *WDR48* | ENSG00000114742 | P80|SPG60|UAF1 | 3 | 39093489 | 39138155 | lysosome | WD repeat domain 48 |  | Lysosome |
| *ZNRF1* | ENSG00000186187 | NIN283 | 16 | 75032928 | 75144892 | lysosome | zinc and ring finger 1, E3 ubiquitin protein ligase |  | Lysosome |
| *ATP6AP1* | ENSG00000071553 | 16A|ATP6IP1|ATP6S1|Ac45|CF2|VATPS1|XAP-3|XAP3 | X | 153656978 | 153664862 |  | ATPase, H+ transporting, lysosomal accessory protein 1 | Curr Protein Pept Sci. 2012 Mar;13(2):124-33. Novel insights into V-ATPase functioning: distinct roles for its accessory subunits ATP6AP1/Ac45 and ATP6AP2/(pro) renin receptor. Jansen EJ, Martens GJ. | Lysosome-related genes |
| *ATP6AP2* | ENSG00000182220 | APT6M8-9|ATP6IP2|ATP6M8-9|CDG2R|ELDF10|HT028|M8-9|MRXE|MRXSH|MSTP009|PRR|RENR|XMRE|XPDS | X | 40440146 | 40465889 | associated with the transmembrane sector of the V-type ATPases | ATPase, H+ transporting, lysosomal accessory protein 2 | EMBO J. 2011 Jul 29;30(16):3242-58. Regulation of TFEB and V-ATPases by mTORC1. Peña-Llopis S | Lysosome-related genes |
| *ATP6V0A1* | ENSG00000033627 | ATP6N1|ATP6N1A|Stv1|VPP1|Vph1|a1 | 17 | 40610862 | 40674629 | mediating autophagosome-lysosome fusion and lysosomal acidification | ATPase, H+ transporting, lysosomal V0 subunit a1 | Eur J Neurosci. 2013 Jun; 37(12): 1949–1961. Autophagy failure in Alzheimer’s disease and the role of defective lysosomal acidificationDevin M. Wolfe, Ju-hyun Lee, Asok Kumar, Sooyeon Lee, Samantha J. Orenstein, and Ralph A. Nixon | Lysosome-related genes |
| *ATP6V0B* | ENSG00000117410 | ATP6F|HATPL|VMA16 | 1 | 44440159 | 44443967 | lysosomal acidification (Palmieri_Table 1) | ATPase, H+ transporting, lysosomal 21kDa, V0 subunit b | Hum Mol Genet. 2011 Oct 1;20(19):3852-66. Characterization of the CLEAR network reveals an integrated control of cellular clearance pathways. Palmieri M | Lysosome-related genes |
| *ATP6V0E1* | ENSG00000113732 | ATP6H|ATP6V0E|M9.2|Vma21|Vma21p | 5 | 172410760 | 172462448 | ATP hydrolysis coupled proton transport; lysosomal acidification (Palmieri_Table 1) | ATPase, H+ transporting, lysosomal 9kDa, V0 subunit e1 | Hum Mol Genet. 2011 Oct 1;20(19):3852-66. Characterization of the CLEAR network reveals an integrated control of cellular clearance pathways. Palmieri M | Lysosome-related genes |
| *ATP6V0E2* | ENSG00000171130 | ATP6V0E2L|C7orf32 | 7 | 149570057 | 149577784 | regulation of macroautophagy | ATPase, H+ transporting V0 subunit e2 | Gene. 2007 May 15;393(1-2):94-100. Molecular cloning and characterization of a novel form of the human vacuolar H+-ATPase e-subunit: an essential proton pump component. Blake-Palmer KG, Su Y, Smith AN, Karet FE. | Lysosome-related genes |
| *ATP6V1A* | ENSG00000114573 | ARCL2D|ATP6A1|ATP6V1A1|HO68|IECEE3|VA68|VPP2|Vma1 | 3 | 113465866 | 113530903 | lysosomal acidification (Palmieri_Table 1) | ATPase, H+ transporting, lysosomal 70kDa, V1 subunit A | 1) Hum Mol Genet. 2011 Oct 1;20(19):3852-66. Characterization of the CLEAR network reveals an integrated control of cellular clearance pathways. Palmieri M; 2) Traffic. 2007 Dec;8(12):1676-86. Integral and associated lysosomal membrane proteins. Schröder B. | Lysosome-related genes |
| *ATP6V1B1* | ENSG00000116039 | ATP6B1|RTA1B|VATB|VMA2|VPP3 | 2 | 71163012 | 71192536 | lysosomal acidification | ATPase, H+ transporting, lysosomal 56/58kDa, V1 subunit B1 | 1) Hum Mol Genet. 2011 Oct 1;20(19):3852-66. Characterization of the CLEAR network reveals an integrated control of cellular clearance pathways. Palmieri M; 2) Traffic. 2007 Dec;8(12):1676-86. Integral and associated lysosomal membrane proteins. Schröder B. | Lysosome-related genes |
| *ATP6V1B2* | ENSG00000147416 | ATP6B1B2|ATP6B2|DOOD|HO57|VATB|VPP3|Vma2|ZLS2 | 8 | 20054878 | 20084330 | lysosomal acidification | ATPase, H+ transporting, lysosomal 56/58kDa, V1 subunit B2 | 1) Traffic. 2007 Dec;8(12):1676-86. Integral and associated lysosomal membrane proteins. Schröder B; 2) Cell Res. 2014 Nov;24(11):1370-3. De novo mutation in ATP6V1B2 impairs lysosome acidification and causes dominant deafness-onychodystrophy syndrome. Yuan Y | Lysosome-related genes |
| *ATP6V1C1* | ENSG00000155097 | ATP6C|ATP6D|VATC|Vma5 | 8 | 104033291 | 104085279 | lysosomal acidification (Palmieri_Table 1) | ATPase, H+ transporting, lysosomal 42kDa, V1 subunit C1 | 1) Hum Mol Genet. 2011 Oct 1;20(19):3852-66. Characterization of the CLEAR network reveals an integrated control of cellular clearance pathways. Palmieri M; 2) Traffic. 2007 Dec;8(12):1676-86. Integral and associated lysosomal membrane proteins. Schröder B. | Lysosome-related genes |
| *ATP6V1C2* | ENSG00000143882 | ATP6C2|VMA5 | 2 | 10861775 | 10925236 | acidification of eukaryotic intracellular organelles | ATPase, H+ transporting, lysosomal 42kDa, V1 subunit C2 | 1) Hum Mol Genet. 2011 Oct 1;20(19):3852-66. Characterization of the CLEAR network reveals an integrated control of cellular clearance pathways. Palmieri M; 2) Traffic. 2007 Dec;8(12):1676-86. Integral and associated lysosomal membrane proteins. Schröder B. | Lysosome-related genes |
| *ATP6V1D* | ENSG00000100554 | ATP6M|VATD|VMA8 | 14 | 67761088 | 67826982 | ATP hydrolysis coupled proton transport; lysosomal acidification (Palmieri_Table 1) | ATPase, H+ transporting, lysosomal 34kDa, V1 subunit D | Traffic. 2007 Dec;8(12):1676-86. Integral and associated lysosomal membrane proteins. Schröder B | Lysosome-related genes |
| *ATP6V1E1* | ENSG00000131100 | ARCL2C|ATP6E|ATP6E2|ATP6V1E|P31|Vma4 | 22 | 18074902 | 18111584 | lysosomal acidification (Palmieri_Table 1) | ATPase, H+ transporting, lysosomal 31kDa, V1 subunit E1 | 1) Hum Mol Genet. 2011 Oct 1;20(19):3852-66. Characterization of the CLEAR network reveals an integrated control of cellular clearance pathways. Palmieri M; 2) Traffic. 2007 Dec;8(12):1676-86. Integral and associated lysosomal membrane proteins. Schröder B. | Lysosome-related genes |
| *ATP6V1E2* | ENSG00000250565 | ATP6E1|ATP6EL2|ATP6V1EL2|VMA4 | 2 | 46717889 | 46769696 | acidification of eukaryotic intracellular organelles; regulation of macroautophagy (Hohn) | ATPase, H+ transporting, lysosomal 31kDa, V1 subunit E2 | Gene. 2002 May 1;289(1-2):7-12. A human gene, ATP6E1, encoding a testis-specific isoform of H(+)-ATPase subunit E. Imai-Senga Y, Sun-Wada GH, Wada Y, Futai M. | Lysosome-related genes |
| *ATP6V1F* | ENSG00000128524 | ATP6S14|VATF|Vma7 | 7 | 128502880 | 128505898 | acidification of eukaryotic intracellular organelles | ATPase, H+ transporting, lysosomal 14kDa, V1 subunit F | Theranostics. 2018; 8(19): 5379–5399. V-ATPases and osteoclasts: ambiguous future of V-ATPases inhibitors in osteoporosisXiaohong Duan, Shaoqing Yang, Lei Zhang, and Tielin Yang | Lysosome-related genes |
| *ATP6V1G1* | ENSG00000136888 | ATP6G|ATP6G1|ATP6GL|ATP6J|Vma10 | 9 | 117350026 | 117360653 | lysosomal acidification (Palmieri_Table 1) | ATPase, H+ transporting, lysosomal 13kDa, V1 subunit G1 | 1) Hum Mol Genet. 2011 Oct 1;20(19):3852-66. Characterization of the CLEAR network reveals an integrated control of cellular clearance pathways. Palmieri M; 2) Traffic. 2007 Dec;8(12):1676-86. Integral and associated lysosomal membrane proteins. Schröder B. | Lysosome-related genes |
| *ATP6V1G2* | ENSG00000206445|ENSG00000213760|ENSG00000226850|ENSG00000227587|ENSG00000230900|ENSG00000234668|ENSG00000234920 | ATP6G|ATP6G2|NG38|VMA10 | 6 | 31512239 | 31516204 | acidification of eukaryotic intracellular organelles; regulation of macroautophagy (Hohn) | ATPase, H+ transporting, lysosomal 13kDa, V1 subunit G2 | Theranostics. 2018; 8(19): 5379–5399. V-ATPases and osteoclasts: ambiguous future of V-ATPases inhibitors in osteoporosisDuan X, Yang S, Zhang L, Yang T. | Lysosome-related genes |
| *ATP6V1G3* | ENSG00000151418|ENSG00000263014 | ATP6G3|Vma10 | 1 | 198492352 | 198510075 | acidification of eukaryotic intracellular organelles | ATPase, H+ transporting, lysosomal 13kDa, V1 subunit G3 | Sci Rep. 2015; 5: 14827. Mapping the H+ (V)-ATPase interactome: identification of proteins involved in trafficking, folding, assembly and phosphorylationMaria Merkulova, Teodor G. Păunescu, Anie Azroyan, Vladimir Marshansky, Sylvie Breton, and Dennis Browna. | Lysosome-related genes |
| *ATP6V1H* | ENSG00000047249 | CGI-11|MSTP042|NBP1|SFD|SFDalpha|SFDbeta|VMA13 | 8 | 54628117 | 54756118 | lysosomal acidification (Palmieri_Table 1) | ATPase H+ Transporting V1 Subunit H | 1) Hum Mol Genet. 2011 Oct 1;20(19):3852-66. Characterization of the CLEAR network reveals an integrated control of cellular clearance pathways. Palmieri M; 2) Traffic. 2007 Dec;8(12):1676-86. Integral and associated lysosomal membrane proteins. Schröder B. | Lysosome-related genes |
| *CLCN3* | ENSG00000109572 | CLC3|ClC-3 | 4 | 170533784 | 170644824 | ClC-3 is ubiquitously expressed and is present in brain synaptic vesicles as well as in late endosome-lysosome compartments in non-neuronal cell lines | chloride channel, voltage-sensitive 3 | J Biol Chem. 2004 Jun 11;279(24):25430-9. AP-3-dependent mechanisms control the targeting of a chloride channel (ClC-3) in neuronal and non-neuronal cells. Salazar G | Lysosome-related genes |
| *CLN6* | ENSG00000128973 | CLN4A|HsT18960|nclf | 15 | 68499330 | 68549549 | involved in the degradation of post-translationally modified proteins in lysosomes | ceroid-lipofuscinosis, neuronal 6, late infantile, variant | Eur J Biochem. 2001 Nov;268(22):5851-6. Elevated lysosomal pH in neuronal ceroid lipofuscinoses (NCLs). Holopainen JM | Lysosome-related genes |
| *CPQ* | ENSG00000104324 | LDP|PGCP | 8 | 97657455 | 98161882 | plasma glutamate carboxypeptidase,lysosomal dipeptidase | carboxypeptidase Q | Proteomics. 2005 Apr;5(6):1520-32. The human brain mannose 6-phosphate glycoproteome: a complex mixture composed of multiple isoforms of many soluble lysosomal proteins. Sleat DE | Lysosome-related genes |
| *CSTB* | ENSG00000160213 | CPI-B|CST6|EPM1|EPM1A|PME|STFB|ULD | 21 | 45192393 | 45196326 | lysosomal cysteine protease inhibitor | cystatin B | Eur J Hum Genet. 2005 Feb;13(2):208-15. Loss of lysosomal association of cystatin B proteins representing progressive myoclonus epilepsy, EPM1, mutations. Alakurtti K | Lysosome-related genes |
| *CTSE* | ENSG00000196188 | CATE | 1 | 206317459 | 206332104 | endosomal aspartic proteinase that is predominantly expressed in immune-related cells; deficiency causes autophagy impairment concomitantly with increased aberrant mitochondria as well as increased oxidative stress. | cathepsin E | PLoS One. 2013 Dec 5;8(12)Cathepsin E deficiency impairs autophagic proteolysis in macrophages. Tsukuba T | Lysosome-related genes |
| *CTSG* | ENSG00000100448 | CATG|CG | 14 | 25042728 | 25045466 | Lysosomal serine protease | cathepsin G | MacIvor DM, Shapiro SD, Pham CT. et al. Normal neutrophil function in cathepsin G-deficient mice. Blood. 1999;94(12):4282–4293. | Lysosome-related genes |
| *CTSL* | ENSG00000135047 | CATL|CTSL1|MEP | 9 | 90340434 | 90346308 | lysosomal cysteine proteases that digest polyQ proteins and peptides | cathepsin L | J Biol Chem. 2012 May 18;287(21):17471-82. Cathepsins L and Z are critical in degrading polyglutamine-containing proteins within lysosomes. Bhutani N | Lysosome-related genes |
| *Hps5* | ENSG00000110756|ENSG00000288445 | AIBP63|BLOC2S2 | 11 | 18300223 | 18343745 | Lysosome Biogenesis | Hermansky-Pudlak syndrome 5 |  | Lysosome-related genes |
| *MT3* | ENSG00000087250 | GIF|GIFB|GRIF|ZnMT3 | 16 | 56622986 | 56625000 | Lysosome localization | metallothionein 3 | Mol Brain. 2010 Oct 26;3(1):30. Roles of zinc and metallothionein-3 in oxidative stress-induced lysosomal dysfunction, cell death, and autophagy in neurons and astrocytes. Lee SJ, Koh JY. | Lysosome-related genes |
| *PSEN2* | ENSG00000143801 | AD3L|AD4|CMD1V|PS2|STM2 | 1 | 227057885 | 227083806 | building up of an intracellular Aβ Pool | presenilin 2 (Alzheimer disease 4) | Cell. 2016 Jun 30;166(1):193-208. Restricted Location of PSEN2/γ-Secretase Determines Substrate Specificity and Generates an Intracellular Aβ Pool. Sannerud R, Esselens C, Ejsmont P, Mattera R, Rochin L, Tharkeshwar AK, De Baets G, De Wever V, Habets R, Baert V, Vermeire W, Michiels C, Groot AJ, Wouters R, Dillen K, Vints K, Baatsen P, Munck S, Derua R, Waelkens E, Basi GS, Mercken M, Vooijs M, Bollen M, Schymkowitz J, Rousseau F, Bonifacino JS, Van Niel G, De Strooper B, Annaert W. | Lysosome-related genes |
| *RAB34* | ENSG00000109113 | NARR|RAB39|RAH | 17 | 27041299 | 27045447 | Lysosome localization | RAB34, member RAS oncogene family |  | Lysosome-related genes |
| *RILPL1* | ENSG00000188026 | GOSPEL|RLP1 | 12 | 123955925 | 124018265 | regulation lysosome morphology | Rab interacting lysosomal protein-like 1 | J Cell Biol. 2004 Mar 29;164(7):1065-76. Localization of the AP-3 adaptor complex defines a novel endosomal exit site for lysosomal membrane proteins. Peden AA, Oorschot V, Hesser BA, Austin CD, Scheller RH, Klumperman J. | Lysosome-related genes |
| *SCPEP1* | ENSG00000121064 | HSCP1|RISC | 17 | 55055466 | 55084129 | Lysosomal hydrolases and accessory proteins | retinoid-inducible serine carboxypeptidase 1 | 1) FEBS J. 2009 Mar;276(5):1356-69. Molecular characterization and gene disruption of mouse lysosomal putative serine carboxypeptidase 1. Kollmann K; 2) Hum Mol Genet. 2011 Oct 1;20(19):3852-66. Characterization of the CLEAR network reveals an integrated control of cellular clearance pathways. Palmieri M | Lysosome-related genes |
| *SLC38A9* | ENSG00000177058 | URLC11 | 5 | 54921673 | 55069022 | autophagic amino acid sensing (by direct interaction with mTOR complex) | solute carrier family 38, member 9 | Science. 2017 Mar 24;355(6331):1306-1311. Lysosomal cholesterol activates mTORC1 via an SLC38A9-Niemann-Pick C1 signaling complex. Castellano BM, Thelen AM, Moldavski O, Feltes M, van der Welle RE, Mydock-McGrane L, Jiang X, van Eijkeren RJ, Davis OB, Louie SM, Perera RM, Covey DF, Nomura DK, Ory DS, Zoncu R. | Lysosome-related genes |
| *LAMTOR1* | ENSG00000149357 | C11orf59|PDRO|Ragulator1|p18|p27RF-Rho | 11 | 71796941 | 71814433 | mTOR regulators | late endosomal/lysosomal adaptor, MAPK and MTOR activator 1 | Methods Enzymol. 2014;535:249-63. p18/LAMTOR1: a late endosome/lysosome-specific anchor protein for the mTORC1/MAPK signaling pathway. Nada S, Mori S, Takahashi Y, Okada M. | Lysosome-related genes |

**Supplementary Table 2.** SMR and colocalization results of the association between exposures of autophagy-related genes and ALS risk.

| **QTL** | **Tissue** | **Gene** | **Probe** | **Probe Chr.** | **Probe**  **base pair** | **Top**  **SNP** | **SNP**  **Chr.** | **SNP**  **base pair** | **Effect allele** | **Other allele** | **Effect allele frequency** | **GWAS association** | | | **QTL association** | | | **SMR association** | | | | **HEIDI Test** | | **PP.H4** | **F-statistic** |
| --- | --- | --- | --- | --- | --- | --- | --- | --- | --- | --- | --- | --- | --- | --- | --- | --- | --- | --- | --- | --- | --- | --- | --- | --- | --- |
|  |  |  |  |  |  |  |  |  |  |  |  | **β** | **SE** | ***P*** | **β** | **SE** | ***P*** | **β** | **SE** | ***P*** | **FDR *P*** | ***P*** | **No. of SNPs** |  |  |
| mQTL | blood | *C9orf72* | cg05990720 | 9 | 27573650 | rs9969832 | 9 | 27493063 | T | C | 0.230616 | 0.1719 | 0.0129 | 1.71E-40 | -0.289872 | 0.0363267 | 1.47E-15 | -0.593021 | 0.086623 | 7.59E-12 | 1.44E-08 | 0.146 | 20 | 0.92 | 63.67 |
|  | brain | *IDUA* | cg08160350 | 4 | 996052 | rs6599388 | 4 | 939087 | C | T | 0.699801 | -0.057 | 0.0127 | 7.63E-06 | 1.33247 | 0.0359488 | 1.00E-300 | -0.0427777 | 0.0096008 | 8.36E-06 | 5.38E-03 | 0.021 | 20 | 0.97 | 1373.87 |
|  |  | *IDUA* | cg01572696 | 4 | 995849 | rs6599388 | 4 | 939087 | C | T | 0.699801 | -0.057 | 0.0127 | 7.63E-06 | 1.33247 | 0.0359488 | 1.00E-300 | -0.0427777 | 0.0096008 | 8.36E-06 | 5.38E-03 | 0.020 | 20 | 0.97 | 1373.87 |
|  |  |  |  |  |  |  |  |  |  |  |  |  |  |  |  |  |  |  |  |  |  |  |  |  |  |
| sQTL | brain | *FNBP1* | chr9:132671278:132686123:clu_78452 | 9 | 132678700 | rs10125881 | 9 | 132690663 | C | T | 0.156064 | 0.0726 | 0.0156 | 3.19E-06 | 0.432654 | 0.0473892 | 6.86E-20 | 0.167802 | 0.0404708 | 3.38E-05 | 6.72E-03 | 0.872 | 20 | 0.97 | 83.35 |
|  |  | *C9orf72* | chr9:27567162:27573785:clu_73749 | 9 | 27570474 | rs2120721 | 9 | 27566141 | G | C | 0.491054 | 0.0926 | 0.011 | 5.05E-17 | 0.16345 | 0.0259595 | 3.05E-10 | 0.566535 | 0.112362 | 4.61E-07 | 1.22E-04 | 0.012 | 20 | 0.00 | 39.64 |
|  |  |  |  |  |  |  |  |  |  |  |  |  |  |  |  |  |  |  |  |  |  |  |  |  |  |
| eQTL | blood | *USP35* | ENSG00000118369 | 11 | 77912807 | rs2511162 | 11 | 77927101 | G | A | 0.195825 | 0.0591 | 0.0146 | 5.47E-05 | 0.368467 | 0.0116514 | 1.71E-219 | 0.160394 | 0.039947 | 5.94E-05 | 8.71E-03 | 0.053 | 20 | 0.88 | 1000.09 |
|  |  | *NME4* | ENSG00000103202 | 16 | 453546 | rs6600214 | 16 | 444814 | T | C | 0.294235 | -0.055 | 0.014 | 8.46E-05 | 0.512426 | 0.00937247 | 1.00E-300 | -0.107333 | 0.0273915 | 8.91E-05 | 8.71E-03 | 0.035 | 20 | 0.75 | 2989.19 |

GWAS: genome-wide association study

mQTL: methylation quantitative trait loci

sQTL: splicing quantitative trait loci

eQTL: expression quantitative trait loci

pQTL: protein quantitative trait loci.

SMR: summary-data-based Mendelian randomization.

HEIDI: heterogeneity in dependent instruments.

Only genome-wide significant QTLs (*P* < 5E-8) are analyzed. We report significant SNP-gene combinations with FDR *P*_SMR_ < 0.01 using multiple corrections for blood mQTL probes (1902), brain mQTL probes (1929), blood sQTL probes (328), brain sQTL probes (1591), blood eQTL (489), brain eQTL probes (347), blood pQTL probes (23), and brain pQTL probes (32) , and survived after the heterogeneity test (*P*_HEIDI_> 0·01).

β in GWAS association, the regression coefficient of ALS on SNP, log(OR).

SE, standard error.

β in QTL association, the regression coefficient of DNA methylation, RNA splicing, gene expression, or protein abundance on SNP.

β in SMR association, the regression coefficient of ALS on DNA methylation, RNA splicing, gene expression, or protein abundance.

PP.H4, the posterior probability of H4, PP.H4 > 0·8 as the cut-off for the evidence of colocalization of ALS GWAS and QTL association.

F-statistic >10 indicates no evidence of weak instrumental variables.

**Supplementary Table 3.** Sensitivity analysis used the TwoSampleMR package on the association between exposures of autophagy-related genes and ALS risk.

| **QTL** | **Tissue** | **Gene** | **Exposure** | **Outcome** | **MR method** | **No. of SNP** | **OR (95% CI)** | ***P*-value** | **Correct causal**  **direction** | **Heterogeneity test** | | **Pleiotropy test** | | **Leave-one-out analyses** | **Passing sensitivity analyses** |
| --- | --- | --- | --- | --- | --- | --- | --- | --- | --- | --- | --- | --- | --- | --- | --- |
|  |  |  |  |  |  |  |  |  |  | **Cochran's Q** | ***P*** | **intercept** | ***P*** |  |  |
| mQTL | Blood | *C9orf72* | cg05990720 | ALS | Wald ratio | 1 | 0.56 (0.51-0.61) | 1.64E-40 | True | NA | NA | NA | NA | NA | Yes |
|  | Brain | *IDUA* | cg01572696 | ALS | Wald ratio | 1 | 0.96 (0.95-0.98) | 7.18E-06 | True | NA | NA | NA | NA | NA | Yes |
|  |  | *IDUA* | cg08160350 | ALS | Wald ratio | 1 | 0.96 (0.95-0.98) | 7.18E-06 | True | NA | NA | NA | NA | NA | Yes |
|  |  |  |  |  |  |  |  |  |  |  |  |  |  |  |  |
| sQTL | Brain | *FNBP1* | chr9.132671278.132686123.clu.78452 | ALS | Wald ratio | 1 | 1.18 (1.10-1.27) | 5.17E-06 | True | NA | NA | NA | NA | NA | Yes |
|  |  | *C9orf72* | chr9.27567162.27573785.clu.73749 | ALS | Wald ratio | 1 | 1.77 (1.55-2.02) | 3.82E-17 | True | NA | NA | NA | NA | NA | Yes |
|  |  |  |  |  |  |  |  |  |  |  |  |  |  |  |  |
| eQTL | Blood | *USP35* | ENSG00000118369 | ALS | Wald ratio | 1 | 1.18 (1.09-1.27) | 5.17E-05 | True | NA | NA | NA | NA | NA | Yes |
|  |  | *NME4* | ENSG00000103202 | ALS | MR Egger | 3 | 0.82 (0.67-1.00) | 2.96E-01 | True | 0.015 | 0.903 | 0.032 | 0.456 | Outlier detected | No |
|  |  |  | ENSG00000103202 | ALS | Weighted median | 3 | 0.92 (0.86-0.97) | 3.61E-03 | True | NA | NA | NA | NA |  |  |
|  |  |  | ENSG00000103202 | ALS | Inverse variance weighted | 3 | 0.92 (0.86-0.97) | 2.59E-03 | True | 1.331 | 0.514 | NA | NA |  |  |
|  |  |  | ENSG00000103202 | ALS | Simple mode | 3 | 0.97 (0.87-1.09) | 6.30E-01 | True | NA | NA | NA | NA |  |  |
|  |  |  | ENSG00000103202 | ALS | Weighted mode | 3 | 0.90 (0.84-0.96) | 7.87E-02 | True | NA | NA | NA | NA |  |  |

NA: not applicable.

THE Cochran Q statistic implemented in MR Egger and IVW method, *P* > 0·05 indicates no heterogeneity.

The intercept of MR Egger can be used to indicate whether directional horizontal pleiotropy is driving the results of MR analysis. There are no directional pleiotropies if *P* > 0·05.

The plot for leave-one-out analyses of *NME4* (blood eQTL) can be seen in Supplementary Figure 1.

**Supplementary Table 4.** Phenome-wide scan of the association between identified SNPs with other disease traits using PhenoScanner.

| **Gene** | **Tissue** | **QTL** | **Gene.**  **Chr** | **Probe** | **topSNP** | **Effect allele** | **Other allele** | **Diseases and traits** | **study** | **β** | **SE** | ***P*** | **N** | **N_cases** | **N_controls** | **Ancestry** |
| --- | --- | --- | --- | --- | --- | --- | --- | --- | --- | --- | --- | --- | --- | --- | --- | --- |
| *IDUA* | Brain | mQTL | 4 | cg08160350 | rs6599388 | C | T | Parkinson's disease | Nalls MA | -0.1484 | 0.02139 | 4.00E-12 | NA | NA | NA | European |
|  |  |  |  | cg01572696 |  |  |  |  |  |  |  |  |  |  |  |  |
| *USP35* | Blood | eQTL | 11 | ENSG00000118369 | rs2511162 | A | G | Impedance of arm left | Neale B | -0.01897 | 0.002311 | 2.26E-16 | 331292 | 0 | 331292 | European |
|  |  |  |  |  |  |  |  | Impedance of arm right | Neale B | -0.02043 | 0.002302 | 6.93E-19 | 331279 | 0 | 331279 | European |
|  |  |  |  |  |  |  |  | Impedance of whole body | Neale B | -0.01495 | 0.002496 | 2.11E-09 | 331284 | 0 | 331284 | European |
|  |  |  |  |  |  |  |  | Age at menarche | ReproGen | -0.044 | 0.0077 | 1.10E-08 | 182416 | 0 | 182416 | European |

NA: not applicable.

**Supplementary Table 5.** SMR results of the association between DNA methylation/RNA splicing and expression of autophagy-related genes.

| **Tissue** | **Expo QTL** | **Outco QTL** | **Expo**  **Gene** | **Expo Chr.** | **Expo Probe** | **Expo**  **base pair** | **Outco**  **Gene** | **Outco Chr.** | **Outco Probe** | **Outco base pair** | **topSNP** | **SNP Chr.** | **SNP base pair** | **Effect allele** | **Other allele** | **Effect allele frequency** | **eQTL association** | | | **mQTL/sQTL association** | | | **SMR association** | | | | **HEIDI Test** | |
| --- | --- | --- | --- | --- | --- | --- | --- | --- | --- | --- | --- | --- | --- | --- | --- | --- | --- | --- | --- | --- | --- | --- | --- | --- | --- | --- | --- | --- |
|  |  |  |  |  |  |  |  |  |  |  |  |  |  |  |  |  | **β** | **SE** | ***P*** | **β** | **SE** | ***P*** | **β** | **SE** | ***P*** | **FDR *P*** | ***P*** | **No. of SNPs** |
| Blood | mQTL | eQTL | *FNBP1* | 9 | cg14364797 | 132651576 | *FNBP1* | 9 | ENSG00000187239 | 132727469 | rs151267204 | 9 | 132594774 | A | G | 0.0139165 | 0.593185 | 0.0302667 | 1.59E-85 | 0.637646 | 0.114378 | 2.48E-08 | 0.930272 | 0.173487 | 8.22E-08 | 3.34E-07 | 0.067 | 17 |
| Brain | mQTL | eQTL | *FNBP1* | 9 | cg13399952 | 132652889 | *FNBP1* | 9 | ENSG00000187239 | 132727470 | rs10739762 | 9 | 132655652 | T | C | 0.481113 | 0.241174 | 0.0321471 | 6.28E-14 | -0.261974 | 0.0435156 | 1.74E-09 | -0.920603 | 0.196067 | 2.66E-06 | 1.84E-05 | 0.021 | 5 |
| Blood | mQTL | eQTL | *USP35* | 11 | cg25473794 | 77921181 | *USP35* | 11 | ENSG00000118369 | 77912807 | rs2512525 | 11 | 77923019 | C | T | 0.195825 | 0.327741 | 0.011417 | 3.17E-181 | -0.509779 | 0.0431931 | 3.80E-32 | -0.642909 | 0.0588974 | 9.69E-28 | 1.68E-26 | 0.028 | 20 |
| Brain | mQTL | eQTL | *USP35* | 11 | cg02537108 | 77907224 | *USP35* | 11 | ENSG00000118369 | 77913068 | rs682742 | 11 | 77843259 | T | C | 0.630219 | 0.141814 | 0.0280409 | 4.25E-07 | 0.347918 | 0.0630137 | 3.36E-08 | 0.407606 | 0.109297 | 1.92E-04 | 9.41E-04 | 0.436 | 10 |
| Brain | mQTL | eQTL | *USP35* | 11 | cg21167761 | 77903617 | *USP35* | 11 | ENSG00000118369 | 77913068 | rs10793289 | 11 | 77850517 | C | A | 0.536779 | -0.134763 | 0.0279068 | 1.37E-06 | 0.596301 | 0.0580292 | 9.05E-25 | -0.225998 | 0.0517099 | 1.24E-05 | 7.44E-05 | 0.012 | 14 |
| Brain | sQTL | eQTL | *IDUA* | 4 | chr4:997900:998054:clu_294701_ | 997977 | *IDUA* | 4 | ENSG00000127415 | 989568 | rs6814642 | 4 | 978388 | A | G | 0.361829 | -0.253567 | 0.0371936 | 9.26E-12 | 0.153152 | 0.0275725 | 2.78E-08 | -1.65565 | 0.38448 | 1.66E-05 | 5.50E-05 | 0.355 | 20 |

eQTL: expression quantitative trait loci.

mQTL: methylation quantitative trait loci.

sQTL: splicing quantitative trait loci.

SMR: summary-data-based Mendelian randomization.

HEIDI: heterogeneity in dependent instruments.

Only genome-wide significant mQTL/sQTLs (*P* < 5E-8) are analyzed. We report significant SNP-gene combinations with FDR *P*_SMR_ < 0.01 using multiple corrections for blood mQTL probes (4103), brain mQTL probes (3853), blood sQTL probes (665), and brain sQTL probes (3185) , and survived after the heterogeneity test (*P*_HEIDI_ > 0·01).

SE, standard error.

β in QTL association, the regression coefficient of DNA methylation/RNA splicing on SNP.

β in SMR association, the regression coefficient of DNA methylation/RNA splicing of gene expression.

**Supplementary Table 6.** Cell-type-specific MR analyses of identified causal autophagy-related genes.

| **QTL** | **Cell type** | **Gene** | **Exposure** | **Outcome** | **MR Method** | **No. of SNP** | **OR (95% CI)** | ***P*-value** | **Correct causal direction** | **Heterogeneity test** |  | **Pleiotropy test** |  | **Leave-one-out analyses** |
| --- | --- | --- | --- | --- | --- | --- | --- | --- | --- | --- | --- | --- | --- | --- |
|  |  |  |  |  |  |  |  |  |  | **Cochran's Q** | ***P*** | **intercept** | ***P*** |  |
| eQTL | Astrocytes | *FNBP1* | Astrocytes_*FNBP1* | ALS | Wald ratio | 1 | 0.88(0.81-0.94) | 3.99E-04 | TRUE | NA | NA | NA | NA | NA |
| eQTL | Astrocytes | *C9orf72* | Astrocytes_*C9orf72* | ALS | Wald ratio | 1 | 1.25(1.151-1.36) | 1.33E-07 | TRUE | NA | NA | NA | NA | NA |

**Supplementary Table 7.** Known ALS causative/risk genes from ALSoD.

| **Gene symbol** | **Gene name** | **Category** |
| --- | --- | --- |
| *ANXA11* | annexin A11 | Definitive ALS gene |
| *C9orf72* | C9orf72-SMCR8 complex subunit | Definitive ALS gene |
| *CHCHD10* | coiled-coil-helix-coiled-coil-helix domain containing 10 | Definitive ALS gene |
| *EPHA4* | EPH receptor A4 | Definitive ALS gene |
| *FUS* | FUS RNA binding protein | Definitive ALS gene |
| *HNRNPA1* | heterogeneous nuclear ribonucleoprotein A1 | Definitive ALS gene |
| *KIF5A* | kinesin family member 5A | Definitive ALS gene |
| *NEK1* | NIMA related kinase 1 | Definitive ALS gene |
| *OPTN* | optineurin | Definitive ALS gene |
| *PFN1* | profilin 1 | Definitive ALS gene |
| *SOD1* | superoxide dismutase 1 | Definitive ALS gene |
| *TARDBP* | TAR DNA binding protein | Definitive ALS gene |
| *TBK1* | TANK binding kinase 1 | Definitive ALS gene |
| *UBQLN2* | ubiquilin 2 | Definitive ALS gene |
| *UNC13A* | unc-13 homolog A | Definitive ALS gene |
| *VAPB* | VAMP associated protein B and C | Definitive ALS gene |
| *VCP* | valosin containing protein | Definitive ALS gene |
| *ATXN2* | ataxin 2 | Clinical modifier |
| *CAMTA1* | Calmodulin brinding transcriptions activator 1 | Clinical modifier |
| *ENAH* | ENAH actin regulator | Clinical modifier |
| *ATXN1* | ataxin 1 | Strong evidence |
| *CCNF* | cyclin F | Strong evidence |
| *CFAP410* | Cilia and Flagella Associated Protein 410 | Strong evidence |
| *HFE* | homeostatic iron regulator | Strong evidence |
| *NIPA1* | NIPA magnesium transporter 1 | Strong evidence |
| *SCFD1* | sec1 family domain containing 1 | Strong evidence |
| *TUBA4A* | tubulin alpha 4a | Strong evidence |
| *ANG* | angiogenin | Moderate evidence |
| *ARHGEF28* | rho guanine nucleotide exchange factor 28 | Moderate evidence |
| *CDH22* | cadherin 22 | Moderate evidence |
| *CHMP2B* | charged multivesicular body protein 2B | Moderate evidence |
| *CNTN6* | contactin 6 | Moderate evidence |
| *CRYM* | crystallin mu | Moderate evidence |
| *CSNK1G3* | casein kinase 1 gamma 3 | Moderate evidence |
| *CX3CR1* | C-X3-C motif chemokine receptor 1 | Moderate evidence |
| *DAO* | D-amino acid oxidase | Moderate evidence |
| *DNAJC7* | DnaJ heat shock protein family (Hsp40) member C7 | Moderate evidence |
| *DNMT3A* | DNA methyltransferase 3 alpha | Moderate evidence |
| *ERBB4* | erb-b2 receptor tyrosine kinase 4 | Moderate evidence |
| *FIG4* | FIG4 phosphoinositide 5-phosphatase | Moderate evidence |
| *GLE1* | GLE1 RNA export mediator | Moderate evidence |
| *GPX3* | glutathione peroxidase 3 | Moderate evidence |
| *LMNB1* | lamin B1 | Moderate evidence |
| *SARM1* | sterile alpha and TIR motif containing 1 | Moderate evidence |
| *SMN1* | survival of motor neuron 1, telomeric | Moderate evidence |
| *SQSTM1* | sequestosome 1 | Moderate evidence |
| *SS18L1* | SS18L1 subunit of BAF chromatin remodeling complex | Moderate evidence |
| *TNIP1* | TNFAIP3 interacting protein 1 | Moderate evidence |

ALSoD: Amyotrophic Lateral Sclerosis online Database (https://alsod.ac.uk/).

**Supplementary Table 8.** Druggability of the causal autophagy-related genes for ALS.

| **Search term** | **Match term** | **Match type** | **Category** | **Sources** |
| --- | --- | --- | --- | --- |
| ENSG00000118369 | *USP35* | Definite | ENZYME | Pharos |
| ENSG00000118369 | *USP35* | Definite | PROTEASE | dGene |
| ENSG00000187239 | *FNBP1* | Definite | CLINICALLY ACTIONABLE | FoundationOneGenes|CarisMolecularIntelligence |
| ENSG00000127415 | *IDUA* | Definite | ENZYME | Pharos|HumanProteinAtlas |
| ENSG00000147894 | *C9ORF72* | Definite | KINASE | Pharos |

**Supplementary Figure 1.** Leave-one-out sensitivity analyses of the SNPs represented the blood expression of *NME4* and ALS risk.


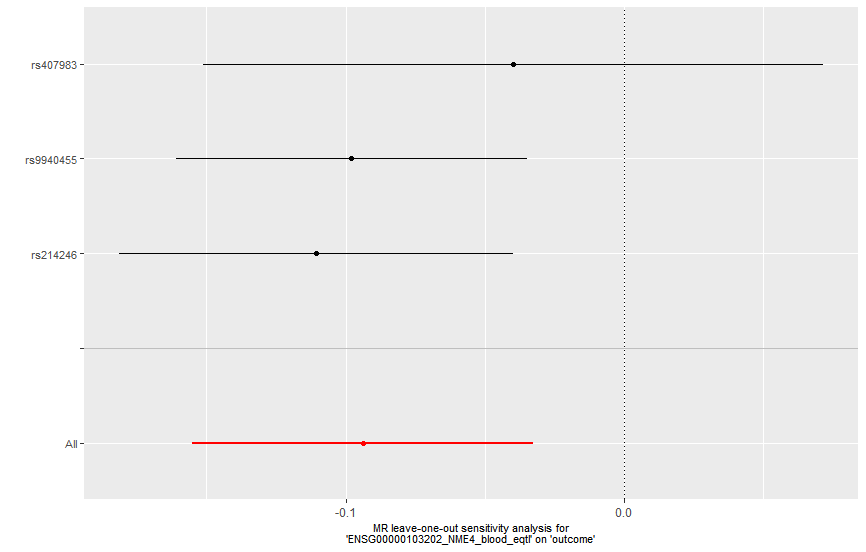


The estimated causal effect is shown for each excluded SNP, and the overall estimate using all the SNPs is shown in red. The error bars represent the 95% confidence intervals.

**References**

1. Zhu Z, Zhang F, Hu H, Bakshi A, Robinson MR, Powell JE, Montgomery GW, Goddard ME, Wray NR, Visscher PM and Yang J. Integration of summary data from GWAS and eQTL studies predicts complex trait gene targets. *Nat Genet*. 2016;48:481-7.

2. Genomes Project C, Auton A, Brooks LD, Durbin RM, Garrison EP, Kang HM, Korbel JO, Marchini JL, McCarthy S, McVean GA and Abecasis GR. A global reference for human genetic variation. *Nature*. 2015;526:68-74.

3. Wu Y, Zeng J, Zhang F, Zhu Z, Qi T, Zheng Z, Lloyd-Jones LR, Marioni RE, Martin NG, Montgomery GW, Deary IJ, Wray NR, Visscher PM, McRae AF and Yang J. Integrative analysis of omics summary data reveals putative mechanisms underlying complex traits. *Nat Commun*. 2018;9:918.

4. Giambartolomei C, Vukcevic D, Schadt EE, Franke L, Hingorani AD, Wallace C and Plagnol V. Bayesian test for colocalisation between pairs of genetic association studies using summary statistics. *PLoS Genet*. 2014;10:e1004383.
